# Structural Investigations of Phthalazinone Derivatives as Allosteric Inhibitors of Human DNA Methyltransferase 3A

**DOI:** 10.1021/acsmedchemlett.3c00528

**Published:** 2024-04-08

**Authors:** Ivan Hernandez, Ethan Ward, Thomas R. R. Pettus, Norbert O. Reich

**Affiliations:** † Department of Chemistry and Biochemistry, University of California, Santa Barbara, California 93106-9510, United States; ‡ Biomolecular Science and Engineering, University of California, Santa Barbara, California 93106-9510, United States

**Keywords:** Allosteric inhibitor, DNMT3A, SAR, Phthalazinone derivatives, Acute myeloid leukemia

## Abstract

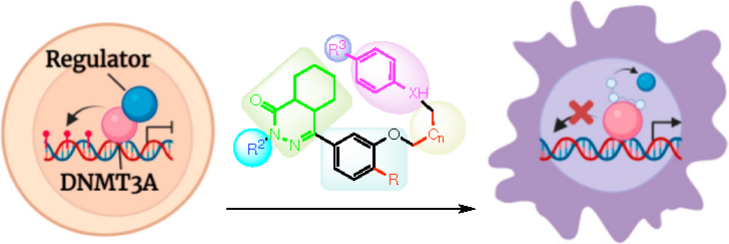

The development of new therapeutics targeting enzymes involved in epigenetic pathways such as histone modification and DNA methylation has received a lot of attention, particularly for targeting diverse cancers. Unfortunately, irreversible nucleoside inhibitors (azacytidine and decitabine) have proven highly cytotoxic, and competitive inhibitors are also problematic. This work describes synthetic and structural investigations of a new class of allosteric DNA methyltransferase 3A (DNMT3A) inhibitors, leading to the identification of several critical pharmacophores in the lead structure. Specifically, we find that the tetrazole and phthalazinone moieties are indispensable for the inhibitory activity of DNMT3A and elucidate other modifiable regions in the lead compound.

Enzymes involved in epigenetic regulation often have essential roles in cellular processes, including development, differentiation, and cell cycle regulation.^1−3^ The dynamic nature of epigenetic regulation involves intricate communication between various players, including readers, writers, erasers, and transcription factors, to modulate gene expression.^4,5^ Among these players, DNMT3A stands out as a central regulator, and not surprisingly, DNMT3A mutations are key drivers for diverse cancers.^6^ For example, DNMT3A is the most frequently mutated gene in acute myeloid leukemia (AML).^6^ The mutations are concentrated at interfaces that stabilize the DNMT3A homotetramer as well as heterotetramers, resulting in aberrant methylation patterns caused by dramatic reductions in the ability of DNMT3A to processively methylate DNA.^7^ Current FDA-approved drugs targeting DNMT3A (azacytidine and decitabine) have limitations due to their incorporation into DNA, which leads to unintended effects and limited effectiveness while also posing significant cytotoxicity risks.^8−10^ As a result, there is growing interest in developing novel DNMT3A inhibitors with alternative mechanisms that can offer improved patient outcomes.^11−16^

We recently reported a screening effort surveying an open-source chemical library derived from the Medicines for Malaria Venture (MMV) Pathogen Box. Twelve compounds showed greater than 90% inhibition of DNMT3A at 60 μM. The three most potent compounds were identified as hydrophthalazinone **1a** and its five-membered-ring derivative **1b** (Figure 1)^17^ along with a familiar phenylurea known as suramin. The latter compound was omitted from further studies due to our familiarity with its indiscriminate enzyme inhibitory activity. As lead structures, compounds **1a** and **1b** both provided numerous hydrogen-bond donor and acceptor sites. In addition, their tetrazole moieties are known to exist in a nearly 1:1 ratio of 1*H* and 2*H* tautomeric forms,^18^ potentially serving as distinct bioisosteres of different carboxylic acids.^19^

**Figure 1:**
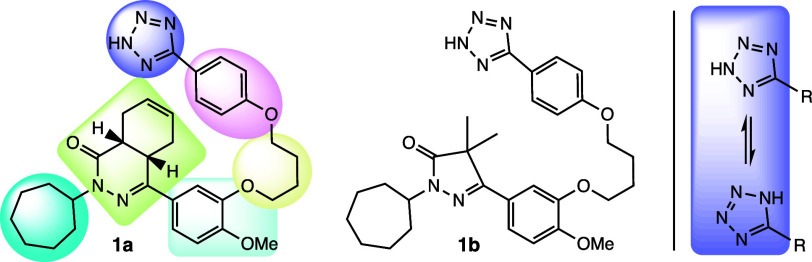
Lead DNMT3A allosteric inhibitors **1a** and **1b** and potential structural zones for further refinement and improvement of inhibition by **1a**.

Earlier studies with these compounds were aimed at identifying their mechanisms of action. Compound **1a** showed a *K*_i_ of 9.16–18.85 μM with *S*-adenosyl methionine (SAM, AdoMet), whereas compound **1b** showed a *K*_i_ of 3.70–7.06 μM with SAM; both inhibitors were found to act allosterically.^17^ In a subsequent publication, these two compounds (**1a** and **1b**) were reported as first-in-class allosteric inhibitors of DNMT3A, which act by disrupting protein–protein interactions (PPIs) and induce acute myeloid leukemia cell differentiation (Figure 2).^20^ Importantly, compounds **1a** and **1b** are significantly less cytotoxic than FDA-approved inhibitors.^21^

**Figure 2:**
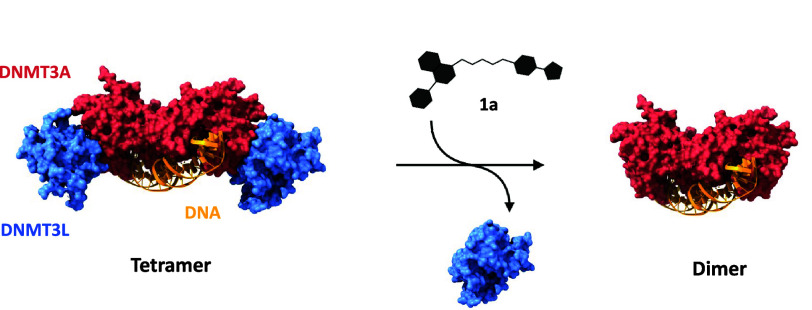
Structure of DNMT3A–DNMT3L in complex with DNA (PDB entry 5YX2); compound **1a** interferes with the PPIs of DNMT3A and its regulatory partners. Binding of compound **1a** reduces DNMT3A tetramers to dimers, disabling processive DNA methylation and decreasing enzyme activity.

In view of these biological and chemical properties and the increasing need for small-molecule inhibitors of DNMT3A with new mechanisms of action, we concluded that compounds **1a** and **1b** and their unique allosteric mechanism offered an exceptionally fertile terrain for continued chemical prospecting. Herein we report on our efforts to identify the features of compounds **1a** and **1b** which are essential for their inhibitory activity.

Compounds **1a** and **1b** had been previously synthesized and investigated by Timmerman in 2001 as potential therapeutics for African sleeping sickness due to their selective, albeit unrelated, inhibition of cyclic nucleotide phosphodiesterase (PDE) enzymes found in *Trypanosoma brucei*.^22^ His prior strategy was readily amenable to various structural modifications for future synthetic pursuits, particularly within the six colored zones indicated within the structure shown in Figure 3, which encompass the lead compounds. However, the six-membered skeleton **1a** clearly offered more options for perturbation than the corresponding five-membered analog **1b**.

**Figure 3:**
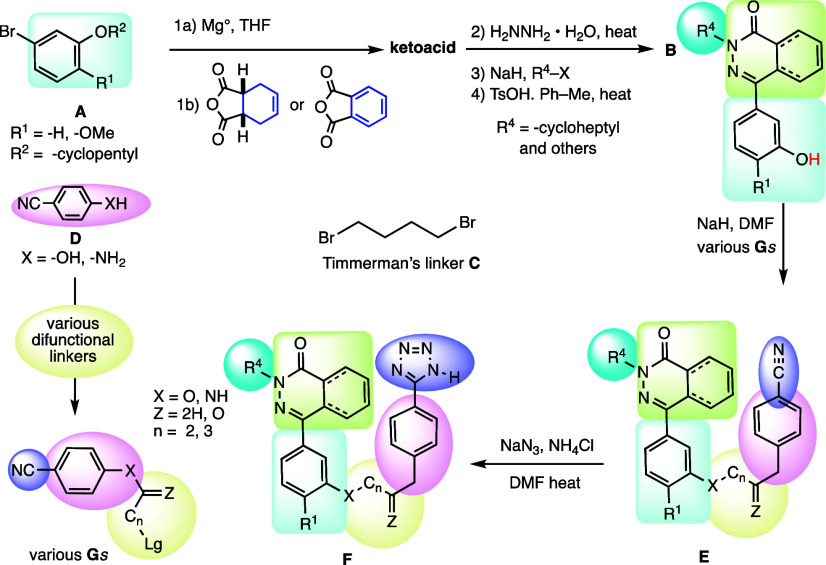
Timmerman’s original 2001 synthetic route to compounds **1a**, **1b**, **2**, and **3** converted **A** to **B**, added the difunctional linker **C** and then phenol **D** to arrive at **E**, which was converted to **F**. For other compounds, we instead chose to couple **B** with various linkers to provide **G** and thereby increase synthetic convergence.

Timmerman’s **1a**, **1b**, **2**, and **3** all displayed a four-carbon linker. The syntheses of these had begun with the requisite aryl bromide **A**. This material was sequentially converted to the desired keto acid and then to the corresponding phthalazinone derivative **B**. Further introduction of linker **C** and phenol **D** provided nitrile **E**, whereupon the fragile acidic tetrazole motif shown in **F** was introduced by cycloaddition. To determine the compound’s IC_50_ with DNMT3A, we began by resynthesizing the lead structure **1a** using the identical route and observed an IC_50_ of 14 ± 2 μM (Figures 4 and S1). Next, we examined the nitrile precursor **2**, which was the penultimate intermediate leading to compound **1a**. Compound **2** (destetrazole) exhibited an IC_50_ of >300 μM toward DNMT3A. Thus, without the tetrazole, which likely serves as a hydrogen-bond donor, compound **2** proved only ≤ 4% as potent as the lead compound **1a**. We next prepared carboxylic acid **3** according to the Timmerman protocol and discovered that it was 60% as potent as the lead compound [IC_50_ = 23 μM]. When considered together, these three results indicated that the acidic hydrogens in the tetrazole moiety of **1a** and carboxylic acid **3** are critical for inhibition, presumably serving as hydrogen-bond donors—functionality which is absent from nitrile **2**.

**Figure 4:**
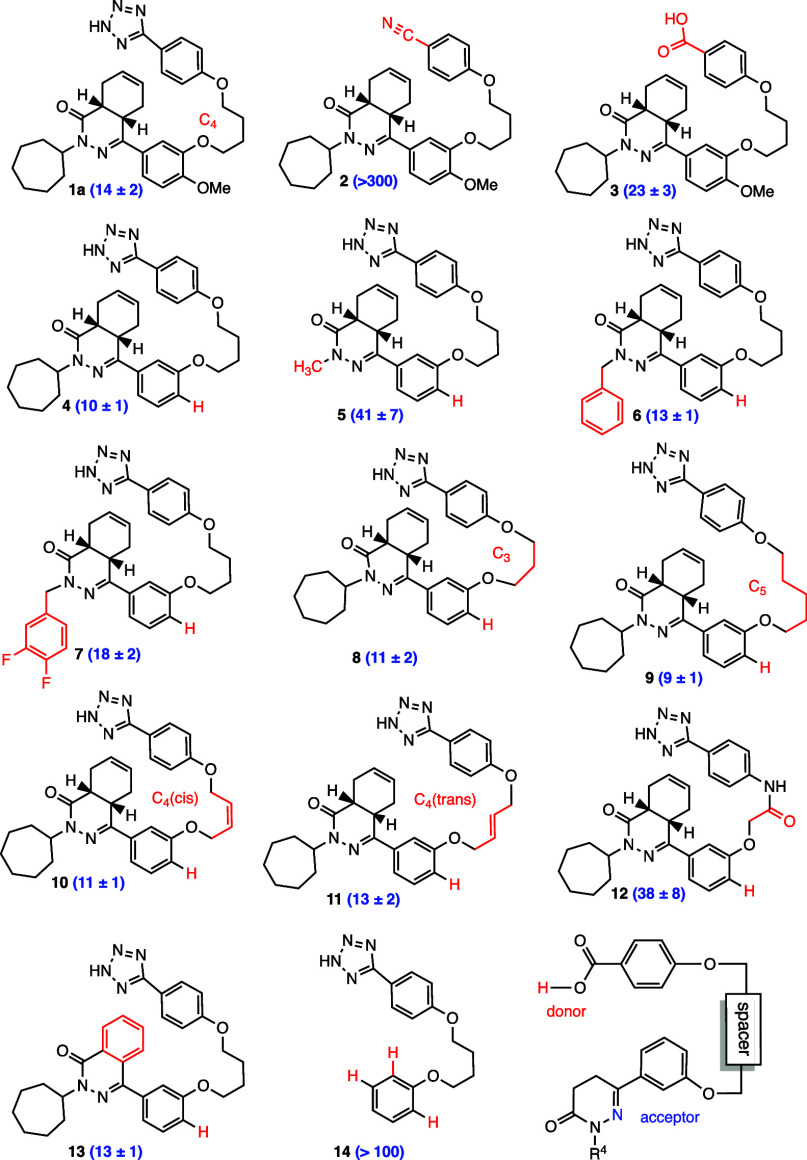
Derivatives tested and respective IC_50_ values (μM) with errors. Compounds **1a**, **2**, and **3** were previously synthesized by Timmerman.

We next prepared and studied compounds not previously synthesized but readily accessible through Timmerman’s general synthetic strategy, but diverging somewhat from his strategy. Removal of the aryl methoxy residue in the starting brominated aromatic sped construction of analogs by removing protecting group regimes and presumably provided additional degrees of freedom of rotation about the neighboring aliphatic C–O bond. Thus, we were very gratified to find that the desmethoxy compound **4** [IC_50_ = 10 μM] resulted in a slight increase in potency (140% of **1a**). In view of this finding, we chose to continue the exploration of chemical space with this simpler scaffold.

Next, we focused on modifications of the R^4^ substituent. Replacing the Timmerman seven-membered ring in analogue **4** with the smaller methyl residue in compound **5** resulted in decreased potency (IC_50_ = 41 μM, 34% of **1a**). Utilization of a benzyl residue as R^4^ provided activity closer to the initial lead structure (compound **6**: IC_50_ = 13 μM, 107% of **1a**). Thereafter, we chose to examine the bisfluorinated compound **7** and found that its potency had decreased (IC_50_ = 18 μM, 77% of **1a**). When considered together, these three R^4^ modifications indicated that this residue was likely positioned either in a lipophilic region or adjacent to an aqueous interface, as both scenarios would be expected to lead to greater potency when a hydrophobic substituent is present.^23^

Our attention next turned toward perturbations within the linker interconnecting the two aryl motifs. Replacement of the original C_4_ intervening chain with a C_3_ chain provided a slight increase in potency (compound **8**: IC_50_ = 11 μM, 127% of **1a**), whereas replacement of the C_4_ chain with a C_5_ residue led to further improvement (compound **9**: IC_50_ = 9 μM, 155% of **1a**). Remarkably, introduction of a *cis* double bond into the C_4_ linkage (compound **10**: IC_50_ = 11 μM, 127% of **1a**), as compared with introduction of a C_4_*trans* linkage (compound **11**: IC_50_ = 13 μM, 107% of **1a**) resulted in similar outcomes. Indeed, the two-carbon linker in the amide derivative **12** provided inhibition [IC_50_ = 38 μM] that was 36% of that of the initial lead structure **1a**. When pondered together, these five linker modifications indicate that this chain likely folds back upon itself and does not interact with any protein residues. However, the decrease in potency seen from **12** compared to the other linker derivatives indicates that the linker chain is proximal to a hydrophobic region of DNMT3A. Next, we examined deshydrophthalazinone derivative **14**, which without its potential hydrogen-bond acceptor displayed reduced activity with an IC_50_ of >100 μM, ≤14% of the inhibition of **1a**. This result indicated that a lone pair of either the carbonyl moiety or the phthalazinone residue is important in interacting with a proton donor and is critical for allosteric inhibition. We then examined the flat achiral phthalazinone derivative **13**. It is inhibitory activity (IC_50_ = 13 μM) was nearly identical to that of the initial lead compound **1a**, demonstrating that whatever allosteric binding interface had accommodated the *cis-*fused cyclohexane of **1a** also tolerated a robust, flat, and less conformationally mobile aromatic ring. Moreover, the absence of increased potency with the presumably more electron-rich phthalazinone carbonyl leads us to speculate that the carbonyl oxygen atom does *not* serve as the principal hydrogen-bond acceptor for this motif.

To identify potential binding sites of compound **1a** on DNMT3A, docking studies were conducted using AutoDock Vina. By minimizing the free energy of the inhibitor–DNMT3A complex, we identified the most energetically favorable pose in silico, which showed compound **1a** bound near the tetramer interface (Figure 5A). In its binding conformation, the compound occupied a location adjacent to the tetramer interface, providing distinctive pockets for the tetrazole and phthalazinone moieties connected by a folded linker chain (Figure 5B). This orientation supports our functional data with linker analogs and suggests that residues Q606 and R742 may interact with the tetrazole and carbonyl of **1a**, respectively (Figure 5C). Although this binding site is not a critical part of the tetramer interface, the binding of compound **1a** could potentially modulate the orientations and reactivity of other pivotal residues essential for PPIs.

**Figure 5:**
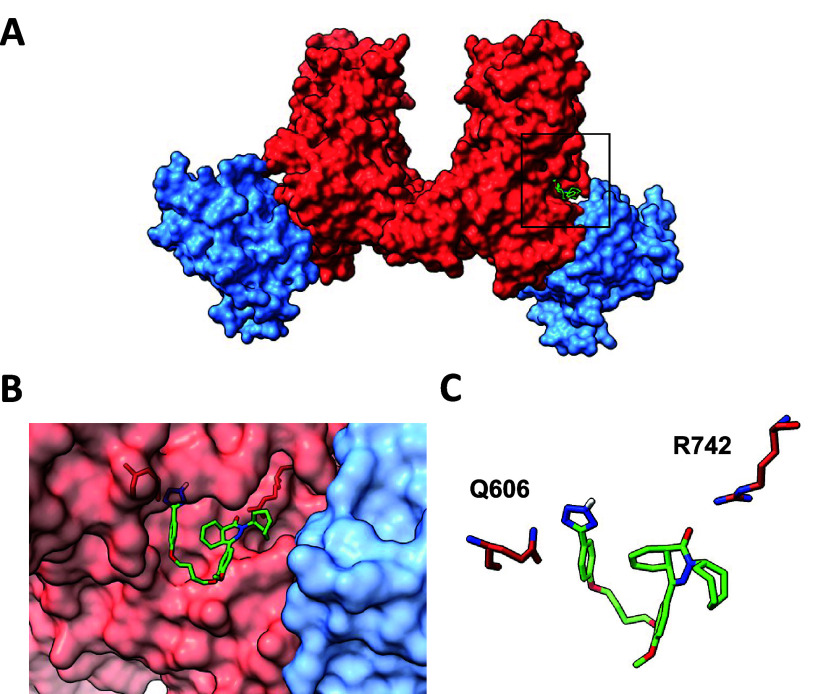
(A) Docking studies with compound **1a** indicate that it and its analogs bind to the DNMT3A homotetramer on the subunit, which is also bound to DNA near the tetramer interface (PDB entry 5yx2). (B) Close-up view showing the binding pocket of compound **1a**. (C) This binding pose implicates potential interactions of the tetrazole and phthalazinone carbonyl with residues Q606 and R742, respectively.

We then sought to determine whether selected analogs of **1a** (**3**, **4**, **5**, and **10**) also displayed allosteric forms of inhibition. Using methylation assays with and without inhibitor and varied concentrations of DNA or SAM, we generated nonlinear Michaelis–Menten curves with corresponding double-reciprocal plots (Figure S2) and fit them to classical inhibition models. All inhibitors best fit a mixed-type inhibition model with DNA and SAM (Table S1), meaning that the inhibitor binds both the free enzyme and enzyme–substrate complex with different affinities. By analyzing double-reciprocal plots, this mechanistic model was confirmed because the changes in *y*-intercept and slope are inconsistent with competitive and uncompetitive forms of inhibition, respectively. Furthermore, the linear regressions converge at, below, and above the *x*-axis on double-reciprocal plots between compounds and/or the substrate being varied, indicating that the affinity of the inhibitors for the free enzyme and enzyme–substrate complex can be modified with small changes to the inhibitor structure. Extraction of the kinetic parameter α from the mixed-type model (Figure S3) revealed that compound **1a** has a preference toward the enzyme–substrate complex (α < 1) with both DNA and SAM (Table S2). Furthermore, the selected analogs also bind more tightly to the enzyme–substrate complex than to the free enzyme with both DNA and SAM (Table S2). This preference could be beneficial since DNMT3A bound to DNA is likely the dominant species in a cellular context.

This study provides key insights into the pharmacophores of compound **1a** for DNMT3A, which itself is significantly improved over currently used drugs to treat AML.^20^ By synthesizing a set of 13 derivative compounds, we showed that the tetrazole and phthalazinone moieties are critical for inhibitory activity. Moreover, the elimination of a lipophilic R^4^ moiety led to a decrease in potency. Furthermore, docking studies predict that residues Q606 and R742 have interactions with **1a**. Additionally, our mechanistic investigations showed that **1a** and four derivatives display a mixed-type inhibition mechanism with a general preference for the enzyme–substrate complex. Taken together, these findings provide a scaffold for further optimization of compound **1a**.

## Supporting Information Available

Full details of all materials and methods used in this work, including protein production, assay methods, and chemical synthesis; full spectroscopic data for compounds **1a**–**14** along with procedures; and ^1^H and ^13^C NMR data for all new derivatives (PDF)

## Abbreviations

DNMT3A: DNA methyltransferase 3A
AML: acute myeloid leukemia
MMV: Medicines for Malaria Venture
PPI: protein–protein interaction
SAM: *S*-adenosyl methionine
PDE: phosphodiesterase
