# Supplementary material for: Structural Investigations of Phthalazinone Derivatives as Allosteric Inhibitors of Human DNA Methyltransferase 3A
Source: ACS Med Chem Lett. 2024 Apr 8;15(5):590–4. doi: 10.1021/acsmedchemlett.3c00528 (PMC11089561; doi:10.1021/acsmedchemlett.3c00528)
Supplement: Supplementary file 1 — ml3c00528_si_001.pdf [file ml3c00528_si_001.pdf]

Supporting Information

Structural Investigations of Phthalazinone Derivatives as Allosteric Inhibitors of Human DNA Methyltransferase 3A

Ivan Hernandez, Ethan Ward, Thomas R. R. Pettus,[*a] and Norbert O. Reich[*a,b]

Department of Chemistry and Biochemistry, University of California, Santa Barbara
Santa Barbara, California 93106-9510 (United States)

Table of Contents

1) Biochemical Methods S1-S2
2) IC_{50} Determination Curves
   a. Figure S1. Dose response curves for compounds **1a-14** S3
3) Mechanism Studies
   a. Figure S2. Double reciprocal plots of compounds **1a**, **3**, **4**, **5**, and **10** S4
   b. Figure S3. Kinetic scheme of mixed-type inhibitors S5
   c. Table S1. R^2 fitting comparison of kinetic studies to inhibition models S5
   d. Table S2. Extracted α values from mixed-type inhibition model S5
4) General Methods of Synthetic Chemistry S6
5) Chemical Synthesis and Compound Characterization of Compounds **1a-14** S6-S27
6) Chemical Synthesis and Compound Characterization of Difunctional Linkers. S27-S32
7) Computational Docking S32
8) NMR Spectra for Compounds **4-14** S32-S53
9) References S54

1. Biochemical Methods

Expression and Purification of DNMT3A_CD

The catalytic domain of DNMT3A (residues 634-912) were used for all experiments since the catalytic domain and the full-length enzyme have comparable kinetic parameters. DNMT3A_CD was expressed in NiCo21 (DE3) cells (NEB) using a codon optimized plasmid pET28a-hDNMT3A_CD (Δ1-611). Cell cultures were grown in LB medium at 37°C to an OD_{600} = 0.8. Cultures were cooled to 25°C, then expression was induced by the addition of 1 mM IPTG (Gold Biotechnology). Cells were harvested by centrifugation after 5 hours and flash frozen in liquid nitrogen for storage at -80°C.

Cells were resuspended in lysis buffer containing 50 mM K_2HPO_4/KH_2PO_4, 500 mM NaCl, 50 mM imidazole, 10% glycerol [v/v], 1 mM EDTA and 1 mM PMSF at pH 7.8. Sonication was used for lysis and the supernatant was collected after centrifugation at 18,000 x g for 1 hour. Lysates were applied to an ÄKTA start FPLC system (GE healthcare) and loaded onto a 1 mL HisTrap Excel column (Cytiva). The column was washed with 50 mL of lysis

buffer and eluted with an identical buffer containing a higher imidazole concentration of 500 mM. Eluted protein was desalted with a 5 mL HiTrap Desalting column (Cytiva) and flash frozen in liquid nitrogen for storage at -80°C. DNMT3A_CD concentration was determined using UV-vis with an extinction coefficient determined by protparam of 38,180 M^{-1} cm^{-1} to represent the monomeric state.

In Vitro Methylation Assays

Methylation assays were used to determine nanomolar amounts of methylated DNA product. Reactions were carried out in 50 mM K_2HPO_4/KH_2PO_4 at pH 7.8, 1 mM EDTA, 1 mM DTT, 0.2 mg/mL BSA, 20 mM NaCl, 150 nM DNMT3A_CD, 10 uM poly dI-dC (Sigma Aldrich), and 10 uM SAM (Sigma Aldrich) unless otherwise stated. The SAM cofactor was diluted with [^{3}H]-SAM from PerkinElmer in 10 mM H_2SO_4. All inhibitors were dissolved and diluted in DMSO prior to addition to methylation reactions. Reactions with and without inhibitors had a final DMSO concentration of 10% [v/v]. All reactions containing inhibitors were preincubated with DNMT3A and inhibitor for 1 hour. Reactions were activated with poly dI-dC to a final volume of 20 uL, then incubated at 37°C for 1 hour. Reaction aliquots were spotted on Hybond-N+ membranes (GE healthcare), then washed with 50 mM K_2HPO_4/KH_2PO_4 at pH 7.8 and ethanol. Membranes were dried and counted using a Hidex LS 300 scintillation counter.

Kinetics Studies

In vitro methylation assays were performed as described above using two concentrations of inhibitor (0 uM and ~IC_{50}) and varied concentrations of substrate SAM or poly dI-dC while the other substrate is held at a saturating concentration of 10 uM. For the variation of SAM, concentrations of 0.15, 0.25, 0.5, 1, 3 and 10 uM were used, while for the variation of poly dI-dC concentrations of 0.15, 0.25, 0.5, 1, 2.5, and 10 uM were used. Data was fitted globally with *Prism 10* and best-fit inhibition models were determined using an F-test ($p < 0.0001$). Lineweaver-Burke plots were generated and fit to a linear regression model. Data represent the mean and standard deviation of three independent reactions.

Determination of IC_{50} Value

Percent activity of methylation assays was determined by comparing activities of assays with inhibitor to assays without inhibitor. Sigmoidal dose-response curves of percent activity vs compound concentration were fitted in *Prism 10*. The data was best-fit using Hill slope coefficient constrained to a value of -2. All assays were performed in triplicate, with additional repetitions added as necessary.

Safety Statement

No unexpected or unusually high safety hazards were encountered during biochemical experimentation.

2. IC_{50} Determination Curves

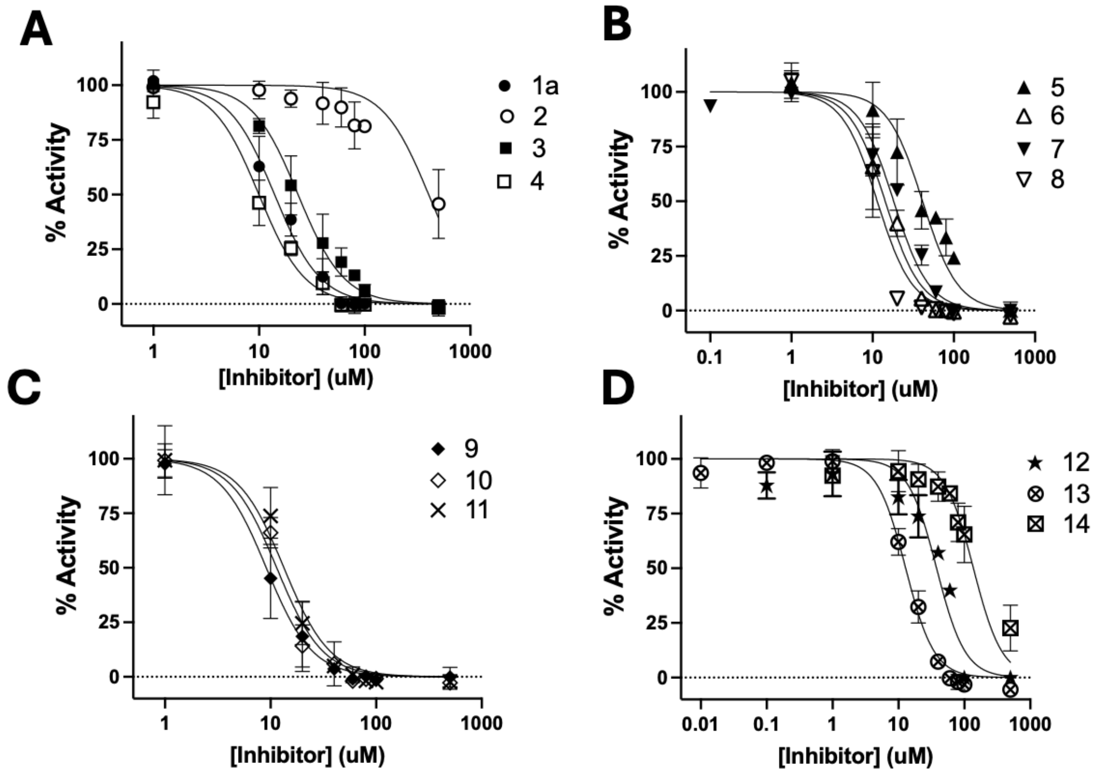

Figure S1: Dose response curves IDH compounds **1a-14** (**A** = **1-4**, **B** = **5-8**, **C** = **9-11**, **D** = **12-14**). Reactions consisted of 150 nM DNMT3A, 10 uM SAM, and 10 uM poly dI-dC. The data points represent the mean and standard deviation of three independent reactions.

3. Mechanism Studies

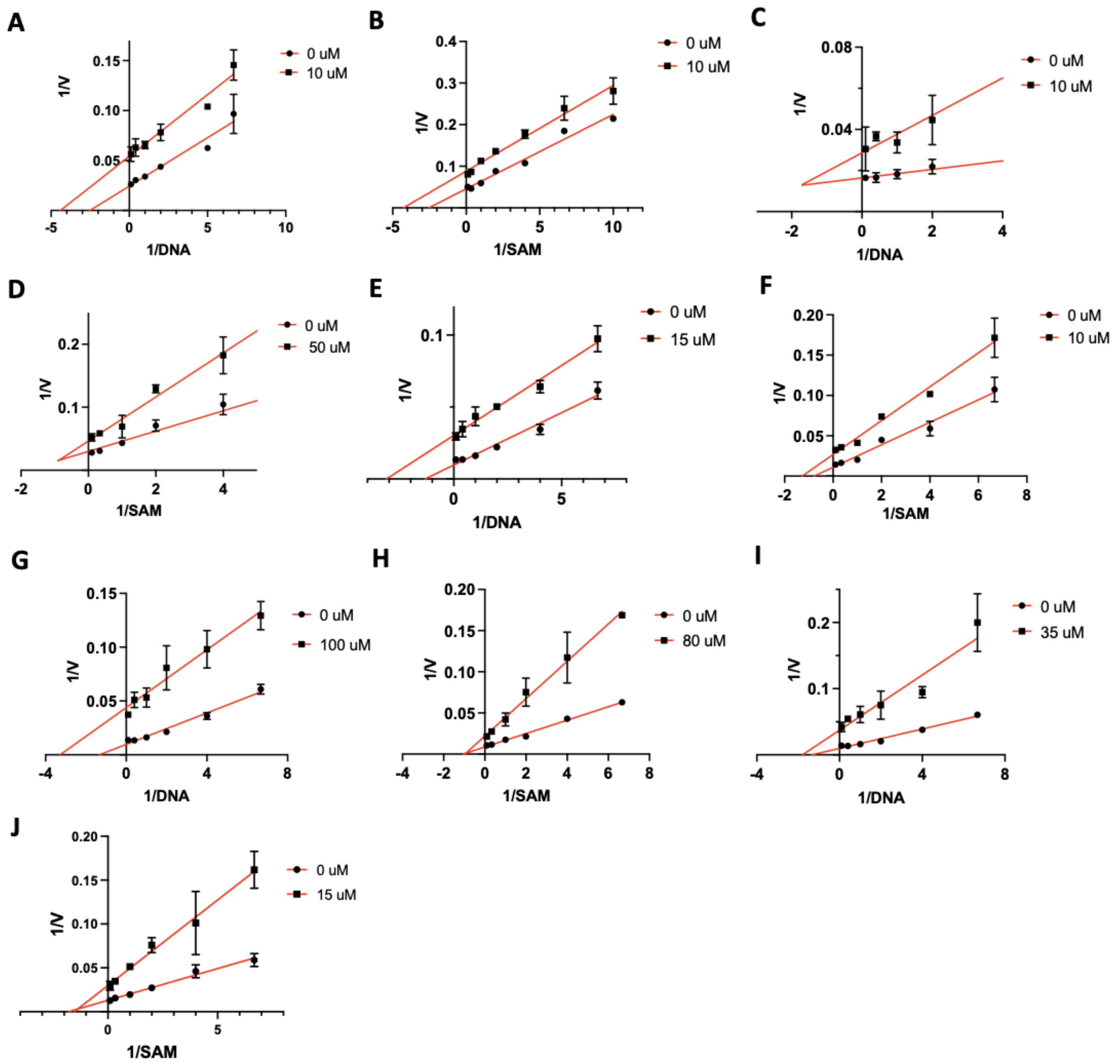

Figure S2: Double reciprocal plots for **1a** (**A, B**), **3** (**C, D**), **4** (**E, F**), **5** (**G, H**), and **10** (**I, J**) with DNA and SAM. The data points represent the mean and standard deviation of three independent reactions. Reactions were done at initial velocity conditions using 150 nM DNMT3A, and varied concentrations of one substrate (0.15–10 uM) while the other was saturated at 10 uM. Linear fits do not converge with at the y-intercept and do not have the same slope, inconsistent with competitive inhibition and uncompetitive inhibition respectively.

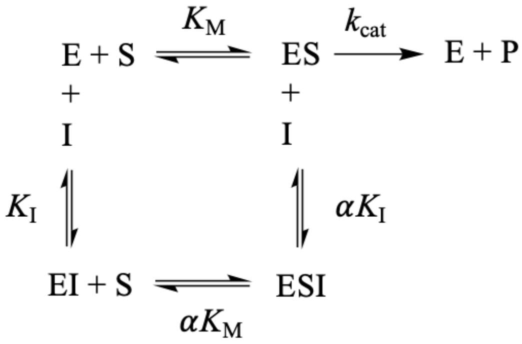

Figure S3: Kinetic scheme of mixed-type inhibition model used for derivation of $\boldsymbol{\alpha}$. The $\boldsymbol{\alpha}$ coefficient is a measure of the inhibitor's preference for E and ES kinetic species.

Table S1: Determination of best fit inhibition models for **1a**, **3**, **4**, **5**, and **10** by R^2 comparison and F-test. All curves best fit a mixed-type inhibition model with a p-value < 0.0001.

**Substrate**	**DNA**					**SAM**				
**Compound**	**1a**	**3**	**4**	**5**	**10**	**1a**	**3**	**4**	**5**	**10**
**Competitive**	0.70	0.69	0.83	0.92	0.89	0.81	0.84	0.84	0.94	0.89
**Mixed**	0.96	0.90	0.98	0.97	0.97	0.96	0.96	0.96	0.99	0.97

Table S2: Extracted α coefficient values from *Prism* using the mixed-type inhibition model. Most of the inhibitors have $\alpha < 1$, where the inhibitor prefers the enzyme-substrate complex. Data from compounds **3** and **10** with varied DNA and SAM respectively have $\alpha > 1$, however their error is high.

**Compound ID**	**DNA**	**SAM**
1a	0.2 ± 0.2	0.6 ± 0.3
3	3 ± 2	0.7 ± 0.4
4	0.7 ± 0.3	0.5 ± 0.3
5	0.6 ± 0.6	0.7 ± 0.7
10	0.9 ± 0.5	2 ± 0.7

4. General Methods of Chemistry

In reactions where water was not present as a solvent, reagent, or byproduct, the glassware was flame dried, and the reactions were carried out under an inert atmosphere of nitrogen. Reactions were monitored by analytical thin-layer chromatography on EMD silica gel 60 F254 plates; visualization was effected by ultraviolet light (254 nm), *p*-anisaldehyde or potassium permanganate stains. Column chromatography was performed using silica gel (200-300 mesh).

Solvents were removed using a rotary evaporator. If the product was non-volatile, trace solvents were removed at a reduced pressure of approximately 2 mmHg.

All purchased chemicals were used without purification unless otherwise stated. Dichloromethane was distilled from CaH_2. Diethyl ether, tetrahydrofuran, and toluene were distilled from sodium and benzophenone. Deuterated chloroform was stored over anhydrous potassium carbonate and 4Å molecular sieves before use.

^{1}H-NMR spectra were recorded at Varian or Bruker, 500 or 600 MHz instruments with the solvent resonance of $CDCl_3$ (7.26 ppm), CD_3OD (3.31 ppm), $(CD_3)_2SO$ (2.50 ppm). ^{13}C-NMR spectra were recorded at 500 or 600 MHz instruments with a solvent resonance of $CDCl_3$ (77.0 ppm) and CD_3OD (49.0 ppm). High resolution mass spectra (HRMS) were obtained by electrospray ionization/time-of-flight experiments.

Safety Statement

No unexpected or unusually high safety hazards were encountered during synthetic experimentation.

5. Chemical Synthesis and Compound Characterization

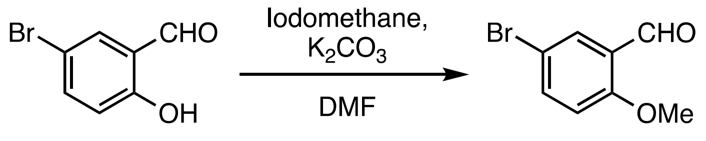

1

5-bromo-2-methoxybenzaldehyde (1)

Synthesis is previously reported.[1]

^{1}HNMR: (600 MHz, $CDCl_3$): δ 10.39 (s, 1H), 7.93 (d, *J* = 2.6 Hz, 1H), 7.64 (dd, *J* = 8.9, 2.6 Hz, 1H), 6.90 (d, 8.9 Hz, 1H), 3.93 (s, 3H). Our characterization data matches with prior literature data.[1]

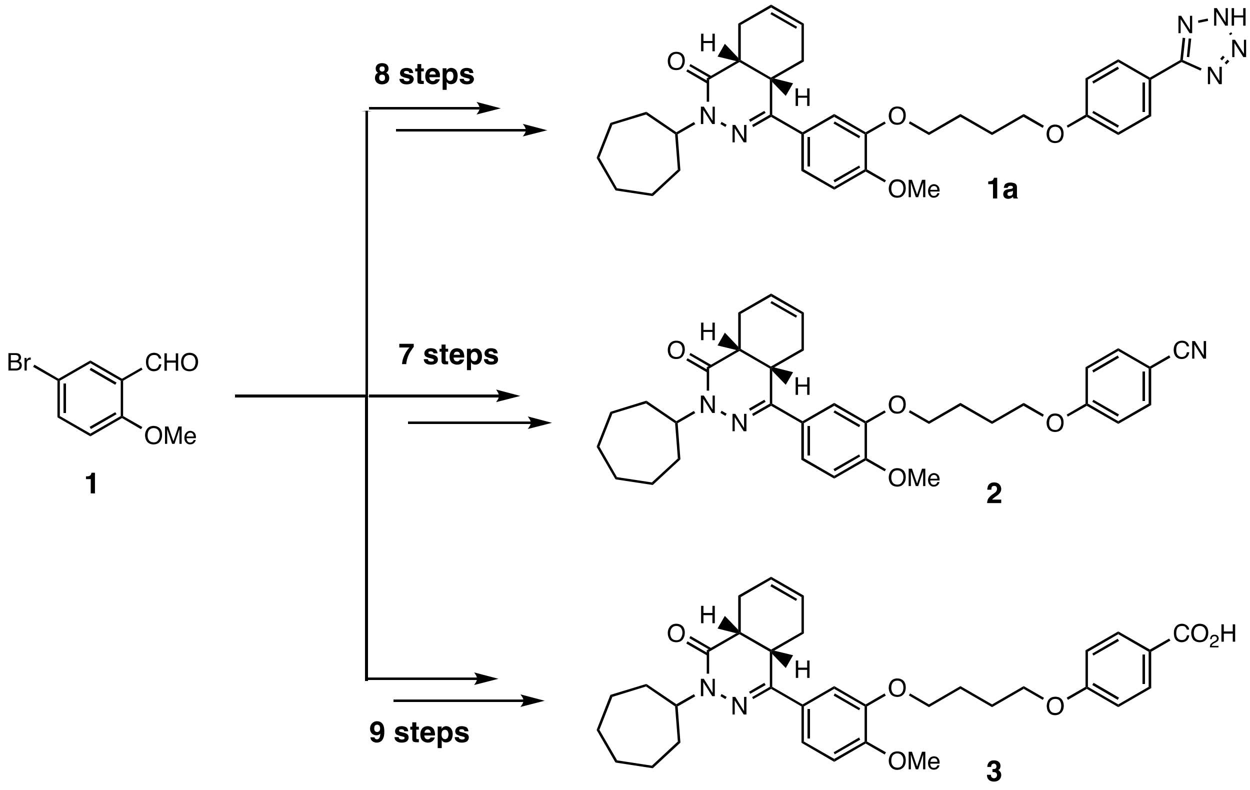

(4aS,8aR)-4-(3-(4-(4-(2H-tetrazol-5-yl)phenoxy)butoxy)-4-methoxyphenyl)-2-cycloheptyl-4a,5,8,8a-tetrahydrophthalazin-1(2H)-one (1a)

Prepared according to prior synthesis from compound **(1)** in 8 steps.[2]

4-(4-(5-((4a*R*,8a*S*)-3-cycloheptyl-4-oxo-3,4,4a,5,8,8a-hexahydrophthalazin-1-yl)-2-methoxyphenoxy)butoxy)benzonitrile (2)

Prepared according to prior synthesis from **(1)** in 7 steps.[2]

4-(4-(5-((4a*R*,8a*S*)-3-cycloheptyl-4-oxo-3,4,4a,5,8,8a-hexahydrophthalazin-1-yl)-2-methoxyphenoxy)butoxy)benzoic acid (3)

Prepared according to prior synthesis from **(1)** in 9 steps.[2]

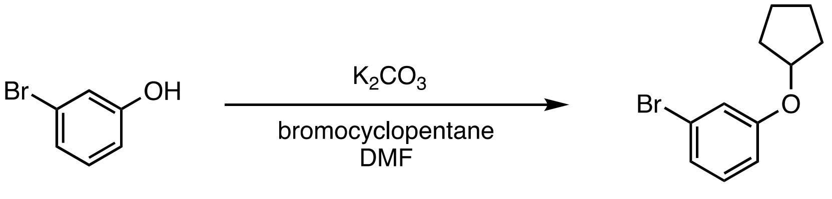

4a

1-bromo-3-(cyclopentyloxy)benzene (4a)

To a flame dried reaction flask, under N_2, was added K_2CO_3 (8.0 g, 57.8 mmol, 2.0 equiv.), 3-bromophenol (3.1 mL, 28.9 mmol, 1.0 equiv.), bromocyclopentane (5.31 mL, 49.9 mmol, 1.73 equiv.), and anhydrous DMF (40 mL). The reaction mixture was heated to 65°C and was allowed to stir for 12 h. The solution was then quenched with H_2O (20 mL) and extracted with Et_2O (3 x 20 mL). The organic layer was the washed with H_2O (3 x 20 mL), dried with brine, dried over $MgSO_4$, and concentrated. The crude product was purified by column chromatography (SiO_2: eluent: hexanes/ethyl acetate = 5:1) to afford the title compound. Yellow oil (7.1 g, >99% isolated yield).

^{1}HNMR: (500 MHz, $CDCl_3$): δ 7.13-7.09 (m, 1H), 7.04-7.02 (m, 2H), 6.81-6.78 (m, 1H), 4.74-4.71 (m, 1H), 1.93-1.60 (m, 8H).

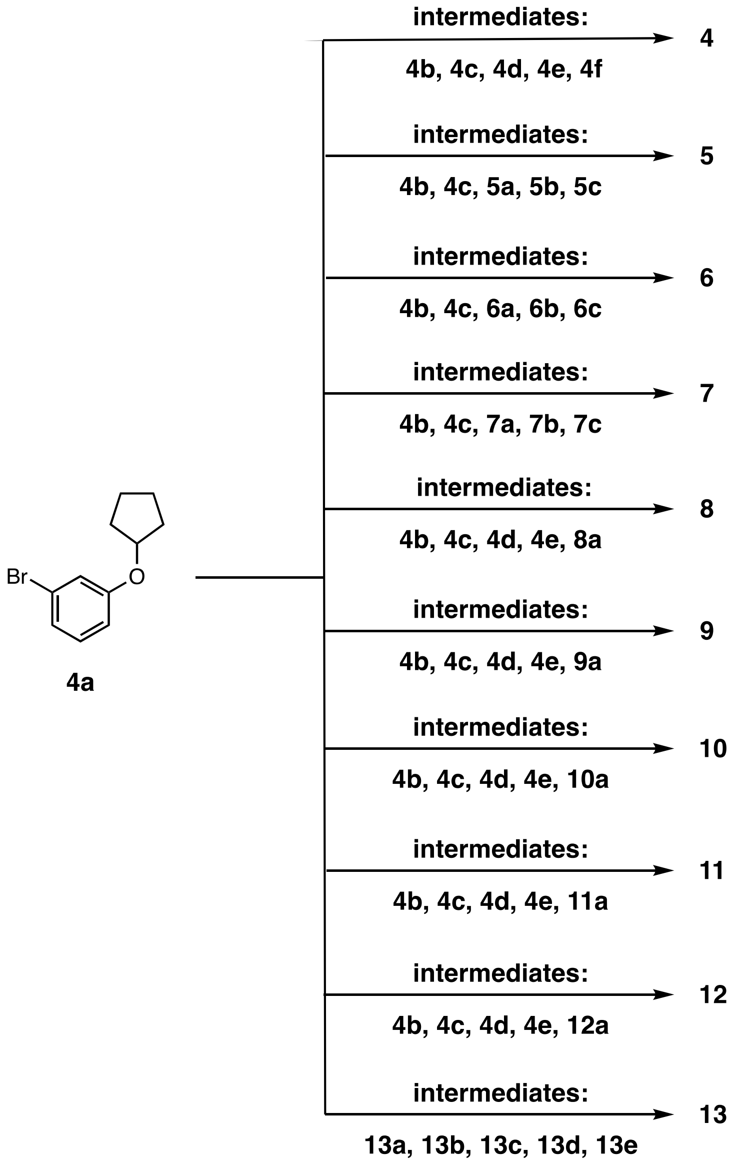

Scheme S1: Synthetic outline of compounds **4-13** from compound **4a** including all intermediates involved for each respective final product.

General Procedure A (Grignard Reaction)

To a flame dried reaction flask charged with a suspension of Mg turnings (1.2 equiv.) in anhydrous THF (0.3 M), under N_2, was added compound **(4a)** (1.0 equiv.) dissolved in anhydrous THF (1.0 M). Reaction mixture was refluxed for 4 hours. After allowing the reaction mixture to cool to room temperature, the mixture was added dropwise to a solution of either (3a*R*,7a*S*)-3a,4,7,7a-tetrahydroisobenzofuran-1,3-dione (1.04 equiv.) or isobenzofuran-1,3-dione (1.04 equiv.) in anhydrous THF (0.5 M) at 0°C and stirred for 30 minutes. The reaction was slowly warmed to room temperature and was allowed to stir for an additional 16 hours. The solution was quenched with saturated NH_4Cl (30 mL) and extracted with Et_2O (3 x 20 mL). The combined organic layers were extracted with 1N KOH (30 mL), and the basic solution was acidified with HCl. The acidic solution was extracted with Et_2O (3 x 20 mL), dried with brine, dried over anhydrous $MgSO_4$, and concentrated. The crude product was purified by column chromatography (SiO_2: eluent: hexanes/ethyl acetate = 5:1→1:1) to afford pure products.

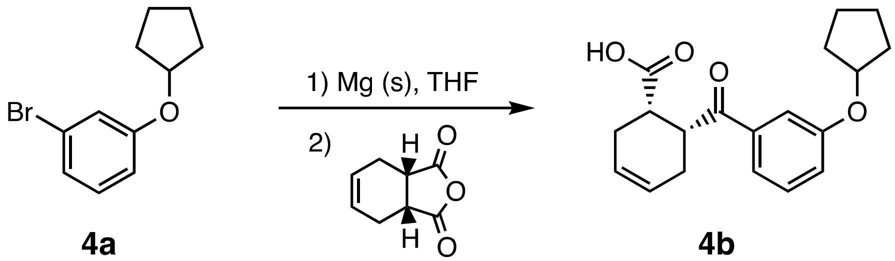

4a **4b**

(1*S*,6*R*)-6-(3-(cyclopentyloxy)benzoyl)cyclohex-3-ene-1-carboxylic acid (4b)

Prepared according to the **general procedure A** using compound **(4a)** and (3a*R*,7a*S*)-3a,4,7,7a-tetrahydroisobenzofuran-1,3-dione. White solid (5.89 g, 61.2% isolated yield).

^{1}HNMR: (500 MHz, $CDCl_3$): δ 7.41-7.31 (m, 3H), 7.05 (dd, *J* = 8.1, 1.6 Hz, 1H), 5.78-5.60 (m, 2H), 4.79 (m, 1H), 3.93 (m, 1H), 3.02 (m, 1H), 2.83-2.78 (m, 1H), 2.52-2.43 (m, 3H), 1.96-1.58 (m, 8H). $\mathbf{R_f}$ = 0.33 (hexanes/ethyl acetate = 1:1).

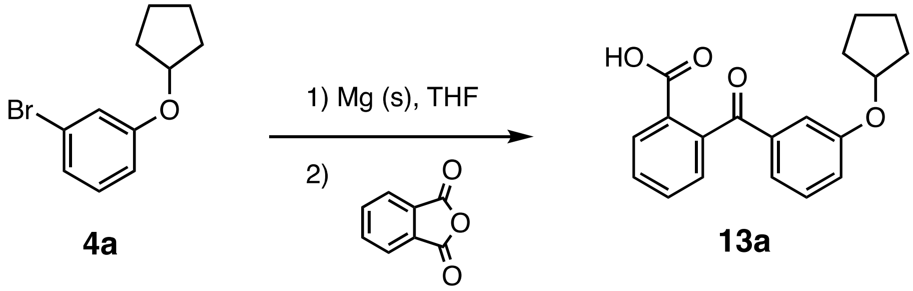

4a **13a**

2-(3-(cyclopentyloxy)benzoyl)benzoic acid (13a)

Prepared according to the **general procedure A** using compound **(4a)** and isobenzofuran-1,3-dione. White solid (5.96 g, 73.9% isolated yield).

^{1}HNMR: (500 MHz, $CDCl_3$): δ 8.07 (d, J = 8.0 Hz, 1H), 7.67-7.54 (m, 2H), 7.38-7.24 (m, 3H), 7.15-7.04 (m, 2H), 4.80-4.78 (m, 1H), 1.93-1.60 (m, 8H). **R_f** = 0.44 (hexanes/ethyl acetate = 1:1).

General Procedure B (Hydrazine)

To a reaction flask charged with compounds **(4b)** or **(13a)** (1.0 equiv.) in EtOH (0.19 M), under N_2, was added hydrazine monohydrate (3.0 equiv.) and the mixture was allowed to reflux for 4 hours. After the mixture was cooled to room temperature, it was concentrated and dissolved in ethyl acetate (40 mL). The organic layer was washed with H_2O (20 mL), 1N HCl (20 mL), and saturated $NaHCO_3$ (20 mL). The organic solution was then dried with brine, dried over anhydrous $MgSO_4$, and concentrated. The crude product was purified by column chromatography (SiO_2: eluent: hexanes/ethyl acetate = 8:1→2:1) to afford pure products.

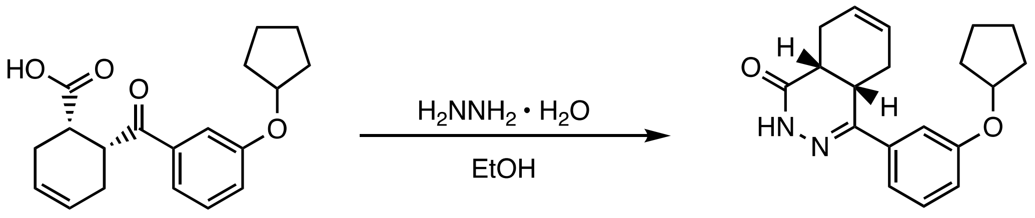

4b 4c

(4a*S*,8a*R*)-4-(3-(cyclopentyloxy)phenyl)-4a,5,8,8a-tetrahydrophthalazin-1(2*H*)-one (4c)

Prepared according to the **general procedure B** using compound **(4b)**. White solid (3.14 g, 54.1% isolated yield).

^{1}HNMR: (600 MHz, $CDCl_3$): δ 8.62 (s, 1H), 7.32-7.30 (m, 3H), 6.93-6.91 (m, 1H), 5.79-5.69 (m, 2H), 4.83-4.80 (m, 1H), 3.40-3.37 (m, 1H), 2.92-2.80 (m, 2H), 2.99-2.97 (m, 1H), 2.84 (t, J = 5.6 Hz), 2.25-1.77 (m, 10H). **R_f** = 0.57 (hexanes/ethyl acetate = 1:1).

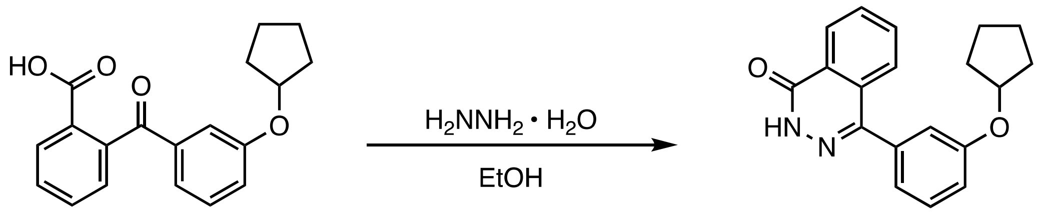

13a 13b

4-(3-(cyclopentyloxy)phenyl)phthalazine-1(2*H*)-one (13b)

Prepared according to the **general procedure B** using compound **(13a)**. Crude product crystallized out of reaction mixture upon cooling to room temperature, therefore it was filtered off and washed with water and EtOH to yield the pure product without any further purification. White solid (4.87 g, 82.7% isolated yield).

^{1}HNMR: (600 MHz, $CDCl_3$): δ 10.60 (m, 1H), 8.53-8.51 (m, 1H), 7.81-7.78 (m, 3H), 7.42-7.39 (m, 1H), 7.12-7.01 (m, 3H), 4.83-4.81 (m, 1H), 1.94-1.60 (m, 8H). **R_f** = 0.30 (hexanes/ethyl acetate = 1:1).

General Procedure C (Amide Alkylation)

To a flame dried reaction flask, under N_2, charged with NaH (60 % in oil, 1.1 equiv.) was slowly added compounds **(4c)** or **(13b)** (1.0 equiv.) dissolved in anhydrous DMF (0.28 M) and the mixture was allowed to stir for 1.5 hours at room temperature. Organohalide (1.1 equiv.) and KI (60 mol %) were then added to the reaction mixture and it was allowed to stir for 16 hours at room temperature. The solution was quenched with H_2O (10 mL) and extracted with ethyl acetate (3 x 30 mL). The combined organic layers were washed with H_2O (3 x 20 mL), dried with brine, dried over anhydrous $MgSO_4$, and concentrated. The crude product was purified by column chromatography (SiO_2: eluent: hexanes/ethyl acetate = 5:1) to afford pure products.

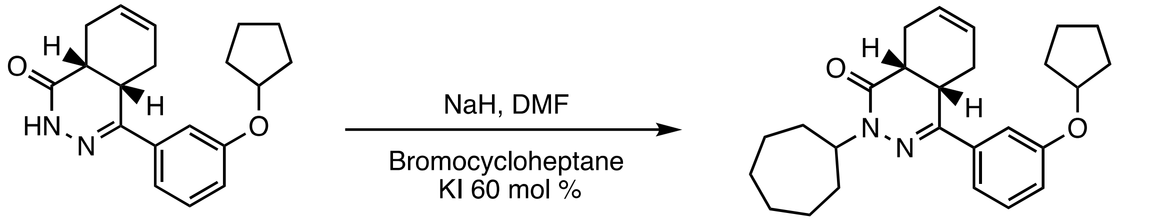

4c 4d

(4a*S*,8a*R*)-2-cycloheptyl-4-(3-(cyclopentyloxy)phenyl)-4a,5,8,8a-tetrahydrophthalazin-1(2*H*)-one (4d)

Prepared according to the **general procedure C** using compound **(4c)** and bromocycloheptane. White solid (700 mg, 53.5% isolated yield).

^{1}HNMR: (500 MHz, $CDCl_3$): δ 7.36-7.29 (m, 3H), 6.92-6.90 (m, 1H), 5.77-5.67 (m, 2H), 4.84-4.79 (m, 2H), 3.31-3.27 (m, 1H), 3.01-2.98 (m, 1H), 2.72 (t, J = 5.9 Hz, 1H), 2.20-1.51 (m, 23H) **R_f** = 0.49 (hexanes/ethyl acetate = 3:1).

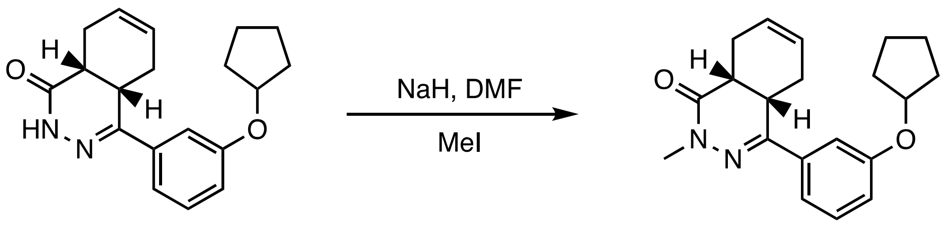

4c **5a**

(4a*S*,8a*R*)-4-(3-(cyclopentyloxy)phenyl)-2-methyl-4a,5,8,8a-tetrahydrophthalazin-1(2*H*)-one (5a)

Prepared according to the **general procedure C** using compound **(4c)** and iodomethane. No addition of KI (60% mol) was necessary. Yellow oil (238 mg, 75.6% isolated yield).

^{1}HNMR: (600 MHz, $CDCl_3$): δ 7.30-7.25 (m, 3H), 6.89-6.87 (m, 1H), 5.75-5.63 (m, 2H), 4.80-4.77 (m, 1H), 3.43 (s, 3H), 3.31-3.27 (m, 1H), 2.98-2.94 (m, 1H), 2.74 (t, *J* = 5.8 Hz, 1H), 2.21-1.60 (m, 11H). $\mathbf{R_f}$ = 0.49 (hexanes/ethyl acetate = 3:1).

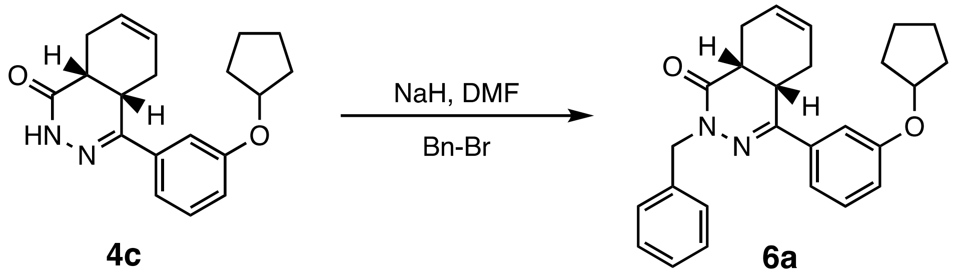

4c **6a**

(4a*S*,8a*R*)-2-benzyl-4-(3-(cyclopentyloxy)phenyl)-4a,5,8,8a-tetrahydrophthalazin-1(2*H*)-one (6a)

Prepared according to the **general procedure C** using compound **(4c)** and benzyl-bromide. Yellow oil (246 mg, 95.8% isolated yield).

^{1}HNMR: (600 MHz, $CDCl_3$): δ 7.38-7.21 (m, 8H), 6.88 (dt, *J* = 7.6, 1.7 Hz, 1H), 5.75-5.61 (m, 2H), 5.12 (d, *J* = 14.3 Hz, 1H), 4.90 (d, *J* = 14.3 Hz, 1H), 4.78-4.75 (m, 1H), 3.32-3.28 (m, 1H), 3.00-2.98 (m, 1H), 2.79 (t, *J* = 6.1 Hz, 1H), 2.19-1.60 (m, 11H). $\mathbf{R_f}$ = 0.54 (hexanes/ethyl acetate = 3:1).

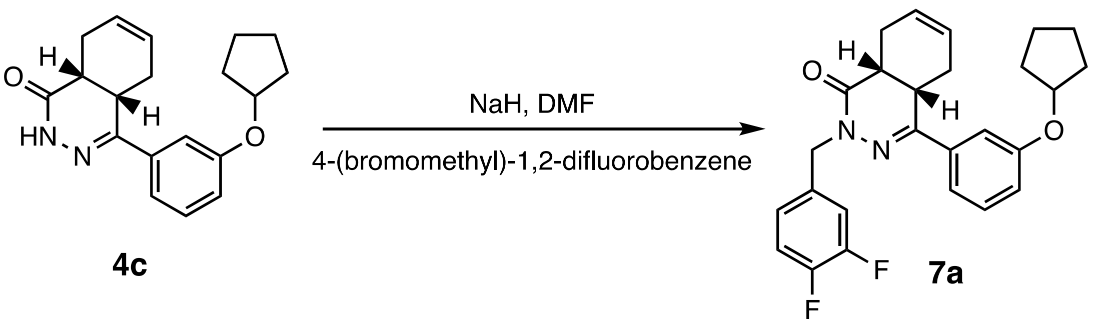

(4a*S*,8a*R*)-4-(3-(cyclopentyloxy)phenyl)-2-(3,4-difluorobenzyl)-4a,5,8,8a-tetrahydrophthalazin-1(2*H*)-one (7a)

Prepared according to the **general procedure C** using compound **(4c)** and 4-(bromomethyl)-1,2-difluorobenzene. Yellow oil (351 mg, 82.9% isolated yield).

^{1}HNMR: (600 MHz, $CDCl_3$): δ 7.30-7.04 (m, 6H), 6.92-6.90 (m, 1H), 5.76-5.63 (m, 2H), 5.02 (d, *J* = 14.3 Hz, 1H), 4.87 (d, *J* = 14.3 Hz, 1H), 4.80-4.77 (m, 1H), 3.34-3.31 (m, 1H), 2.99-2.96 (m, 1H), 2.79 (t, *J* = 5.7 Hz, 1H), 2.22-1.61 (m, 11H). **R_f** = 0.50 (hexanes/ethyl acetate = 3:1).

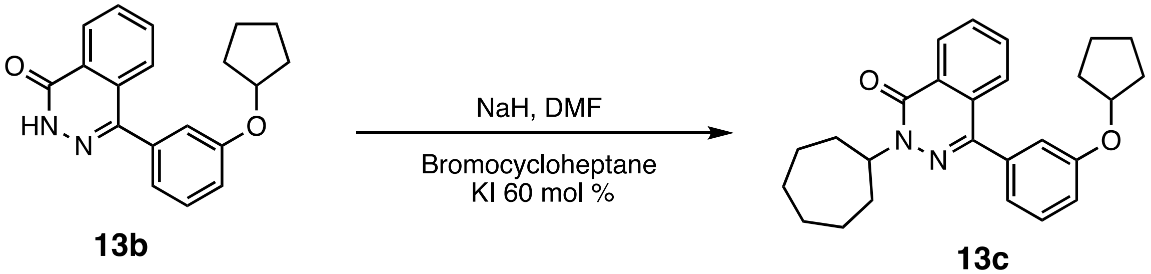

2-cycloheptyl-4-(3-(cyclopentyloxy)phenyl)phthalazine-1(2*H*)-one (13c)

Prepared according to the **general procedure C** using compound **(13b)** and bromocycloheptane. White solid. (623 mg, 88.4% isolated yield).

^{1}HNMR: (500 MHz, $CDCl_3$): δ 8.52-8.50 (m, 1H), 7.79-7.68 (m, 3H), 7.39-7.36 (m, 1H), 7.14-6.97 (m, 3H), 5.26-5.21 (m, 1H), 4.82-4.78 (m, 1H), 2.09-1.53 (m, 20H). **R_f** = 0.58 (hexanes/ethyl acetate = 3:1).

General Procedure D (Ether Deprotection)

Using a dean stark setup, a reaction flask was charged with compounds **(4d), (5a), (6a), (7a), or (13c)** (1 equiv.), *p*-toluene sulfonic acid monohydrate (1.2 equiv.), and toluene (0.15 M). The reaction mixture was refluxed for 4 hours and was frequently monitored by thin layer chromatography (hexanes/ethyl acetate = 1:1). The mixture was concentrated and dissolved in ethyl acetate (20 mL). The organic layer was washed with saturated $NaHCO_3$ (20 mL), dried with

brine, dried over anhydrous $MgSO_4$, and concentrated. The crude product was purified by column chromatography (SiO_2: eluent: hexanes/ethyl acetate = 5:1→2:1) to afford pure products.

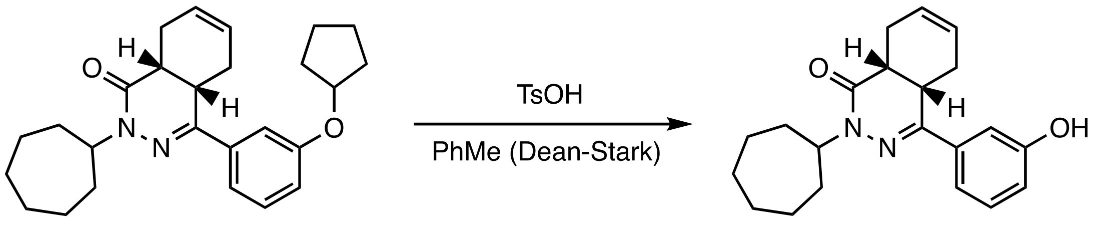

4d 4e

(4a*S*,8a*R*)-2-cycloheptyl-4-(3-hydroxyphenyl)-4a,5,8,8a-tetrahydrophthalazin-1(2*H*)-one (4e)

Prepared according to the **general procedure D** using compound **(4d)**. White solid (195 mg, 37.6% isolated yield).

^{1}HNMR: (500 MHz, $CDCl_3$): δ 7.36-7.25 (m, 3H), 6.93-6.91 (m, 1H), 5.77-5.63 (m, 2H), 4.82-4.76 (m, 1H), 3.30-3.26 (m, 1H), 3.00-2.96 (m, 1H), 2.73 (t, J = 5.2 Hz, 1H), 2.22-1.46 (m, 15H). Phenol peak is unobserved in the NMR spectra. **R_f** = 0.34 (hexanes/ethyl acetate = 3:1).

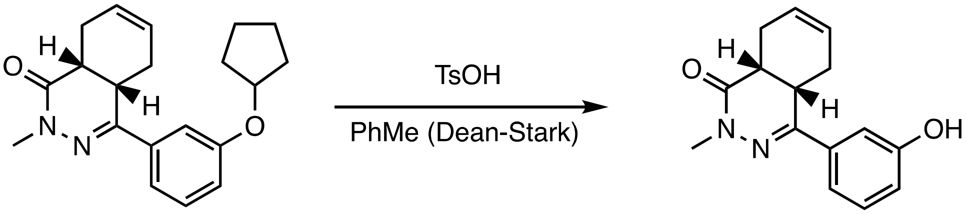

5a 5b

(4a*S*,8a*R*)-4-(3-hydroxyphenyl)-2-methyl-4a,5,8,8a-tetrahydrophthalazin-1(2*H*)-one (5b)

Prepared according to the **general procedure D** using compound **(5a)**. White solid (85.1 mg, 45.5% isolated yield).

^{1}HNMR: (600 MHz, $CDCl_3$): δ 7.33-7.22 (m, 3H), 6.93-6.91 (m, 1H), 5.75-5.62 (m, 2H), 3.45 (s, 3H), 3.32-3.28 (m, 1H), 2.98-2.94 (m, 1H), 2.78 (t, J = 5.7 Hz, 1H), 2.21-2.03 (m, 3H). Phenol peak is unobserved in the NMR spectra. **R_f** = 0.32 (hexanes/ethyl acetate = 3:1).

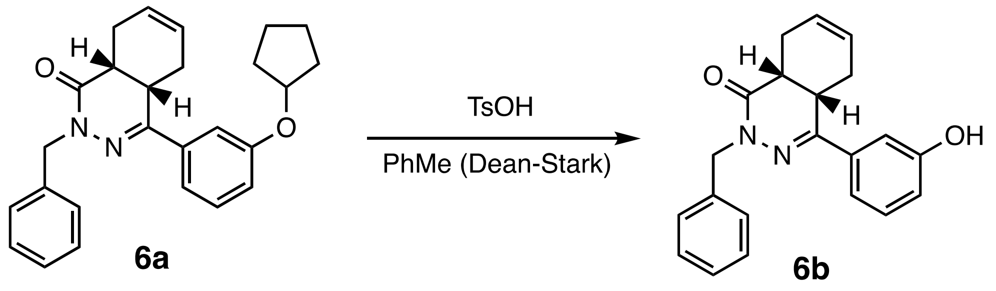

(4a*S*,8a*R*)-2-benzyl-4-(3-hydroxyphenyl)-4a,5,8,8a-tetrahydrophthalazin-1(2*H*)-one (6b)

Prepared according to the **general procedure D** using compound **(6a)**. Yellow solid (120 mg, 59.2% isolated yield).

^{1}HNMR: (600 MHz, $CDCl_3$): δ 7.27-7.13 (m, 8H), 6.80-6.78 (m, 1H), 5.65-5.51 (m, 2H), 5.04 (d, *J* = 14.3 Hz, 1H), 4.81 (d, *J* = 14.3 Hz, 1H), 3.22-3.18 (m, 1H), 2.90-2.86 (m, 1H), 2.71 (t, *J* = 6.0 Hz, 1H), 2.12-1.80 (m, 3H). Phenol peak is unobserved in the NMR spectra. **R_f** = 0.63 (hexanes/ethyl acetate = 1:1).

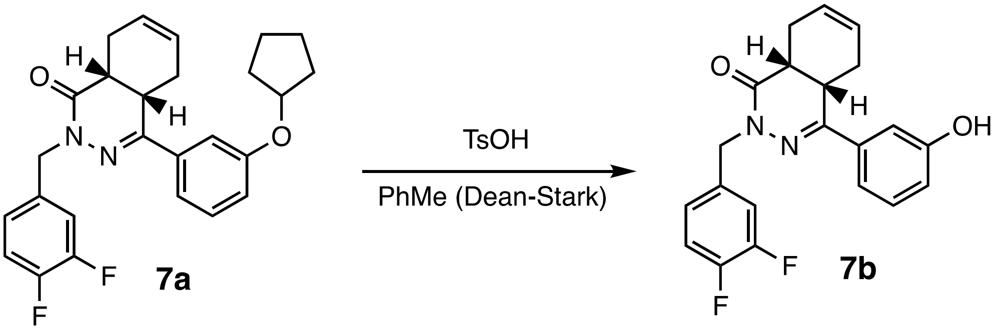

(4a*S*,8a*R*)-2-(3,4-difluorobenzyl)-4-(3-hydroxyphenyl)-4a,5,8,8a-tetrahydrophthalazin-1(2*H*)-one (7b)

Prepared according to the **general procedure D** using compound **(7a)**. Yellow solid (114 mg, 59.5% isolated yield).

^{1}HNMR: (600 MHz, $CDCl_3$): δ 7.30-7.01 (m, 6H), 6.90-6.88 (m, 1H), 5.76-5.61 (m, 2H), 5.01 (d, *J* = 14.4 Hz, 1H), 4.85 (d, *J* = 14.4 Hz, 1H), 3.33-3.29 (m, 1H), 2.98-2.94 (m, 1H), 2.81 (t, *J* = 5.6 Hz, 1H), 2.22-1.87 (m, 3H). Phenol peak is unobserved in the NMR spectra. **R_f** = 0.51 (hexanes/ethyl acetate = 1:1).

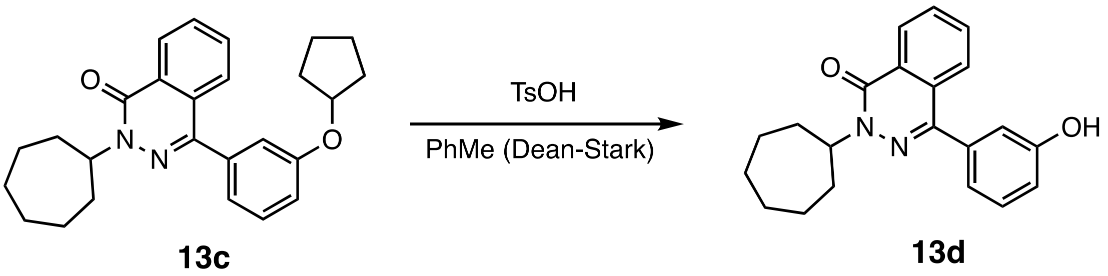

2-cycloheptyl-4-(3-hydroxyphenyl)phthalazine-1(2*H*)-one (13d)

Prepared according to the **general procedure D** using compound **(13c)**. White solid (195 mg, 37.6% isolated yield).

^{1}HNMR: (600 MHz, $CDCl_3$): δ 8.53-8.51 (m, 1H), 7.78-7.69 (m, 3H), 7.40-7.37 (m, 1H), 7.15-7.14 (m, 1H), 7.07-7.00 (m, 2H), 5.24-5.20 (m, 1H), 2.05-1.54 (m, 12H). Phenol peak is unobserved in the NMR spectra. **R$_f$** = 0.19 (hexanes/ethyl acetate = 3:1).

General Procedure E (Linker Formation)

To a flame dried reaction flask charged with NaH (60 % in oil, 1.3-1.5 equiv.) in anhydrous DMF (0.1M), under N_2, was added compounds **(4e), (5b), (6b), (7b), (13d)** or **phenol** (1.0 equiv.) and the mixture was allowed to stir for 1.5 hours at room temperature. Compounds **(L3-L6)** (1.3-1.5 equiv.) were then added to the reaction mixture, and it was allowed to stir for 16 hours at room temperature. The solution was quenched with H_2O (10 mL) and extracted with Et_2O (3 x 30 mL). The combined organic layers were washed with H_2O (3 x 20 mL), dried with brine, dried over anhydrous Na_2SO_4, and concentrated. The crude product was purified by column chromatography to afford pure products.

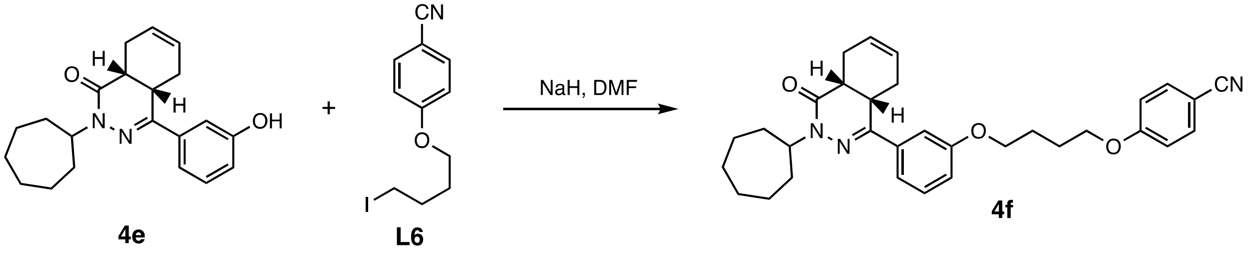

4-(4-(3-((4a*R*,8a*S*)-3-cycloheptyl-4-oxo-3,4,4a,5,8,8a-hexahydrophthalazin-1-yl)phenoxy)butoxy)benzonitrile (4f)

Prepared according to the **general procedure E** using compound **(4e)** and **(L6)**. White solid (55 mg, 40.4% isolated yield).

^{1}HNMR: (600 MHz, $CDCl_3$): δ 7.57 (d, *J* = 8.9 Hz, 2H), 7.38-7.31 (m, 3H), 6.94 (d, *J* = 8.9 Hz, 2H), 6.92-6.91 (m, 1H), 5.79-5.65 (m, 2H), 4.83-4.79 (m, 1H), 4.11-4.08 (m, 4H), 3.31-3.27 (m,

1H), 3.01-2.97 (m, 1H), 2.72 (t, *J* = 6.2 Hz, 1H), 2.21-1.48 (m, 19H). **R_f** = 0.28 (hexanes/ethyl acetate = 3:1).

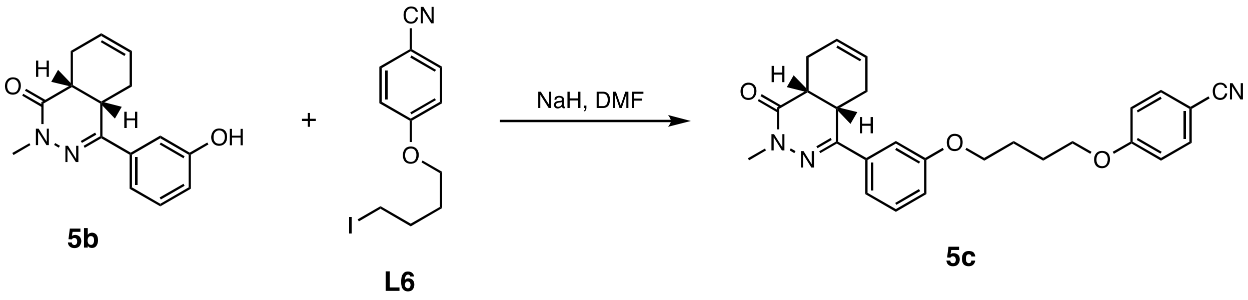

4-(4-(3-((4a*R*,8a*S*)-3-methyl-4-oxo-3,4,4a,5,8,8a-hexahydrophthalazin-1-yl)phenoxy)butoxy)benzonitrile (5c)

Prepared according to the **general procedure E** using compound **(5b)** and **(L6)**. White solid (88 mg, 87.6% isolated yield).

^{1}HNMR: (600 MHz, $CDCl_3$): δ 7.56 (d, *J* = 7.2 Hz, 2H), 7.35-7.29 (m, 3H), 6.93-6.92 (m, 3H), 5.77-5.65 (m, 2H), 4.09-4.07 (m, 4H), 3.45 (s, 3H), 3.33-3.30 (m, 1H), 3.00-2.96 (m, 1H), 2.77 (t, *J* = 6.1 Hz, 1H), 2.23-1.97 (m, 7H). **R_f** = 0.24 (hexanes/ethyl acetate = 3:1).

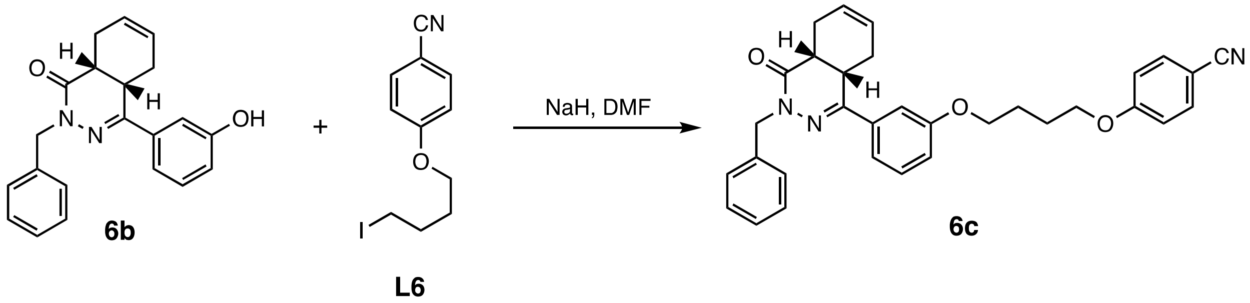

4-(4-(3-((4a*R*,8a*S*)-3-benzyl-4-oxo-3,4,4a,5,8,8a-hexahydrophthalazin-1-yl)phenoxy)butoxy)benzonitrile (6c)

Prepared according to the **general procedure E** using compound **(6b)** and **(L6)**. White solid (47.1 mg, 58.2% isolated yield).

^{1}HNMR: (600 MHz, $CDCl_3$): δ 7.53 (d, *J* = 8.9 Hz, 2H), 7.36-7.19 (m, 8H), 6.91-6.88 (m, 3H), 5.75-5.61 (m, 2H), 5.12 (d, *J* = 14.5 Hz, 1H), 4.88 (d, *J* = 14.5 Hz, 1H), 4.07-4.03 (m, 4H), 3.31-3.27 (m, 1H), 3.00-2.96 (m, 1H), 2.79 (t, *J* = 6.1 Hz, 1H), 2.20-1.94 (m, 7H). **R_f** = 0.29 (hexanes/ethyl acetate = 3:1).

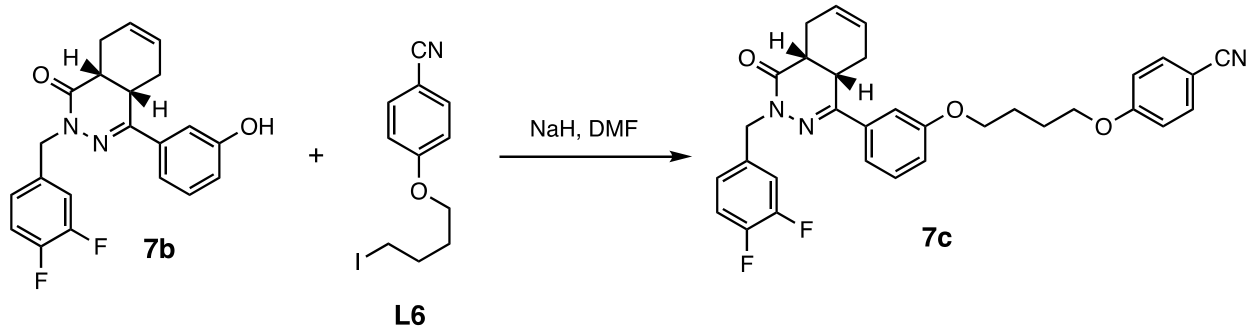

4-(4-(3-((4a*R*,8a*S*)-3-(3,4-difluorobenzyl)-4-oxo-3,4,4a,5,8,8a-hexahydrophthalazin-1-yl)phenoxy)butoxy)benzonitrile (7c)

Prepared according to the **general procedure E** using compound **(7b)** and **(L6)**. White solid (119 mg, 78.5% isolated yield).

^{1}HNMR: (600 MHz, $CDCl_3$): δ 7.55 (d, *J* = 8.9 Hz, 2H) 7.31-7.03 (m, 6H), 6.93-6.91 (m, 3H), 5.77-5.62 (m, 2H), 5.02 (d, *J* = 14.5 Hz, 1H), 4.86 (d, *J* = 14.5 Hz, 1H), 4.09-4.05 (m, 4H), 3.35-3.30 (m, 1H), 2.99-2.95 (m, 1H), 2.80 (t, *J* = 5.8 Hz, 1H), 2.22-1.88 (m, 7H). $\mathbf{R_f}$ = 0.17 (hexanes/ethyl acetate = 3:1).

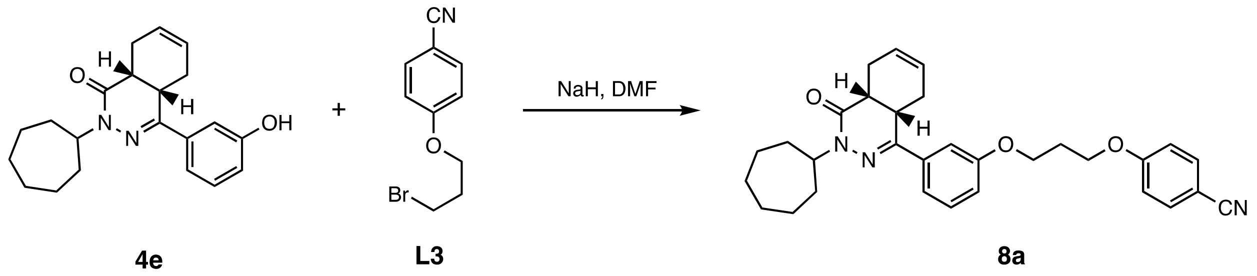

4-(3-(3-((4a*R*,8a*S*)-3-cycloheptyl-4-oxo-3,4,4a,5,8,8a-hexahydrophthalazin-1-yl)phenoxy)propoxy)benzonitrile (8a)

Prepared according to the **general procedure E** using compound **(4e)** and **(L3)**. White solid (22.3 mg, 68.9% isolated yield).

^{1}HNMR: (500 MHz, $CDCl_3$): δ 7.58 (d, *J* = 8.9 Hz, 2H), 7.39-7.31 (m, 3H), 6.97 (d, *J* = 8.9 Hz, 2H), 6.95-6.93 (m, 1H), 5.79-5.65 (m, 2H), 4.84-4.78 (m, 1H), 4.25-4.20 (m, 4H), 3.31-3.26 (m, 1H), 3.02-2.97 (m, 1H), 2.72 (t, *J* = 6.5 Hz, 1H), 2.32 (quint, *J* = 6.1 Hz, 2H). 2.22-1.48 (m, 15H). $\mathbf{R_f}$ = 0.31 (hexanes/ethyl acetate = 3:1).

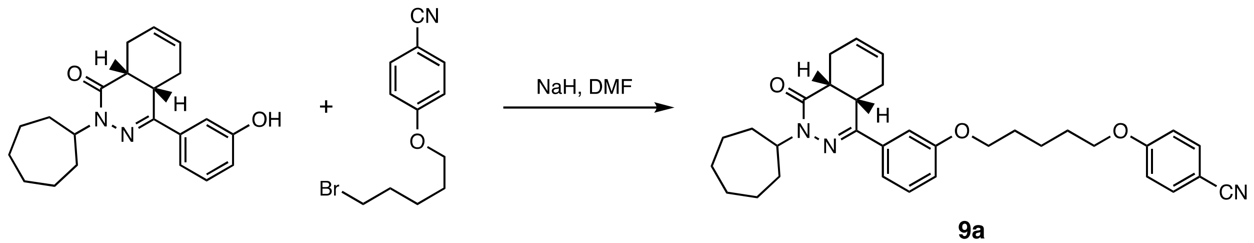

4-((5-(3-((4a*R*,8a*S*)-3-cycloheptyl-4-oxo-3,4,4a,5,8,8a-hexahydrophthalazin-1-yl)phenoxy)pentyl)oxy)benzonitrile (9a)

Prepared according to the **general procedure E** using compound **(4e)** and **(L4)**. White solid (26.4 mg, 37.5% isolated yield).

^{1}HNMR: (600 MHz, $CDCl_3$): δ 7.57 (d, *J* = 8.9 Hz, 2H), 7.38-7.31 (m, 3H), 6.94-6.92 (m, 3H), 5.79-5.65 (m, 2H), 4.84-4.79 (m, 1H), 4.05-4.03 (m, 4H), 3.31-3.27 (m, 1H), 3.02-2.98 (m, 1H), 2.72 (t, *J* = 6.3 Hz, 1H), 2.22-1.48 (m, 21H). **R_f** = 0.32 (hexanes/ethyl acetate = 3:1).

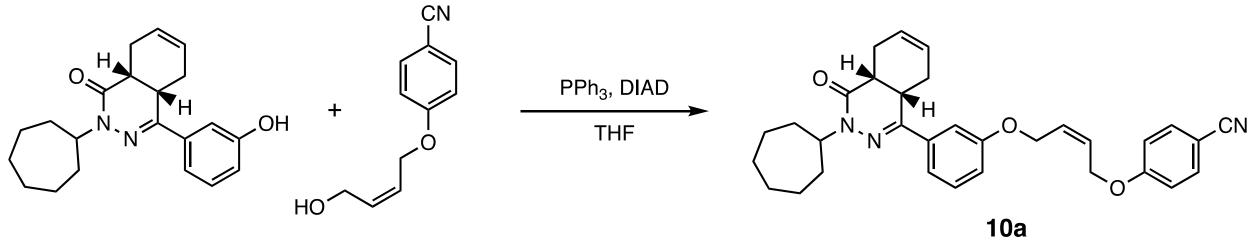

4-(((*Z*)-4-(3-((4a*R*,8a*S*)-3-cycloheptyl-4-oxo-3,4,4a,5,8,8a-hexahydrophthalazin-1-yl)phenoxy)but-2-en-1-yl)oxy)benzonitrile (10a)

To a flame dried reaction flask, under N_2, was added compound **(4e)** (41.1 mg, 0.122 mmol, 1.0 equiv.), compound **(L1)** (25.3 mg, 0.134 mmol, 1.1 equiv.), PPh_3 (38.4 mg, 0.146 mmol, 1.2 equiv.), and anhydrous THF (1 mL) and cooled to 0°C. Diisopropyl azodicarboxylate (DIAD) (0.0287 mL, 0.146 mmol, 1.2 equiv.) was added dropwise to the reaction mixture and was allowed to stir for 30 min at room temperature. The solution was concentrated, and the crude product was purified by column chromatography (SiO_2: eluent: hexanes/ethyl acetate = 8:1) to afford the title compound. White solid (19.2 mg, 30.9 % isolated yield).

^{1}HNMR: (600 MHz, $CDCl_3$): δ 7.58 (d, *J* = 8.9 Hz, 2H), 7.41-7.32 (m, 3H), 6.96-6.93 (m, 3H), 6.04-5.91 (m, 2H), 5.79-5.65 (m, 2H), 4.84-4.79 (m, 1H), 4.76 (d, *J* = 5.9 Hz, 2H), 4.72 (d, *J* = 5.9 Hz, 2H), 3.30-3.26 (m, 1H), 3.02-2.98 (m, 1H), 2.73 (t, *J* = 5.6 Hz, 1H), 2.21-1.48 (m, 15H). **R_f** = 0.23 (hexanes/ethyl acetate = 3:1).

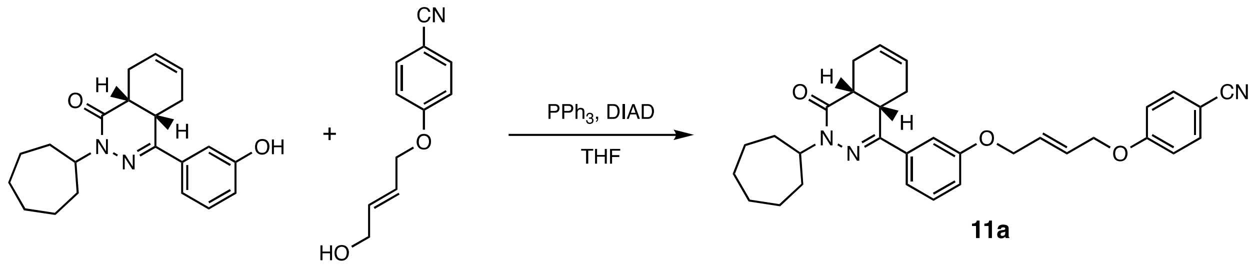

4e L2 11a

4-((€-4-(3-((4a*R*,8a*S*)-3-cycloheptyl-4-oxo-3,4,4a,5,8,8a-hexahydrophthalazin-1-yl)phenoxy)but-2-en-1-yl)oxy)benzonitrile (11a)

To a flame dried reaction flask, under N_2, was added compound **(4e)** (46.1 mg, 0.136 mmol, 1.0 equiv.), compound **(L2)** (28.3 mg, 0.15 mmol, 1.1 equiv.), PPh_3 (42.9 mg, 0.163 mmol, 1.2 equiv.), and anhydrous THF (1 mL) and cooled to 0°C. Diisopropyl azodicarboxylate (DIAD) (0.032 mL, 0.163 mmol, 1.2 equiv.) was added dropwise to the reaction mixture and was allowed to stir for 30 min at room temperature. The solution was concentrated, and the crude product was purified by column chromatography (SiO_2: eluent: hexanes/ethyl acetate = 8:1→7:1) to afford the title compound. White solid (55.1 mg, 79.5 % isolated yield).

^{1}HNMR: (600 MHz, $CDCl_3$): δ 7.57 (d, *J* = 8.8 Hz, 2H), 7.41-7.31 (m, 3H), 6.96-6.93 (m, 3H), 6.11-6.10 (m, 2H), 5.78-5.64 (m, 2H), 4.83-4.77 (m, 1H), 4.64-4.61 (m, 4H), 3.30-3.25 (m, 1H), 3.00-2.97 (m, 1H), 2.72 (t, *J* = 5.9 Hz, 1H), 2.21-1.49 (m, 15H). **R_f** = 0.33 (hexanes/ethyl acetate = 3:1).

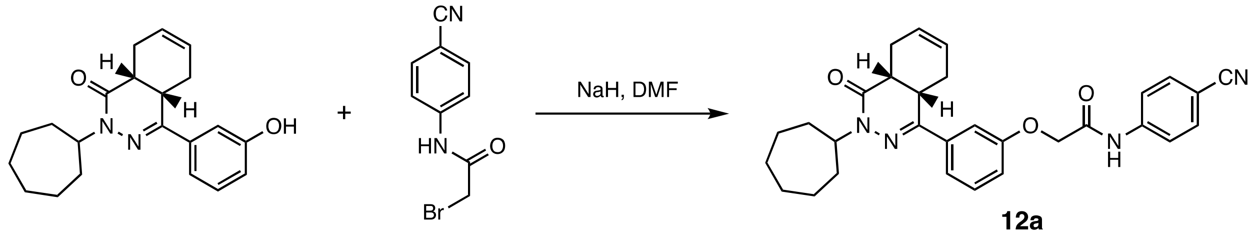

4e L5 12a

***N*-(4-cyanophenyl)-2-(3-((4a*R*,8a*S*)-3-cycloheptyl-4-oxo-3,4,4a,5,8,8a-hexahydrophthalazin-1-yl)phenoxy)acetamide (12a)**

Prepared according to the **general procedure E** using compound **(4e)** and **(L5)**. White solid (123 mg, 82.6% isolated yield).

^{1}HNMR: (500 MHz, $CDCl_3$): δ 8.55 (s, 1H), 7.77 (d, *J* = 8.9 Hz, 2H), 7.64 (d, *J* = 8.9 Hz, 2H), 7.49-7.38 (m, 3H), 7.03-7.01 (m, 1H), 5.78-5.65 (m, 2H), 4.83-4.77 (m, 1H), 4.68 (s, 2H), 3.31-3.27 (m, 1H), 3.01-2.97 (m, 1H), 2.72 (t, *J* = 6.2 Hz, 1H), 2.21-1.49 (m, 15H). **R_f** = 0.56 (hexanes/ethyl acetate = 1:1).

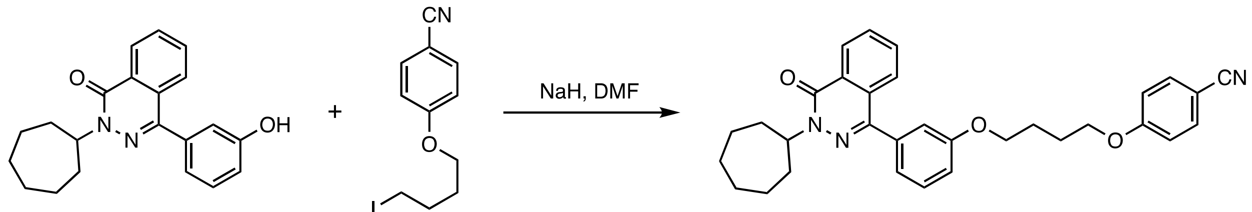

4-(4-(3-(3-cycloheptyl-4-oxo-3,4-dihydrophthalazin-1-yl)phenoxy)butoxy)benzonitrile (13e)

Prepared according to the **general procedure E** using compound **(13d)** and **(L6)**. White solid (50mg, 32.8% isolated yield).

^{1}HNMR: (500 MHz, $CDCl_3$): δ 8.54 (d, *J* = 8.0 Hz, 1H), 7.78-7.70 (m, 3H), 7.57 (d, *J* = 8.9 Hz, 2H), 7.45-7.41 (m, 1H), 7.19-7.12 (m, 2H), 7.03-7.02 (m, 1H), 6.94 (d, *J* = 8.9 Hz, 2H), 5.26-5.21 (m, 1H), 4.14-4.09 (m, 4H), 2.06-1.57 (m, 16H). $\mathbf{R_f}$ = 0.23 (hexanes/ethyl acetate = 3:1).

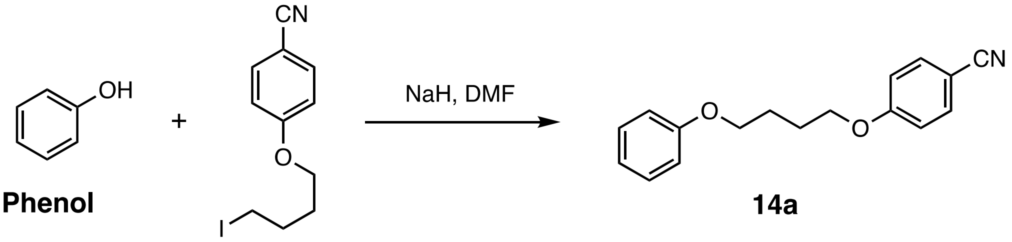

4-(4-phenoxybutoxy)benzonitrile (14a)

Prepared according to the **general procedure E** using **phenol** and **(L6)**. White solid (41 mg, 47.2% isolated yield).

^{1}HNMR: (500 MHz, $CDCl_3$): δ 7.59 (d, *J* = 8.9 Hz, 2H), 7.33-7.29 (m, 2H), 6.99-6.91 (m, 5H), 4.10 (t, *J* = 6.0 Hz, 2H), 4.06 (t, *J* = 6.0 Hz, 2H), 2.05-1.99 (m, 4H). $\mathbf{R_f}$ = 0.49 (hexanes/ethyl acetate = 3:1).

General Procedure F (Tetrazole Formation)

To a flame dried reaction flask under was charged with one of the following compounds, **(4f), (5c-7c), (8a-12a), (13e),** or **(14a)** (0.2 mmol, 1 equiv.), NaN_3 (130 mg, 2 mmol, 10 equiv.), NH_4Cl (107 mg, 2 mmol, 10 equiv.), and anhydrous DMF (3.6 mL). The reaction mixture was heated to 120°C and allowed to stir for 16 hours. The mixture was then concentrated and dissolved in ethyl acetate (15 mL). The organic solution was washed with 1M HCl (10 mL), dried with brine, dried over anhydrous Na_2SO_4, and concentrated. The crude product was purified by column chromatography (SiO_2: eluent: hexanes/ethyl acetate = 1:1→1:3) to afford pure products.

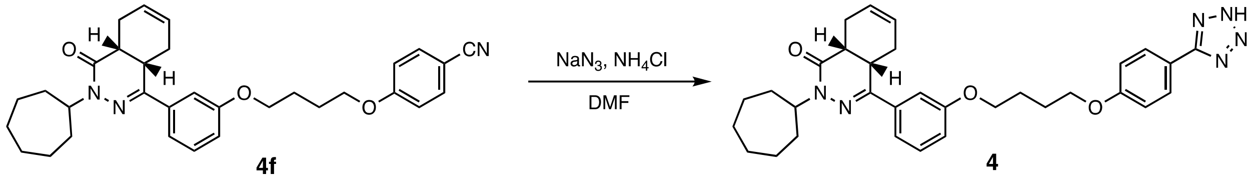

(4a*S*,8a*R*)-4-(3-(4-(4-(2*H*-tetrazol-5-yl)phenoxy)butoxy)phenyl)-2-cycloheptyl-4a,5,8,8a-tetrahydrophthalazin-1(2*H*)-one (4)

Prepared according to the **general procedure F** using compound **(4f)**. White solid (13 mg, 41.8% isolated yield).

^{1}HNMR: (600 MHz, $CDCl_3$): δ 8.09 (d, *J* = 8.9 Hz, 2H), 7.38-7.32 (m, 3H), 7.01 (d, *J* = 8.9 Hz, 2H), 6.96-6.94 (m, 1H), 5.75-5.63 (m, 2H), 4.85-4.80 (m, 1H), 4.12-4.09 (m, 4H), 3.33-3.29 (m, 1H), 3.01-2.97 (m, 1H), 2.78 (t, *J* = 5.6 Hz, 1H), 2.24-1.47 (m, 19H). Acidic NH proton is not observed in the HNMR. **^{13}C NMR** (126 MHz, $CDCl_3$): δ 166.6, 161.7, 159.2, 154.5, 136.4, 129.7, 129.3, 128.4, 125.8, 124.0, 118.4, 115.7, 115.2, 114.8, 112.0, 67.8, 67.5, 56.6, 34.7, 33.1, 32.9, 31.2, 28.4, 25.9, 24.9, 23.1, 22.4. **HRMS (ESI)** m/z calculated for $C_{32}H_{38}N_6O_3Na$ $[M+Na]^+$: 577.2903; found 577. 2880. **R_f** = 0.25 (hexanes/ethyl acetate = 1:1).

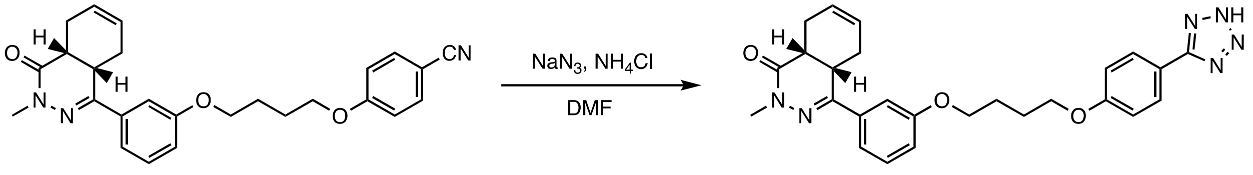

(4a*S*,8a*R*)-4-(3-(4-(4-(2*H*-tetrazol-5-yl)phenoxy)butoxy)phenyl)-2-methyl-4a,5,8,8a-tetrahydrophthalazin-1(2*H*)-one (5)

Prepared according to the **general procedure F** using compound **(5c)**. White solid (60 mg, 70.5% isolated yield).

^{1}HNMR: (600 MHz, $CDCl_3$): δ 8.09 (d, *J* = 8.9 Hz, 2H), 7.37-7.30 (m, 3H), 7.00 (d, *J* = 8.9 Hz, 2H), 6.95-6.94 (m, 1H), 5.74-5.63 (m, 2H), 4.10-4.09 (m, 4H), 3.48 (s, 3H), 3.36-3.32 (m, 1H), 3.00-2.95 (m, 1H), 2.82 (t, *J* = 6.7 Hz, 1H), 2.25-1.99 (m, 7H). Acidic NH proton is not observed in the HNMR. **^{13}C NMR**: (126 MHz, $CDCl_3$): δ 168.5, 161.5, 159.3, 155.2, 135.8, 129.8, 129.1, 125.6, 124.0, 118.4, 116.3, 116.0, 115.1, 111.7, 67.7, 67.5, 37.2, 34.4, 31.8, 25.9, 25.8, 23.4, 22.1. **HRMS (ESI)** m/z calculated for $C_{26}H_{28}N_6O_3H$ $[M+H]^+$: 473.2301; found 473.2316. **R_f** = 0.25 (hexanes/ethyl acetate = 1:1).

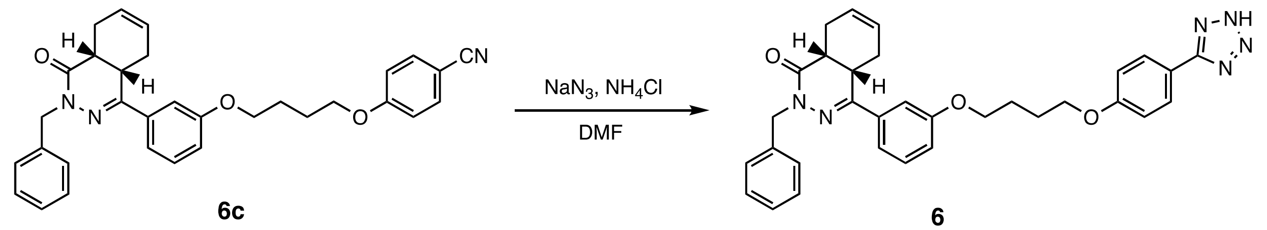

6c 6

(4a*S*,8a*R*)-4-(3-(4-(4-(2*H*-tetrazol-5-yl)phenoxy)butoxy)phenyl)-2-benzyl-4a,5,8,8a-tetrahydrophthalazin-1(2*H*)-one (6)

Prepared according to the **general procedure F** using compound **(6c)**. White solid (27.3 mg, 64.6% isolated yield).

^{1}HNMR: (600 MHz, $CDCl_3$): δ 8.06 (d, *J* = 8.9 Hz, 2H) 7.36-7.21 (m, 8H), 6.98-6.93 (m, 3H), 5.74-5.61 (m, 2H), 5.17 (d, *J* = 14.5 Hz, 1H), 4.94 (d, *J* = 14.5 Hz, 1H), 4.10-4.07 (m, 4H) 3.36-3.32 (m, 1H), 3.01-2.97 (m, 1H), 2.87 (t, *J* = 6.0 Hz, 1H), 2.24-1.94 (m, 7H). Acidic NH proton is not observed in the HNMR. **^{13}C NMR:** (126 MHz, $CDCl_3$): δ 168.1, 161.5, 159.2, 155.0, 137.4, 135.9, 129.7, 129.1, 128.4, 128.2, 127.4, 125.6, 123.9, 118.5, 116.2, 115.2, 111.9, 67.7, 67.5, 52.9, 34.6, 31.8, 25.9, 25.8, 23.3, 22.2. **HRMS (ESI)** m/z calculated for $C_{32}H_{32}N_6O_3Na$ $[M+Na]^+$: 571.2433; found 571.2415. **R_f** = 0.25 (hexanes/ethyl acetate = 1:1).

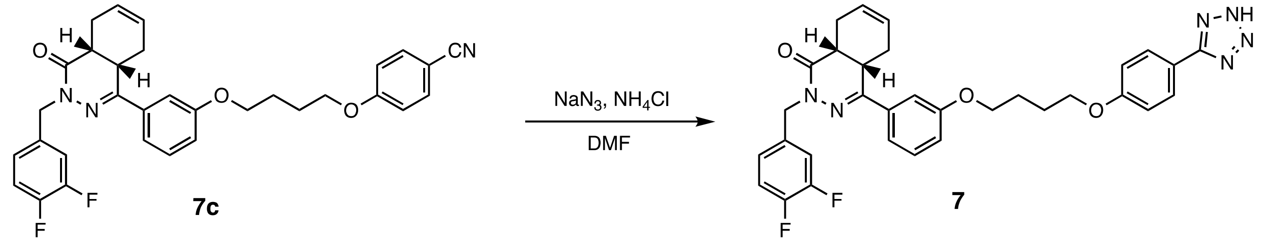

7c 7

(4a*S*,8a*R*)-4-(3-(4-(4-(2*H*-tetrazol-5-yl)phenoxy)butoxy)phenyl)-2-(3,4-difluorobenzyl)-4a,5,8,8a-tetrahydrophthalazin-1(2*H*)-one (7)

Prepared according to the **general procedure F** using compound **(7c)**. White solid (13.8 mg, 14.8% isolated yield).

^{1}HNMR: (600 MHz, $CDCl_3$): δ 8.06 (d, *J* = 8.8 Hz, 2H) 7.34-7.32 (m, 3H), 7.21-7.18 (m, 1H), 7.09-6.95 (m, 5H), 5.76-5.63 (m, 2H), 5.06 (d, *J* = 14.5 Hz, 1H), 4.89 (d, *J* = 14.5 Hz, 1H), 4.12-4.08 (m, 4H) 3.37-3.33 (m, 1H), 3.00-2.95 (m, 1H), 2.85 (t, *J* = 5.8 Hz, 1H), 2.24-1.90 (m, 7H). Acidic NH proton is not observed in the HNMR. **^{13}C NMR:** (126 MHz, $CDCl_3$) δ 168.1, 161.7, 159.3, 155.3, 150.1 (dd, $J_{C\text{-}F}$ = 248.8, 13.0 Hz), 149.8 (dd, $J_{C\text{-}F}$ = 248.2, 12.9 Hz), 135.7, 134.4, 129.8, 129.2, 125.7, 124.5 (dd, $J_{C\text{-}F}$ = 6.5, 3.7 Hz), 124.5, 123.8, 118.5, 117.4 (d, $J_{C\text{-}F}$ = 23.0 Hz), 117.2 (d, $J_{C\text{-}F}$ = 22.6 Hz), 116.2, 115.7, 115.2, 111.9, 67.7, 67.5, 51.9, 34.6, 31.8, 25.9, 25.8, 23.3, 22.1. **HRMS (ESI)** m/z calculated for $C_{32}H_{30}F_2N_6O_3Na$ $[M+Na]^+$: 607.2245; found 607.2264. **R_f** = 0.25 (hexanes/ethyl acetate = 1:1).

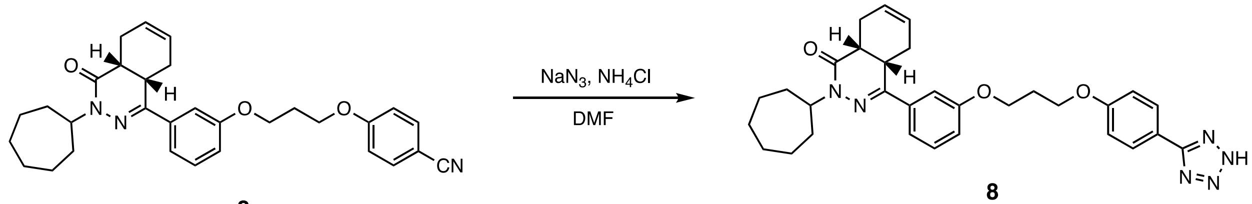

8a **8**

(4a*S*,8a*R*)-4-(3-(3-(4-(2*H*-tetrazol-5-yl)phenoxy)propoxy)phenyl)-2-cycloheptyl-4a,5,8,8a-tetrahydrophthalazin-1(2*H*)-one (8)

Prepared according to the **general procedure F** using compound **(8a)**. White solid (13.1 mg, 53.8% isolated yield).

^{1}HNMR: (600 MHz, $CDCl_3$): δ 8.09 (d, *J* = 8.9 Hz, 2H), 7.38-7.32 (m, 3H), 7.04 (d, *J* = 8.9 Hz, 2H), 6.98-6.96 (m, 1H), 5.75-5.63 (m, 2H), 4.84-4.80 (m, 1H), 4.26-4.22 (m, 4H), 3.32-3.38 (m, 1H), 3.01-2.97 (m, 1H), 2.77 (t, *J* = 6.1 Hz, 1H), 2.35-2.31 (m, 2H). 2.24-1.43 (m, 15H). Acidic NH proton is not observed in the HNMR. **^{13}C NMR:** (126 MHz, $CDCl_3$): δ 166.7, 161.4, 159.1, 154.6, 136.4, 129.8, 129.1, 125.8, 124.0, 118.5, 115.7, 115.2, 112.0, 64.6, 64.2, 56.7, 34.7, 33.1, 32.9, 31.1, 29.2, 28.4, 25.0, 23.1. **HRMS (ESI)** m/z calculated for $C_{31}H_{36}N_6O_3Na$ $[M+Na]^+$: 563.2747; found 563.2726. **R_f** = 0.25 (hexanes/ethyl acetate = 1:1).

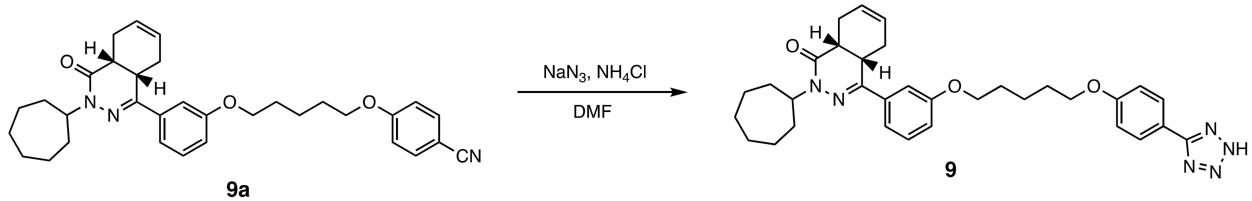

9a **9**

(4a*S*,8a*R*)-4-(3-((5-(4-(2*H*-tetrazol-5-yl)phenoxy)pentyl)oxy)phenyl)-2-cycloheptyl-4a,5,8,8a-tetrahydrophthalazin-1(2*H*)-one (9)

Prepared according to the **general procedure F** using compound **(9a)**. White solid (19 mg, 66.8% isolated yield).

^{1}HNMR: (600 MHz, $CDCl_3$): δ 8.10 (d, *J* = 8.9 Hz, 2H), 7.38-7.31 (m, 3H), 7.01 (d, *J* = 8.9 Hz, 2H), 6.95-6.94 (m, 1H), 5.75-5.63 (m, 2H), 4.86-4.81 (m, 1H), 4.07-4.05 (m, 4H), 3.33-3.29 (m, 1H), 3.01-2.98 (m, 1H), 2.78 (t, *J* = 6.1 Hz, 1H), 2.24-1.45 (m, 21H). Acidic NH proton is not observed in the HNMR. **^{13}C NMR:** (126 MHz, $CDCl_3$): δ 166.7, 161.7, 159.3, 154.7, 136.3, 129.7, 129.2, 125.8, 124.0, 118.3, 115.8, 115.2, 112.1, 68.0, 67.8, 56.6, 34.7, 33.1, 32.9, 31.1, 29.7, 29.0, 28.4, 24.9, 23.1, 22.8. **HRMS (ESI)** m/z calculated for $C_{33}H_{40}N_6O_3Na$ $[M+Na]^+$: 591.3060; found 591.3064. **R_f** = 0.25 (hexanes/ethyl acetate = 1:1).

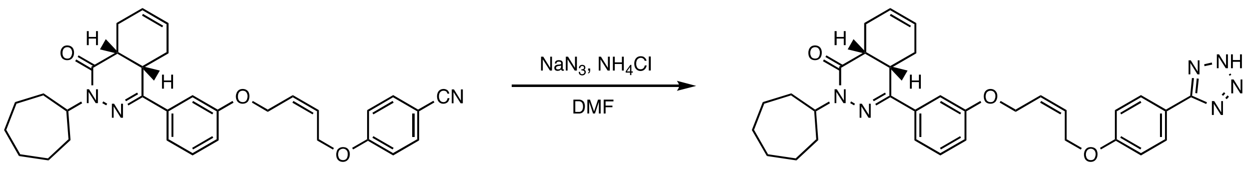

10a **10**

(4a*S*,8a*R*)-4-(3-(((*Z*)-4-(4-(2*H*-tetrazol-5-yl)phenoxy)but-2-en-1-yl)oxy)phenyl)-2-cycloheptyl-4a,5,8,8a-tetrahydrophthalazin-1(2*H*)-one (10)

Prepared according to the **general procedure F** using compound **(10a)**. White solid (8.1 mg, 38.6% isolated yield).

^{1}**HNMR** (500 MHz, $CDCl_3$): δ 8.07 (d, *J* = 8.6 Hz, 2H), 7.42-7.33 (m, 3H), 7.04-6.97 (m, 3H), 6.02-5.97 (m, 2H), 5.73-5.65 (m, 2H), 4.84-4.75 (m, 5H), 3.31-3.27 (m, 1H), 2.99-2.96 (m, 1H), 2.76-2.74 (m, 1H), 2.21-1.46 (m, 15H). Acidic NH proton is not observed in the HNMR. ^{13}**C NMR** (126 MHz, $CDCl_3$): δ 166.6, 158.6, 154.3, 136.5, 129.8, 129.4, 128.9, 128.1, 125.9, 123.9, 118.8, 116.1, 115.4, 112.1, 64.5, 64.3, 56.6, 34.7, 33.2, 32.9, 31.2, 29.7, 28.3, 25.0. **HRMS (ESI)** m/z calculated for $C_{32}H_{36}N_6O_3Na$ $[M+Na]^+$: 575.2747; found 575.2739. $\mathbf{R_f}$ = 0.25 (hexanes/ethyl acetate = 1:1).

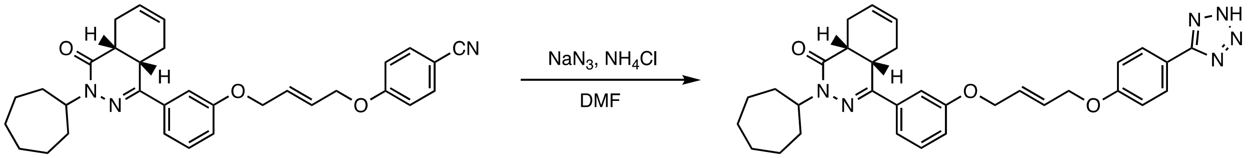

11a **11**

(4a*S*,8a*R*)-4-(3-((€-4-(4-(2*H*-tetrazol-5-yl)phenoxy)but-2-en-1-yl)oxy)phenyl)-2-cycloheptyl-4a,5,8,8a-tetrahydrophthalazin-1(2*H*)-one (11)

Prepared according to the **general procedure F** using compound **(11a)**. White solid (11.3 mg, 37.9% isolated yield).

^{1}**HNMR:** (600 MHz, $CDCl_3$): δ 8.07 (d, *J* = 8.9 Hz, 2H), 7.42-7.32 (m, 3H), 7.03 (d, *J* = 8.9 Hz, 2H), 6.97-6.95 (m, 1H), 6.13-6.12 (m, 2H), 5.75-5.63 (m, 2H), 4.84-4.79 (m, 1H), 4.66-4.65 (m, 4H), 3.32-3.28 (m, 1H), 3.00-2.97 (m, 1H), 2.76 (t, *J* = 6.1 Hz, 1H), 2.23-1.44 (m, 15H). Acidic NH proton is not observed in the HNMR. ^{13}**C NMR:** (126 MHz, $CDCl_3$): δ 166.7, 160.9, 158.7, 154.4, 136.4, 129.8, 129.0, 128.7, 128.0, 125.8, 123.9, 118.7, 116.1, 115.4, 112.1, 67.8, 67.6, 56.6, 34.7, 33.2, 32.9, 31.1, 28.3, 25.0, 24.9, 23.1. **HRMS (ESI)** m/z calculated for $C_{32}H_{36}N_6O_3Na$ $[M+Na]^+$: 575.2747; found 575.2739. $\mathbf{R_f}$ = 0.25 (hexanes/ethyl acetate = 1:1).

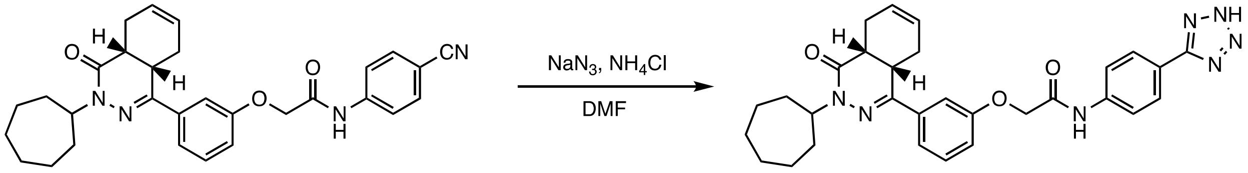

12a **12**

***N*-(4-(2*H*-tetrazol-5-yl)phenyl)-2-(3-((4a*R*,8a*S*)-3-cycloheptyl-4-oxo-3,4,4a,5,8,8a-hexahydrophthalazin-1-yl)phenoxy)acetamide (12)**

Prepared according to the **general procedure F** using compound **(12a)**. White solid (29 mg, 42.9% isolated yield).

^{1}HNMR: (600 MHz, CD_3OD): δ 7.99 (d, *J* = 8.8 Hz, 2H), 7.88 (d, *J* = 8.8 Hz, 2H), 7.58-7.38 (m, 3H), 7.13-7.11 (m, 1H), 5.74-5.62 (m, 2H), 4.77-4.70 (m, 3H), 3.47-3.41 (m, 1H), 2.88-2.84 (m, 1H), 2.77 (m, 1H), 2.26-1.48 (m, 15H). Acidic NH protons are not observed in the HNMR. **^{13}C NMR** (126 MHz, CD_3OD) δ 169.6, 168.3, 159.7, 156.2, 142.2, 137.9, 131.0, 129.0, 126.9, 124.9, 121.9, 121.0, 120.8, 117.8, 112.8, 68.7, 57.7, 35.9, 34.2, 34.0, 32.2, 29.4, 26.1, 24.1. **HRMS (ESI)** m/z calculated for $C_{30}H_{33}N_7O_3Na$ $[M+Na]^+$: 562.2543; found 562.2567. **R_f** = 0.14 (hexanes/ethyl acetate = 1:1).

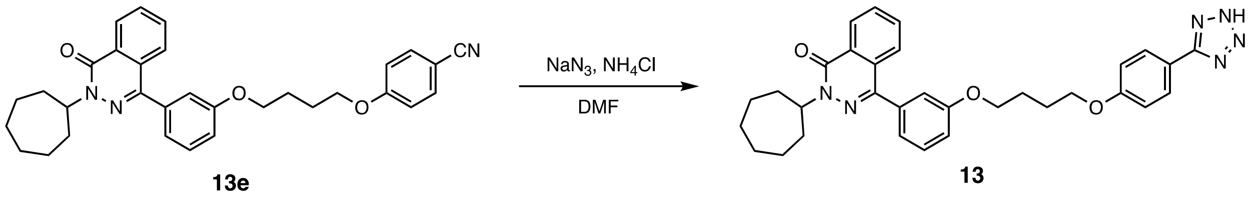

4-(3-(4-(4-(2*H*-tetrazol-5-yl)phenoxy)butoxy)phenyl)-2-cycloheptylphthalazin-1(2*H*)-one (13)

Prepared according to the **general procedure F** using compound **(13e)**. White solid (29 mg, 47.0% isolated yield).

^{1}HNMR: (600 MHz, $CDCl_3$): δ 8.52 (d, *J* = 9.7 Hz, 1H), 8.02 (d, *J* = 9.7 Hz, 2H), 7.79-7.75 (m, 3H), 7.45-7.42 (m, 1H), 7.17 (d, *J* = 9.1 Hz, 1H), 7.10-7.04 (m, 2H), 6.93 (d, *J* = 9.2 Hz, 2H), 5.26-5.21 (m, 1H), 4.15-4.09 (m, 4H), 2.08-1.54 (m, 16H). Acidic NH proton is not observed in the HNMR. **^{13}C NMR:** (126 MHz, $CDCl_3$): δ 161.3, 158.9, 147.3, 136.6, 133.0, 131.5, 129.7, 129.1, 128.6, 127.2, 126.5, 121.9, 115.5, 115.0, 67.6, 67.5, 59.0, 33.7, 27.9, 25.7, 25.0. **HRMS (ESI)** m/z calculated for $C_{32}H_{34}N_6O_3Na$ $[M+Na]^+$: 573.2590; found 573.2583. **R_f** = 0.29 (hexanes/ethyl acetate = 1:1).

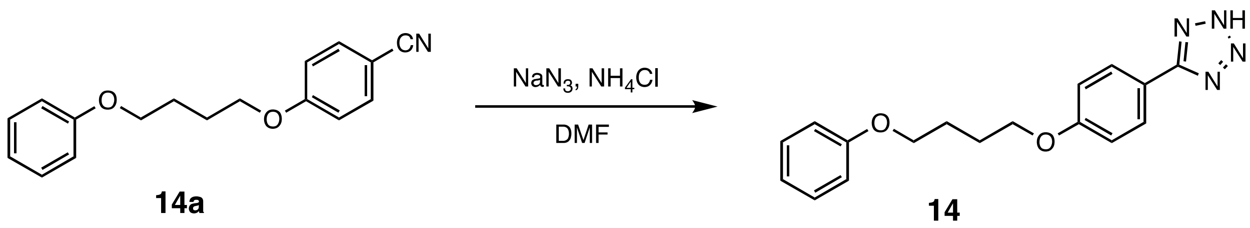

5-(4-(4-phenoxybutoxy)phenyl)-2*H*-tetrazole (14)

Prepared according to the **general procedure F** using compound **(14a)**. White solid (30 mg, 63.2% isolated yield).

^{1}HNMR: (600 MHz, CD_3OD): δ 7.94 (d, *J* = 8.9 Hz, 2H), 7.25 (dd, *J* = 9.0, 8.8 Hz, 2H), 7.13 (d, *J* = 9.0 Hz, 2H), 6.92-6.89 (m, 3H), 4.16 (t, *J* = 6.0 Hz, 2H), 4.06 (t, *J* = 6.1 Hz, 2H), 2.02-1.97 (m, 4H). Acidic NH proton is not observed in the HNMR. **^{13}C NMR**: (126 MHz, CD_3OD): δ 163.1, 160.5, 130.4, 129.9, 121.6, 117.4, 116.4, 115.5, 69.1, 68.5, 27.1. **HRMS (ESI)** m/z calculated for $C_{17}H_{18}N_4O_2Na$ $[M+Na]^+$: 333.1328; found 333.1316. **R_f** = 0.25 (hexanes/ethyl acetate = 1:1).

6. Chemical Synthesis and Compound Characterization of Difunctional Linkers

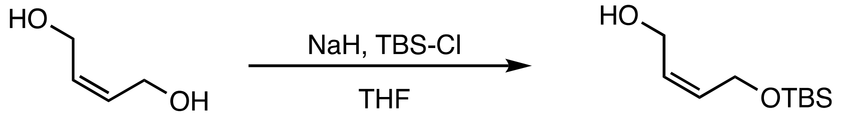

L1a

(*Z*)-4-((*tert*-butyldimethylsilyl)oxy)but-2-en-1-ol (L1a)

Synthesis is previously reported.[3]

^{1}HNMR: (600 MHz, $CDCl_3$): δ 5.74-5.65 (m, 2H), 4.26 (d, *J* = 5.3 Hz, 2H), 4.21 (d, *J* = 5.9 Hz, 2H), 0.91 (s, 9H), 0.09 (s, 6H). Alcohol peak is not present in the NMR. Our characterization data matches with prior literature data.[3]

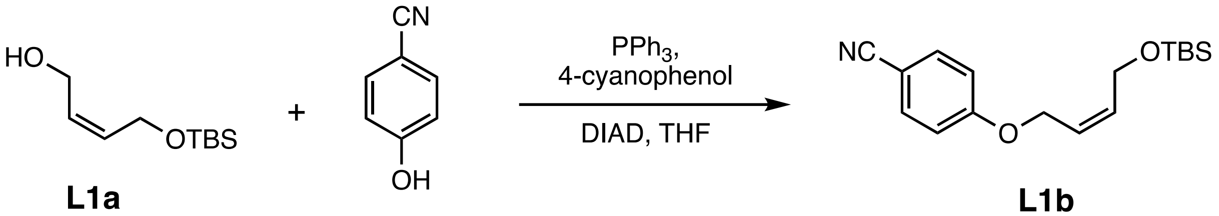

L1a **4-cyanophenol** **L1b**

(*Z*)-*tert*-butyldimethyl((4-(*p*-tolyloxy)but-2-en-1-yl)oxy)silane (L1b)

To a flame dried reaction flask, under N_2, was added compound **(L1a)** (97 mg, 0.48 mmol, 1.0 equiv.), 4-cyanophenol (62.5 mg, 0.52 mmol, 1.1 equiv.), PPh_3 (151.1 mg, 0.58 mmol, 1.2 equiv.), and anhydrous THF (1.6 mL) and cooled to 0°C. Diisopropyl azodicarboxylate (DIAD) (0.114 mL, 0.48 mmol, 1.2 equiv.) was added dropwise to the reaction mixture and was allowed to stir for 30 min at room temperature. The solution was concentrated, and the crude product was purified by column chromatography (SiO_2: eluent: hexanes/ethyl acetate = 3:1) to afford the title compounds. Yellow oil (141 mg, 96.7 % isolated yield).

^{1}HNMR: (500 MHz, $CDCl_3$): δ 7.54 (d, *J* = 9.0 Hz, 2H), 6.93 (d, *J* = 9.0 Hz, 2H), 5.78-5.63 (m, 2H), 4.70 (d, *J* = 6.1 Hz, 2H), 4.29 (d, *J* = 5.6 Hz, 2H), 0.88 (s, 9H), 0.06 (s, 6H).

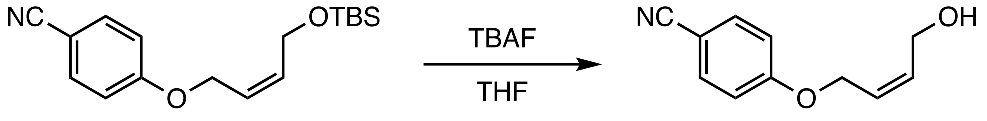

L1b **L1**

(*Z*)-4-((4-hydroxybut-2-en-1-yl)oxy)benzonitrile (L1)

To a flame dried reaction flask, under N_2, was added compound **(L1b)** (141 mg, 0.46 mmol, 1.0 equiv.) and anhydrous THF (2.3 mL) and the solution was cooled to 0°C. Tetrabutylammonium fluoride 1M in THF (0.552 mL, 0.552 mmol, 1.2 equiv.) was added and the reaction mixture was allowed to stir at 0°C for 1h. The mixture was diluted in ethyl acetate (15 mL), washed with brine, dried over Na_2SO_4, and concentrated. The crude product was purified by column chromatography (SiO_2: eluent: hexanes/ethyl acetate = 3:1→1:1) to afford the title compound. White solid (42 mg, 43.5 % isolated yield).

^{1}HNMR: (600 MHz, $CDCl_3$): δ 7.54 (d, *J* = 8.9 Hz, 2H), 6.93 (d, *J* = 8.9 Hz, 2H), 5.89-5.73 (m, 2H), 4.68 (d, *J* = 6.1 Hz, 2H), 4.27 (d, *J* = 6.3 Hz, 2H), 2.30 (br, 1H, OH).

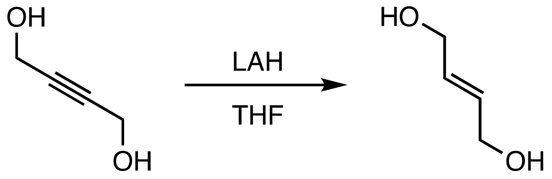

L2a

€-but-2-ene-1,4-diol (L2a)

Synthesis is previously reported.[4]

^{1}HNMR: (600 MHz, $CDCl_3$): δ 5.91-5.89 (m, 2H), 4.18 (m, 4H). Alcohol peaks are not present in the NMR. Our characterization data matches with prior literature data.[4]

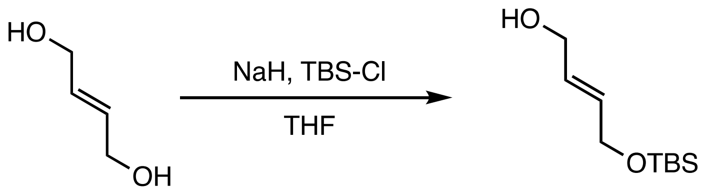

L2a **L2b**

€-4-((*tert*-butyldimethylsilyl)oxy)but-2-en-1-ol (L2b)

To a flame dried reaction flask, under N_2, charged with NaH (203 mg, 5.1 mmol, 60% in oil, 1.0 equiv.) and anhydrous THF (10 mL), was added compound **(L2a)** (448 mg, 5.1 mmol, 1.0 equiv.) and the mixture was allowed to stir for 1 h at room temperature. *tert*-Butyldimethylsilyl chloride (TBS-Cl) (768 mg, 5.1 mmol, 1.0 equiv.) was added to the mixture and was then allowed to stir for 24 hours at room temperature. The solution was diluted in Et_2O (30 mL) and was washed with 10 % aqueous K_2CO_3 (20 mL). The organic layer was then dried with brine, dried over Na_2SO_4, and concentrated. The crude product was purified by column chromatography (SiO_2: eluent: hexanes/ethyl acetate = 2:1) to afford the title compound. Yellow oil (118.4 mg, 11.5 % isolated yield).

^{1}HNMR: (500 MHz, $CDCl_3$): δ 5.87-5.74 (m, 2H), 4.17-4.15 (m, 2H), 4.13 (dd, J = 5.13, 1.4 Hz, 2H), 1.64 (br, 1H, OH), 0.89 (s, 9H), 0.05 (s, 6H).

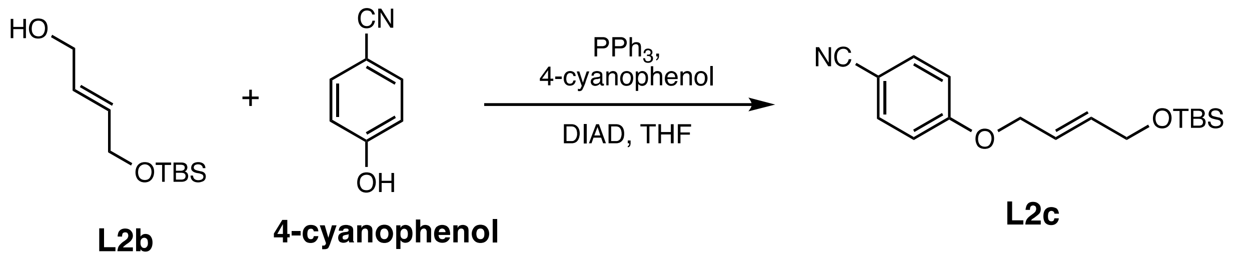

€-*tert*-butyldimethyl((4-(*p*-tolyloxy)but-2-en-1-yl)oxy)silane (L2c)

To a flame dried reaction flask, under N_2, was added compound **(L2b)** (118.4 mg, 0.59 mmol, 1.0 equiv.), 4-cyanophenol (76.7 mg, 0.64 mmol, 1.1 equiv.), PPh_3 (184.1 mg, 0.70 mmol, 1.2 equiv.), and anhydrous THF (2 mL) and cooled to 0°C. Diisopropyl azodicarboxylate (DIAD) (0.138 mL, 0.70 mmol, 1.2 equiv.) was added dropwise to the reaction mixture and was allowed to stir for 30 min at room temperature. The solution was concentrated, and the crude product was purified by column chromatography (SiO_2: eluent: hexanes/ethyl acetate = 5:1) to afford the title compound. Yellow oil (155.2 mg, 87.4 % isolated yield).

^{1}HNMR: (600 MHz, $CDCl_3$): δ 7.54 (d, J = 9.1 Hz, 2H), 6.93 (d, J = 9.1 Hz, 2H), 5.91-5.89 (m, 2H), 4.57-4.56 (m, 2H), 4.20-4.19 (m, 2H), 0.88 (s, 9H), 0.05 (s, 6H).

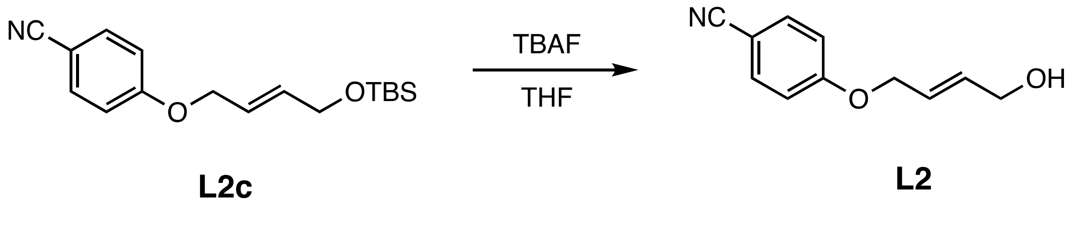

€-4-((4-hydroxybut-2-en-1-yl)oxy)benzonitrile (L2)

To a flame dried reaction flask, under N_2, was added compound **(L2c)** (155.2 mg, 0.51 mmol, 1.0 equiv.) and anhydrous THF (2.5 mL) and the solution was cooled to 0°C. Tetrabutylammonium fluoride 1M in THF (0.614 mL, 0.61 mmol, 1.2 equiv.) was added and the reaction mixture was allowed to stir at 0°C for 1h. The mixture was diluted in ethyl acetate (15

mL), washed with brine, dried over Na_2SO_4, and concentrated. The crude product was purified by column chromatography (SiO_2: eluent: hexanes/ethyl acetate = 3:1) to afford the title compound. Yellow oil (42 mg, 73.3 % isolated yield).

^{1}HNMR: (600 MHz, $CDCl_3$): δ 7.56 (d, *J* = 8.9 Hz, 2H), 6.94 (d, *J* = 8.9 Hz, 2H), 6.04-5.91 (m, 2H), 4.59 (dd, *J* = 5.5, 1.4 Hz, 2H), 4.21 (dd, *J* = 4.9, 1.5 Hz, 2H), 1.86 (br, 1H, OH).

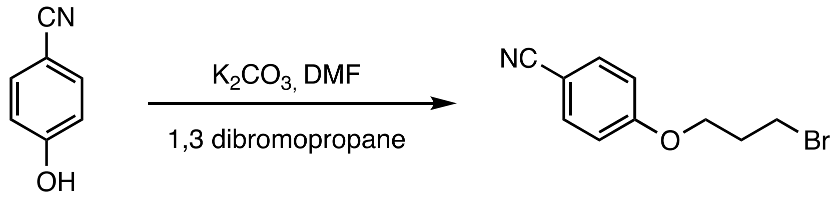

L3

4-(3-bromopropoxy)benzonitrile (L3)

To a flame dried reaction flask, under N_2, charged with K_2CO_3 (415 mg, 3.0 mmol, 2.0 equiv.), 4-cyanophenol (178.7 mg, 1.5 mmol, 1.0 equiv.), and anhydrous DMF (2 mL), was added 1,3 dibromopropane (0.459 mL, 4.5 mmol, 3.0 equiv.) and the mixture was allowed to stir for 16 h at room temperature. The solution was diluted in EtOAc (20 mL) and was washed with water (3 x 20 mL). The organic layer was then dried with brine, dried over Na_2SO_4, and concentrated. The crude product was purified by column chromatography (SiO_2: eluent: hexanes/ethyl acetate = 5:1) to afford the title compound. Yellow oil (280 mg, 77.7 % isolated yield).

^{1}HNMR: (500 MHz, $CDCl_3$): δ 7.53 (d, *J* = 9.0 Hz, 2H), 6.92 (d, *J* = 9.0 Hz, 2H), 4.12 (t, *J* = 5.9 Hz, 2H), 3.56 (t, *J* = 6.4 Hz, 2H), 2.30 (q, *J* = 6.0 Hz, 2H).

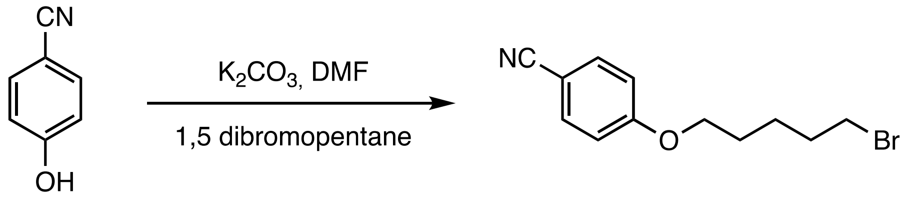

L4

4-((5-bromopentyl)oxy)benzonitrile (L4)

To a flame dried reaction flask, under N_2, charged with K_2CO_3 (415 mg, 3.0 mmol, 2.0 equiv.), 4-cyanophenol (178.7 mg, 1.5 mmol, 1.0 equiv.), and anhydrous DMF (2 mL), was added 1,5 dibromopentane (0.620 mL, 4.5 mmol, 3.0 equiv.) and the mixture was allowed to stir for 16 h at room temperature. The solution was diluted in EtOAc (20 mL) and was washed with water (3 x 20 mL). The organic layer was then dried with brine, dried over Na_2SO_4, and concentrated. The crude product was purified by column chromatography (SiO_2: eluent: hexanes/ethyl acetate = 5:1) to afford the title compound. White solid (330 mg, 82.0 % isolated yield).

^{1}HNMR: (500 MHz, $CDCl_3$): δ 7.52 (d, *J* = 9.0 Hz, 2H), 6.89 (d, *J* = 9.0 Hz, 2H), 3.97 (t, *J* = 6.2 Hz, 2H), 3.39 (t, *J* = 6.7 Hz, 2H), 1.89 (pent, *J* = 6.8 Hz, 2H), 1.79 (pent, *J* = 6.2 Hz, 2H), 1.61-1.55 (m, 2H).

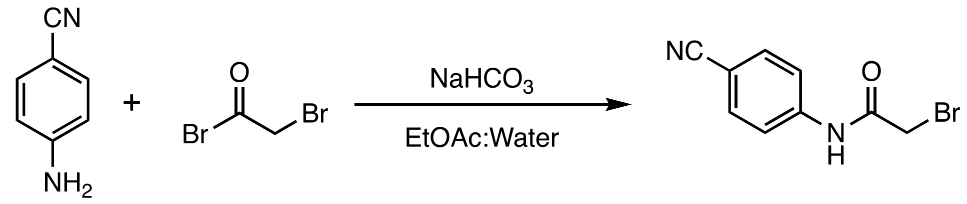

L5

2-bromo-*N*-(4-cyanophenyl)acetamide (L5)

Synthesis is previously reported.[5]

^{1}HNMR: (600 MHz, $(CD_3)_2SO$): δ 10.80 (s, 1H), 7.80 (d, *J* = 8.9 Hz, 2H), 7.76 (d, *J* = 8.9 Hz, 2H), 4.08 (s, 2H). Our characterization data matches with prior literature data.[5]

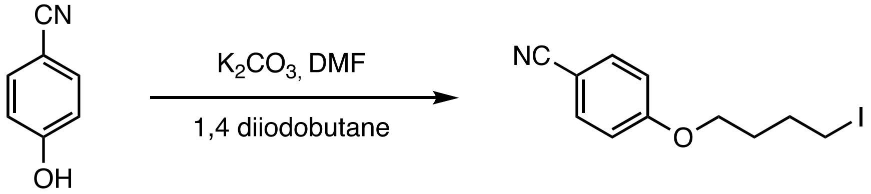

L6

4-(4-iodobutoxy)benzonitrile (L6)

To a flame dried reaction flask, under N_2, was charged with 4-cyanophenol (179 mg, 1.5 mmol, 1.0 equiv.), K_2CO_3 (415 g, 3.0 mmol, 2.0 equiv.), and anhydrous DMF (2.0 mL) and the mixture was allowed to stir for 30 min at room temperature. 1,4 diiodobutane (0.593 mL, 4.5 mmol, 3.0 equiv.) was added and the mixture was allowed to stir for 16 h at room temperature. The solution was quenched with water (10 mL) and extracted with EtOAc (3 x 20 mL). The organic layer was then washed with water (3 x 20 mL), dried with brine, dried over Na_2SO_4, and concentrated. The crude product was purified by column chromatography (SiO_2: eluent: hexanes/ethyl acetate = 3:1) to afford the title compound. White solid (356 mg, 78.8 % isolated yield).

^{1}HNMR: (500 MHz, $CDCl_3$): δ 7.54 (d, *J* = 8.8 Hz, 2H), 6.91 (d, *J* = 8.8 Hz, 2H), 4.01 (t, *J* = 6.1 Hz, 2H), 3.23 (t, *J* = 6.7 Hz, 2H), 2.03-1.97 (m, 2H), 1.93-1.87 (m, 2H).

7. Computational Docking

Crystal structure was retrieved from the RCSB Protein Data Bank for DNMT3A (PDB: 4U7T). Using SWISS-MODEL, a model of a single monomer of the catalytic domain (res 629–912) of

DNMT3A was constructed using the solved crystal structure 4U7T as a template.[6] This structure, along with the small molecules, were then prepared for docking using AutoDock Tools.[7] The small molecules were docked to the protein surface using AutoDock Vina.[7] To validate this initial setup, the native ligand SAH was docked into the active-site pocket.

8. NMR Spectra for Compounds 4-14

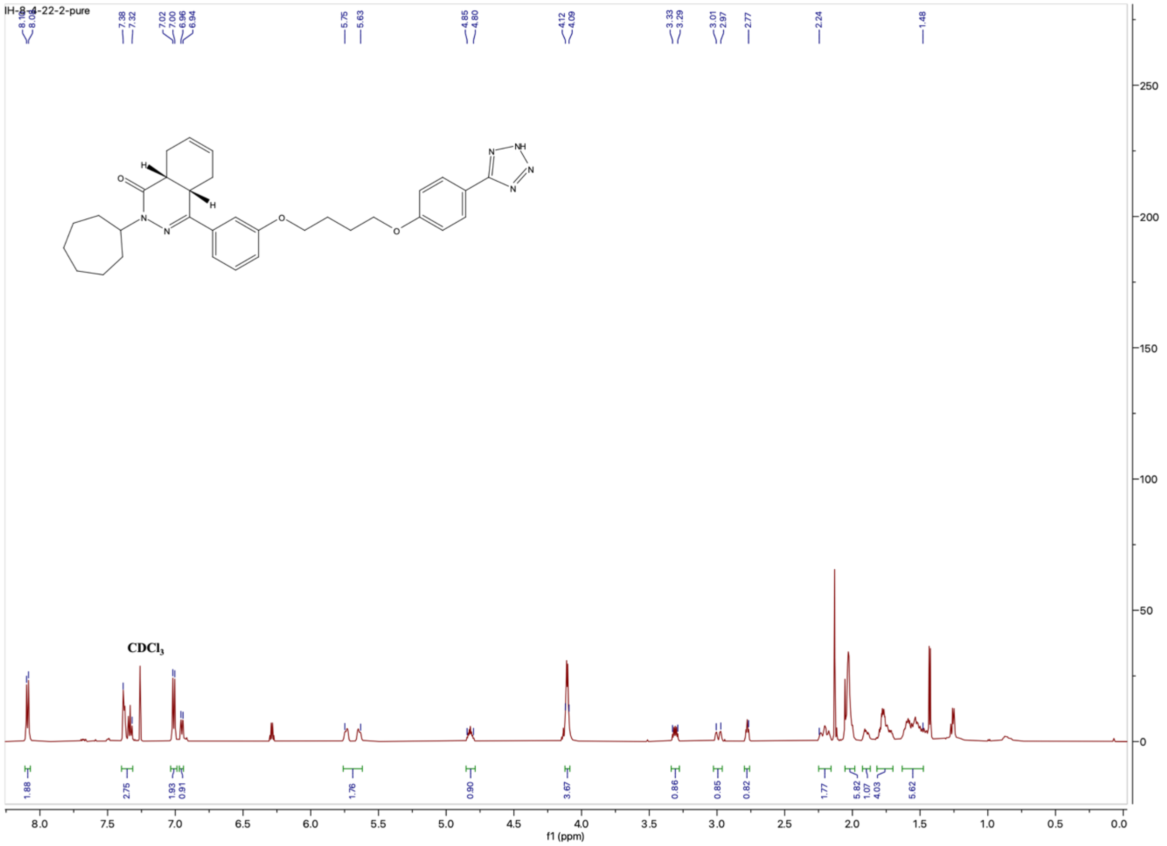

Figure S4: **^{1}**HNMR for compound **4**.

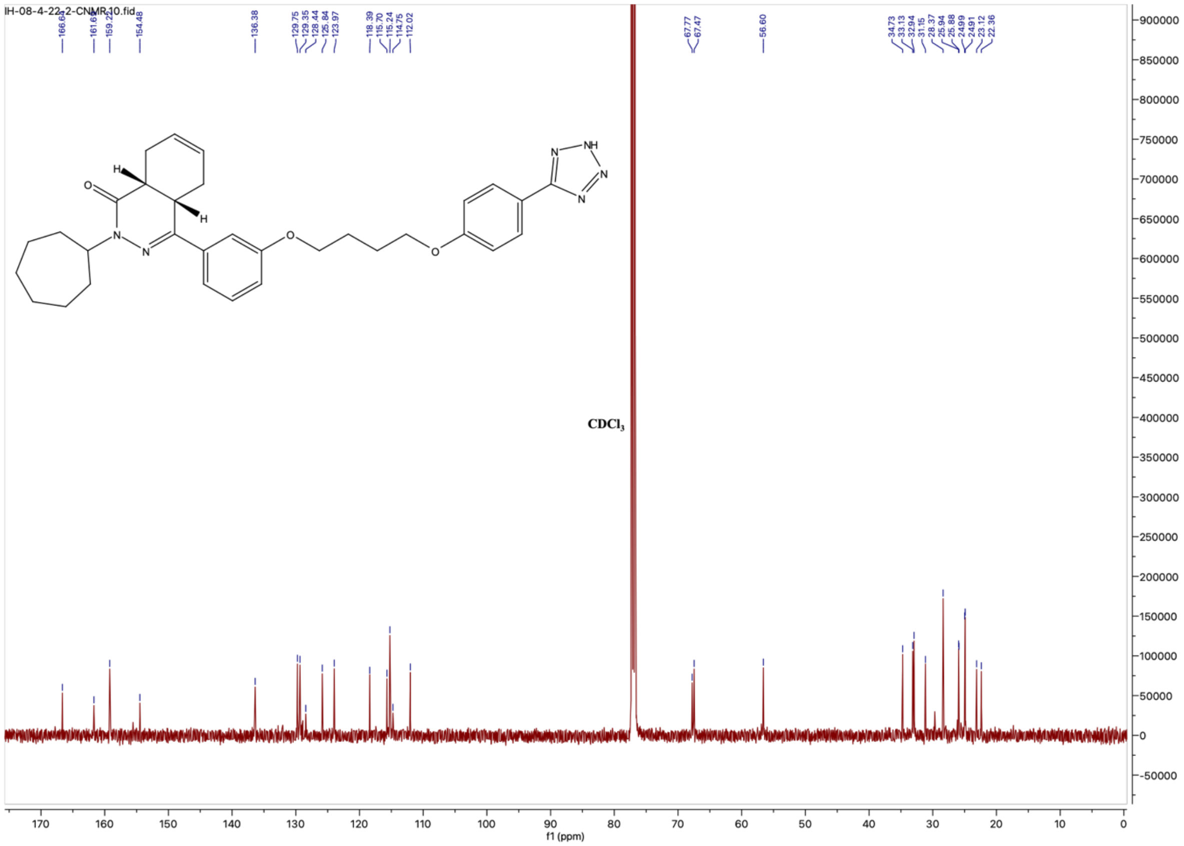

Figure S5: **13**CNMR for compound **4**.

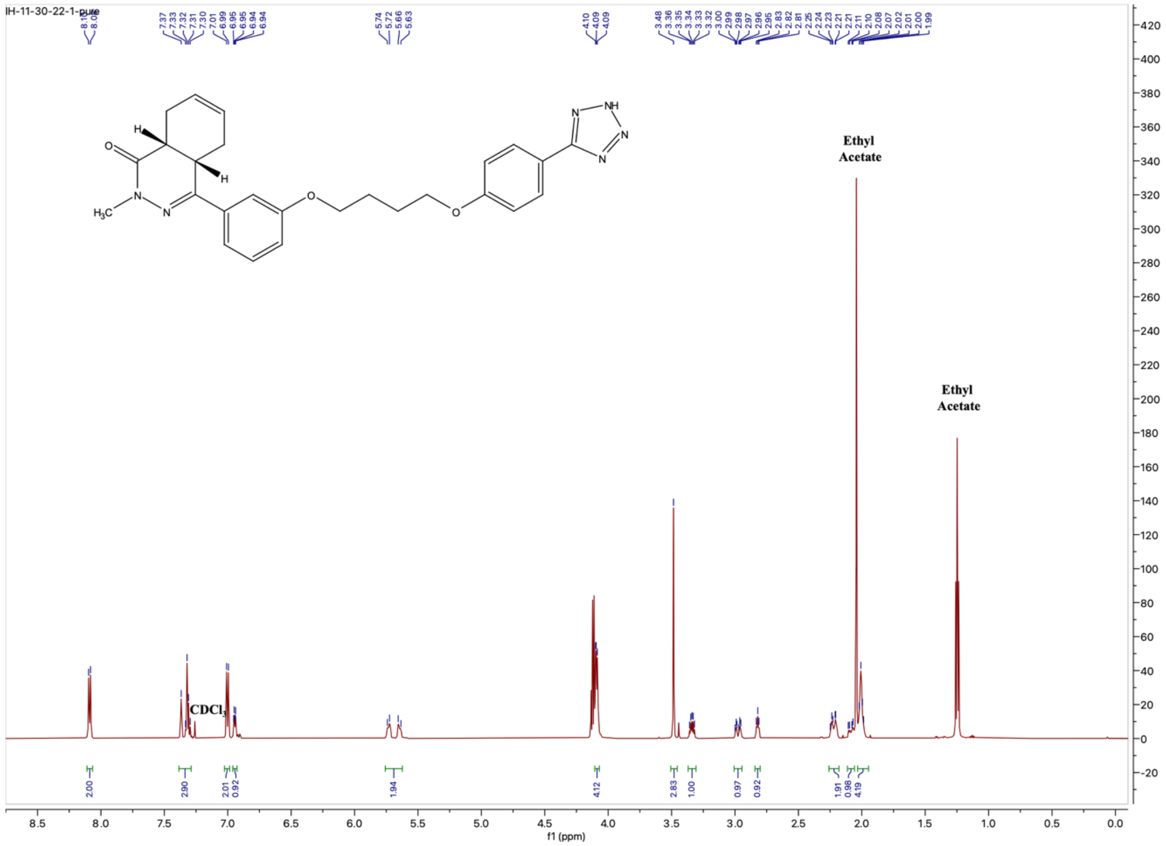

Figure S6: ^{1}HNMR for compound **5**.

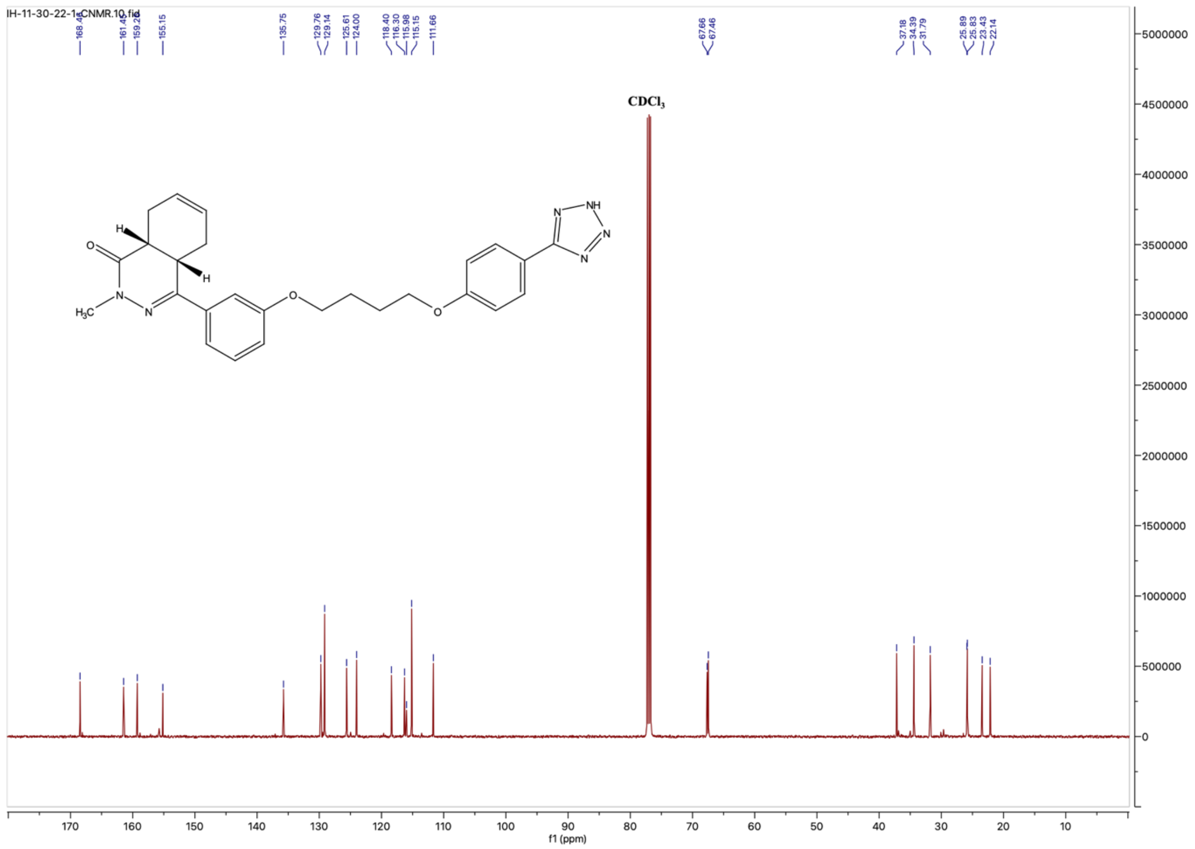

Figure S7: ^{13}CNMR for compound **5**.

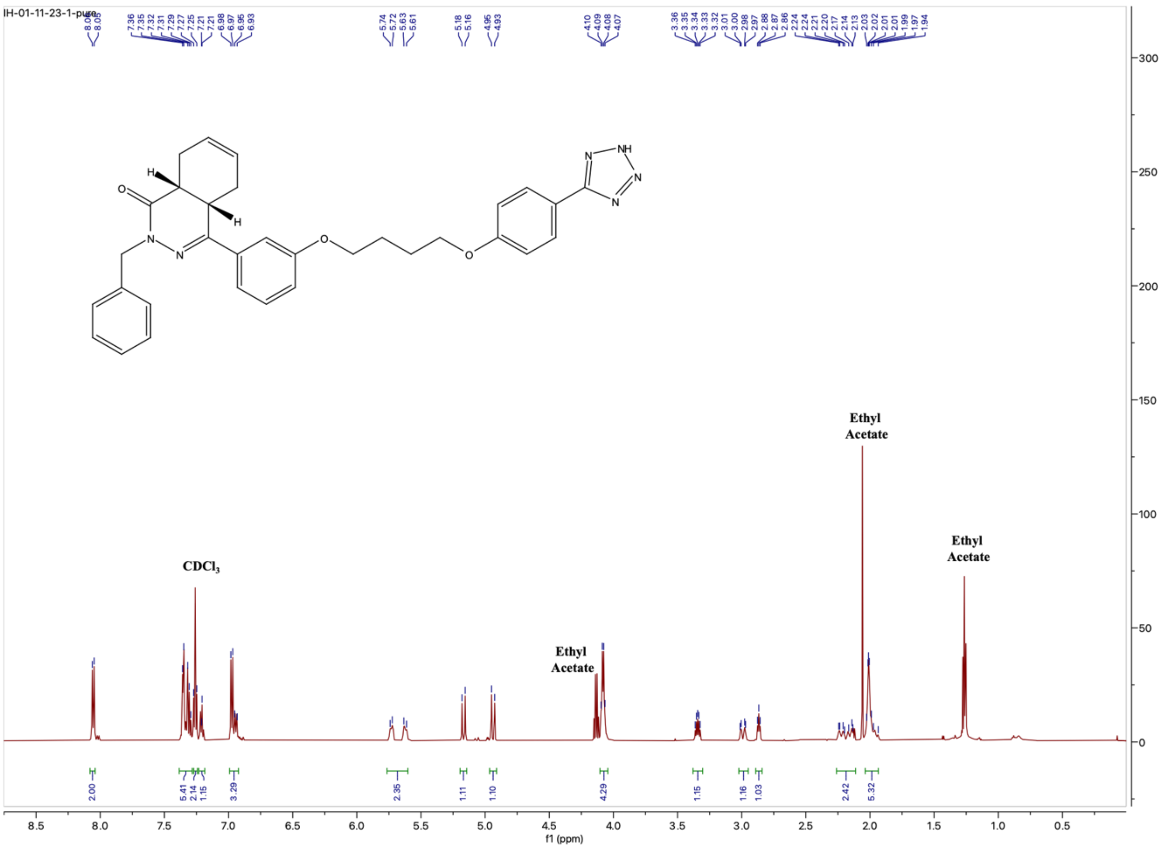

Figure S8: ^{1}HNMR for compound **6**.

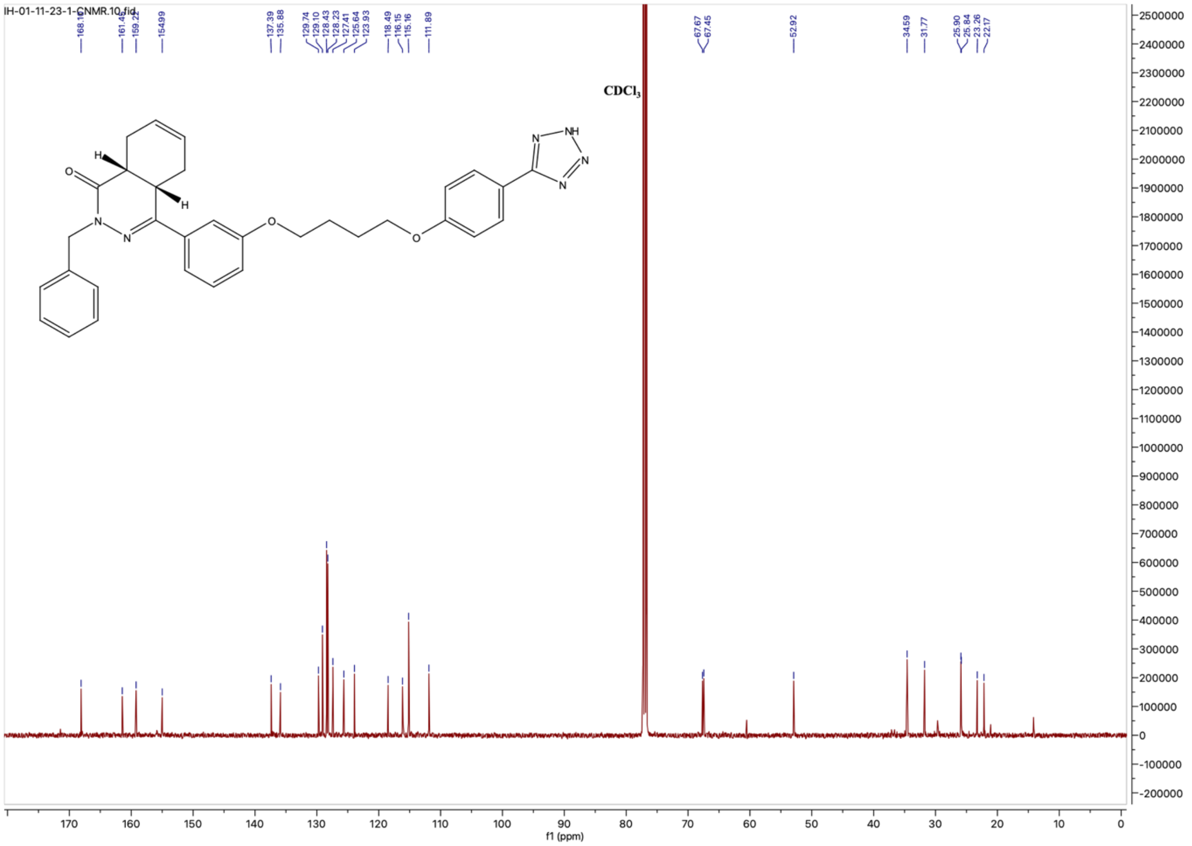

Figure S9: ^{13}CNMR for compound **6**.

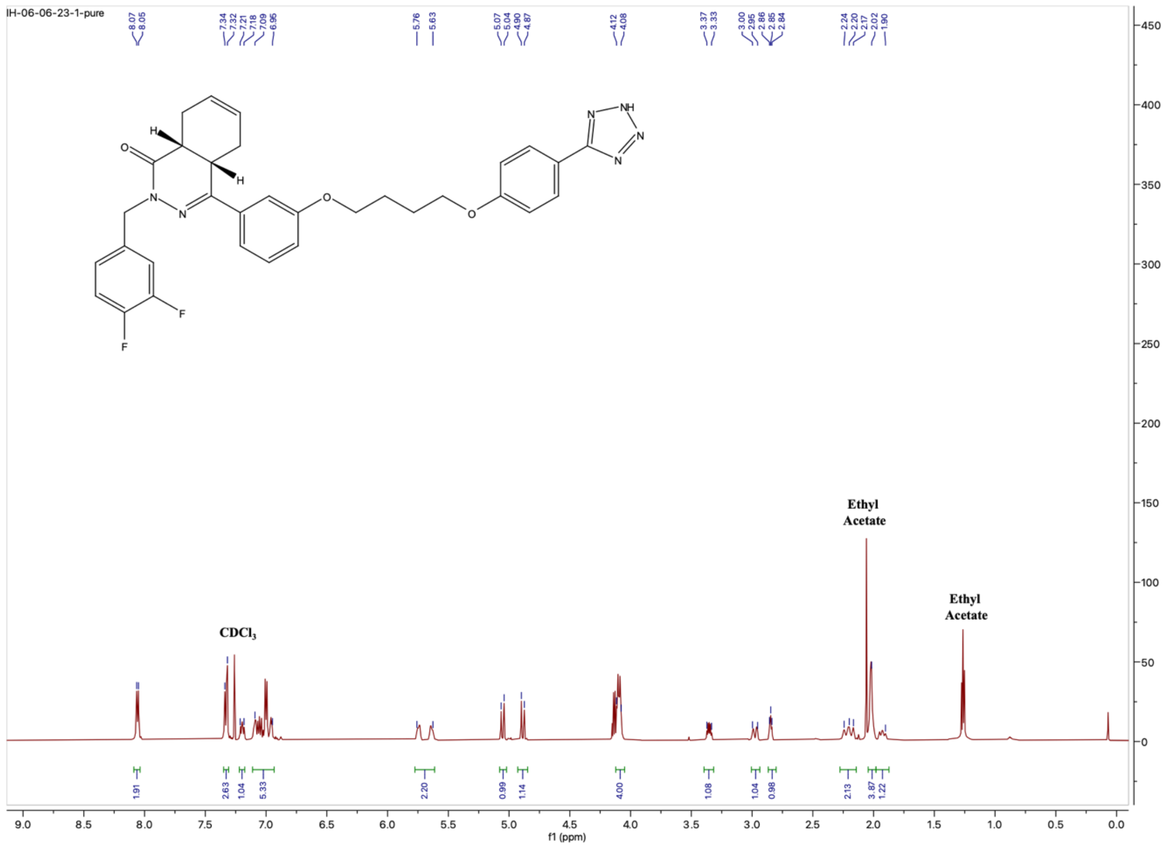

Figure S10: ^{1}HNMR for compound **7**.

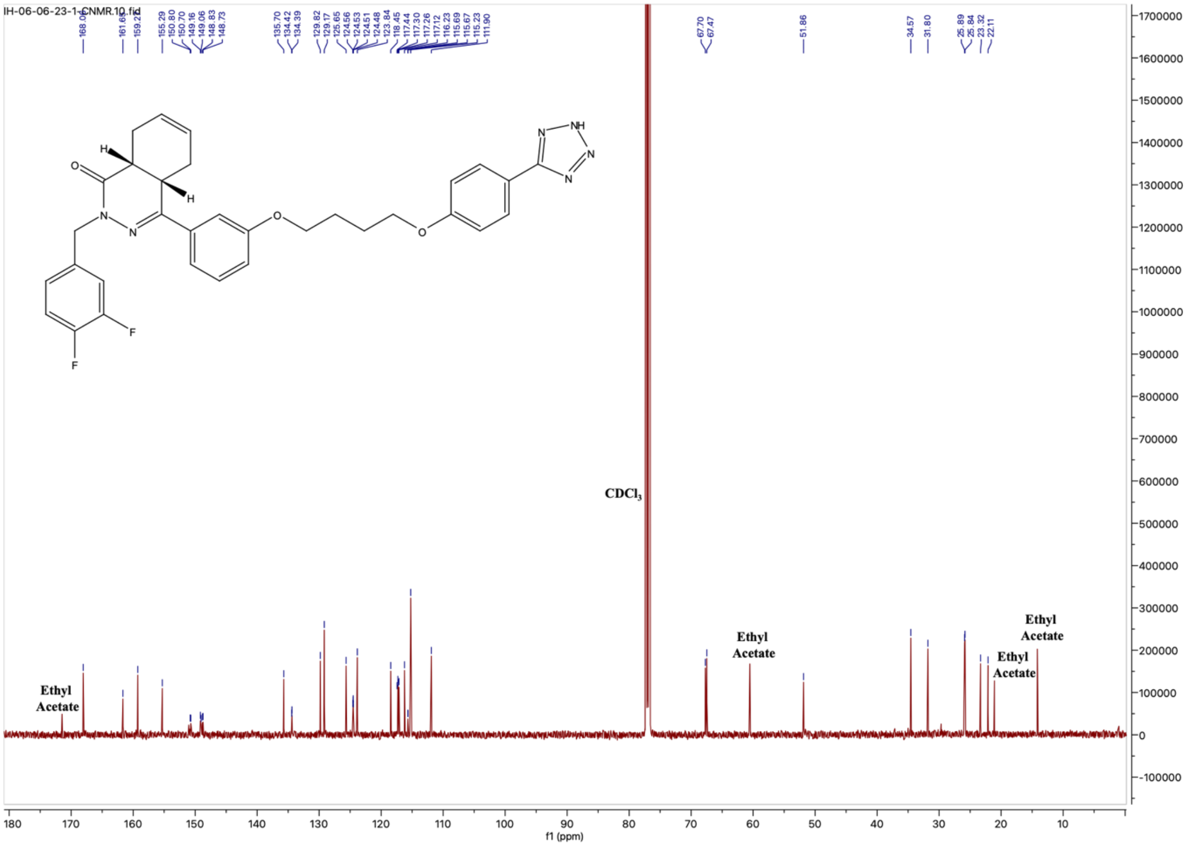

Figure S11: ^{13}CNMR for compound **7**.

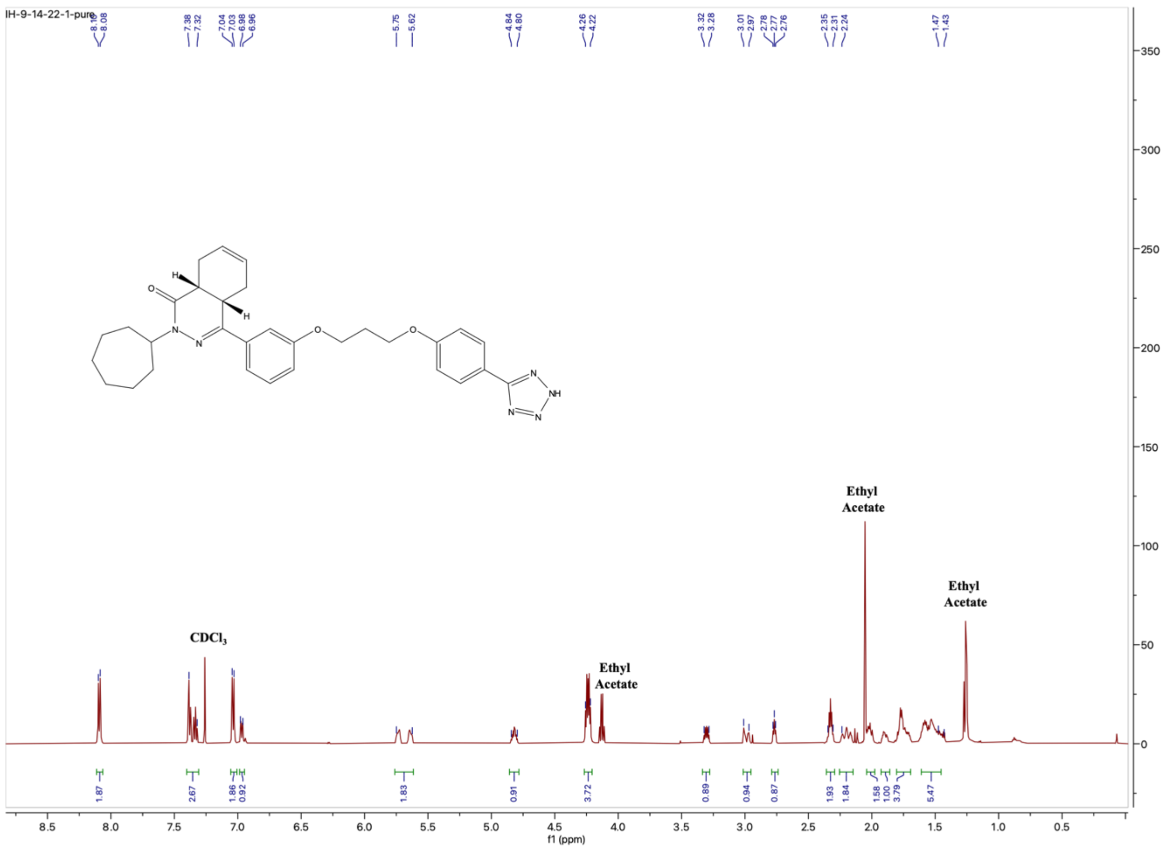

Figure S12: ^{1}HNMR for compound **8**.

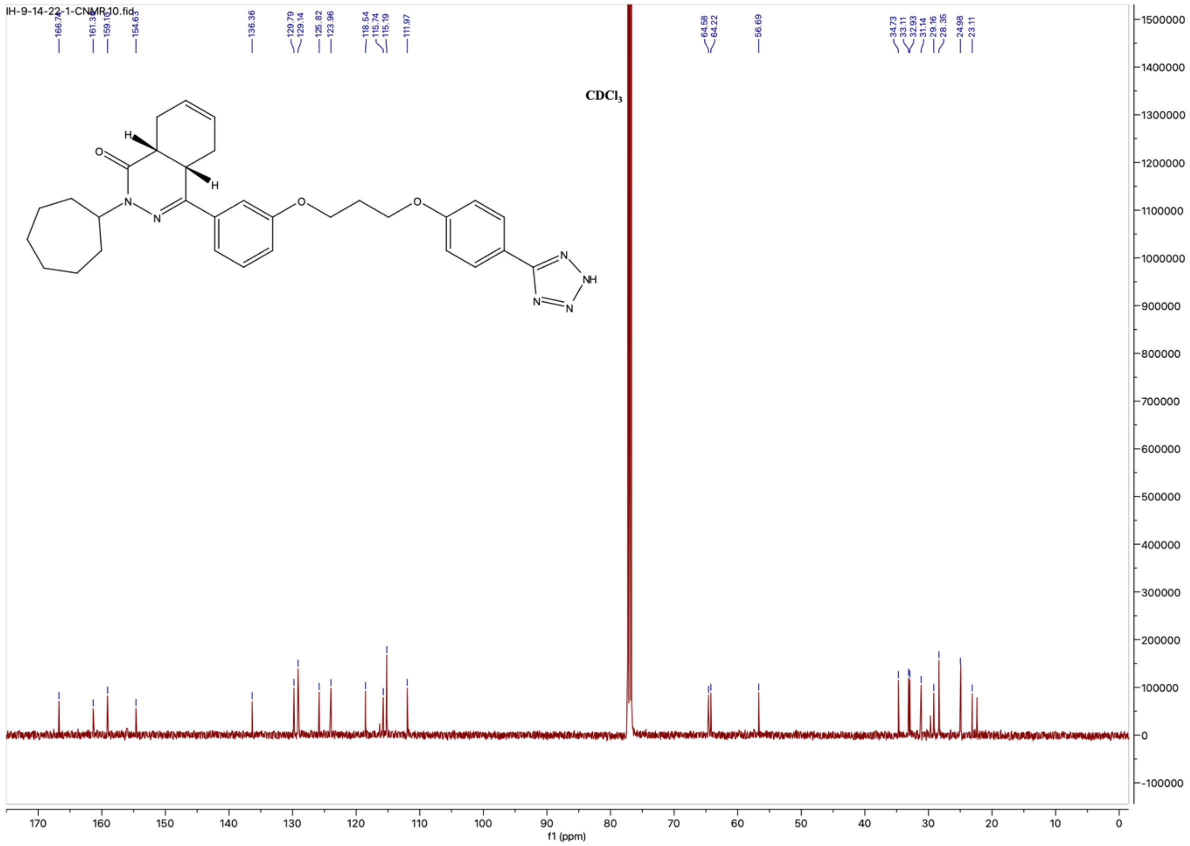

Figure S13: ^{13}CNMR for compound **8**.

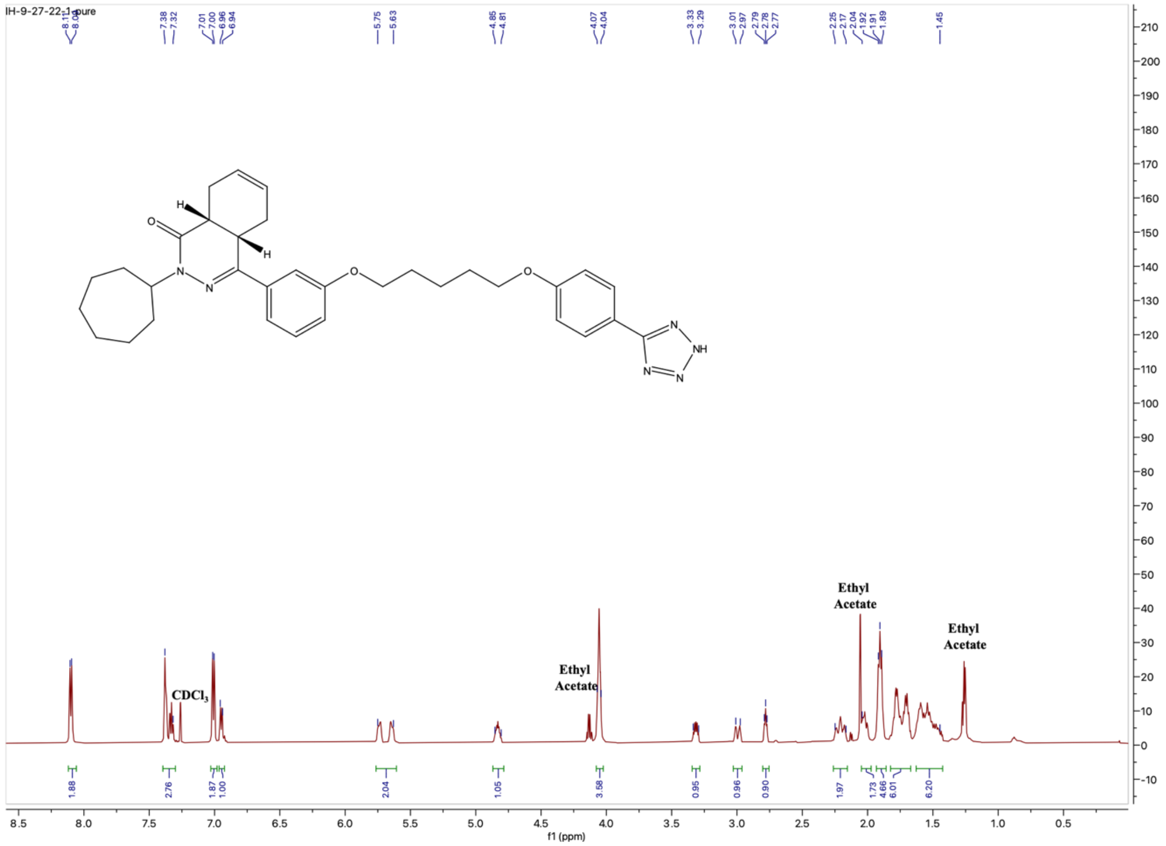

Figure S14: ^{1}HNMR for compound **9**.

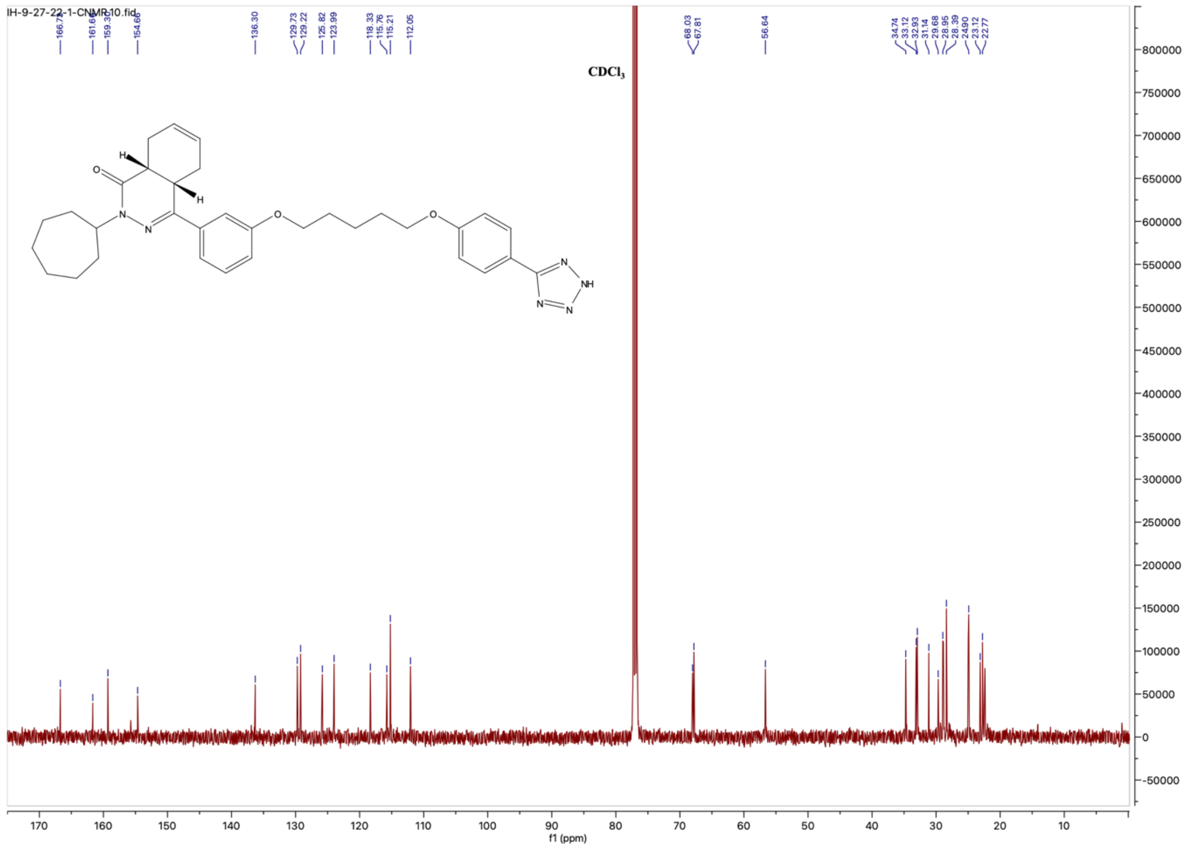

Figure S15: ^{13}CNMR for compound **9**.

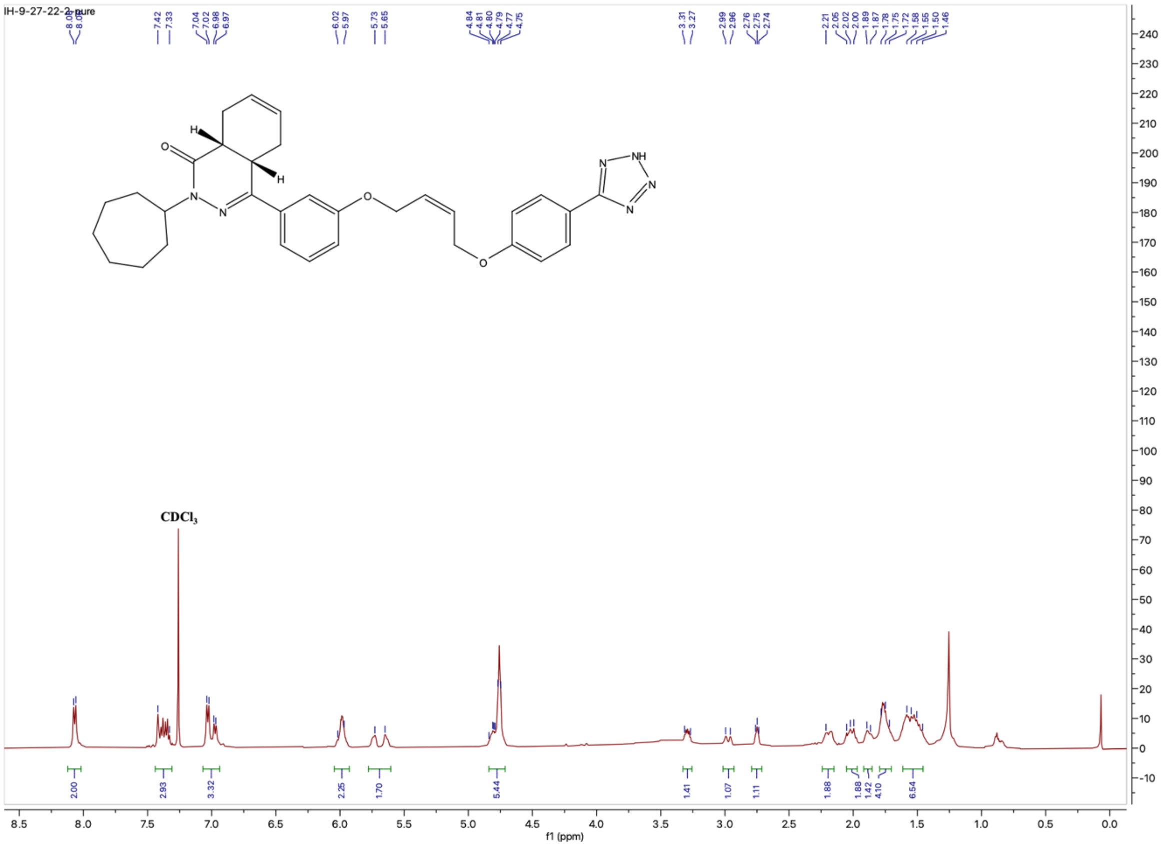

Figure S16: ^{1}HNMR for compound **10**.

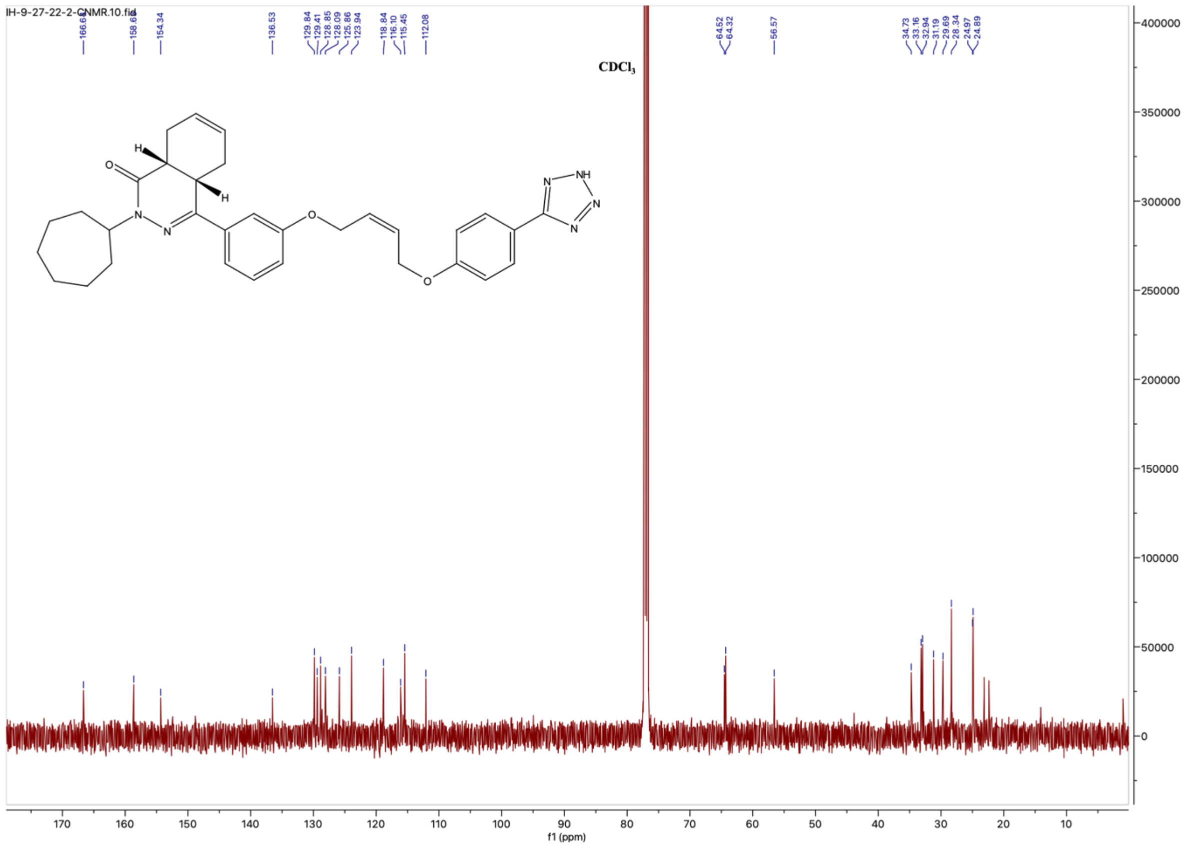

Figure S17: ^{13}CNMR for compound **10**.

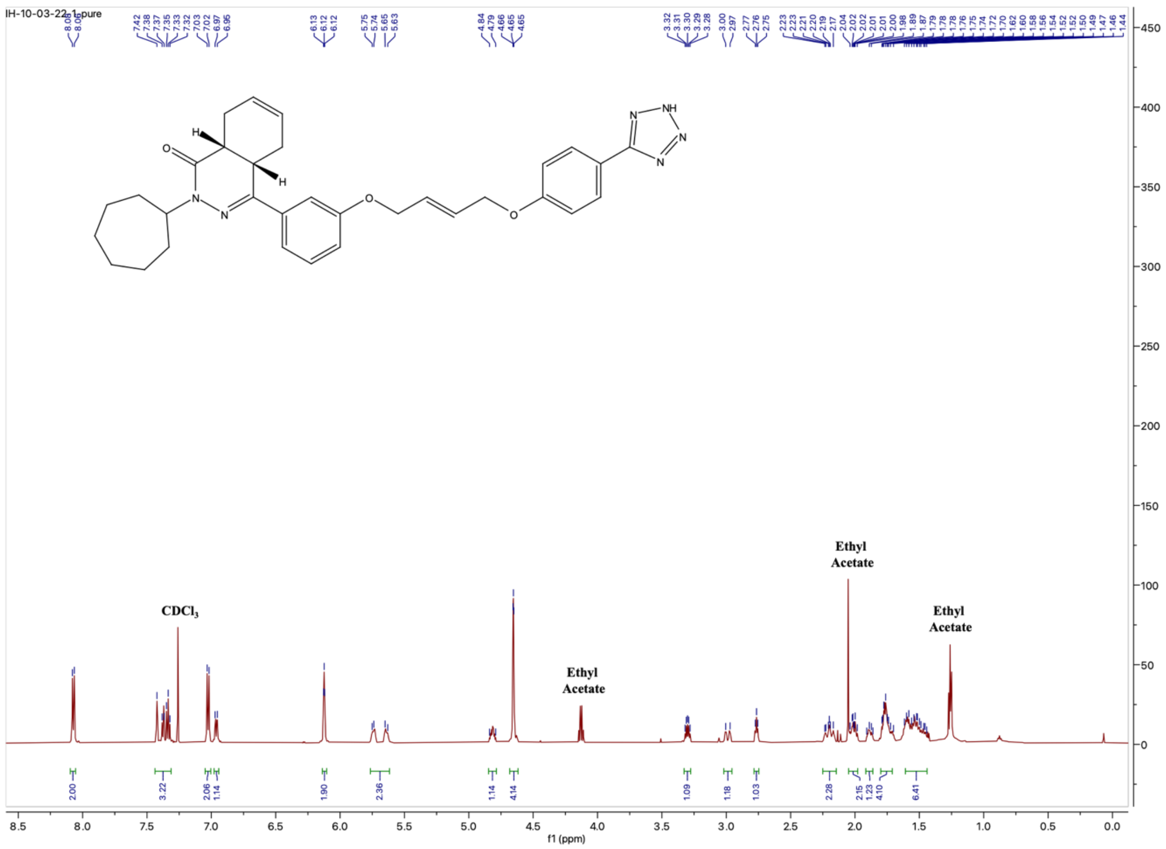

Figure S18: ^{1}HNMR for compound **11**.

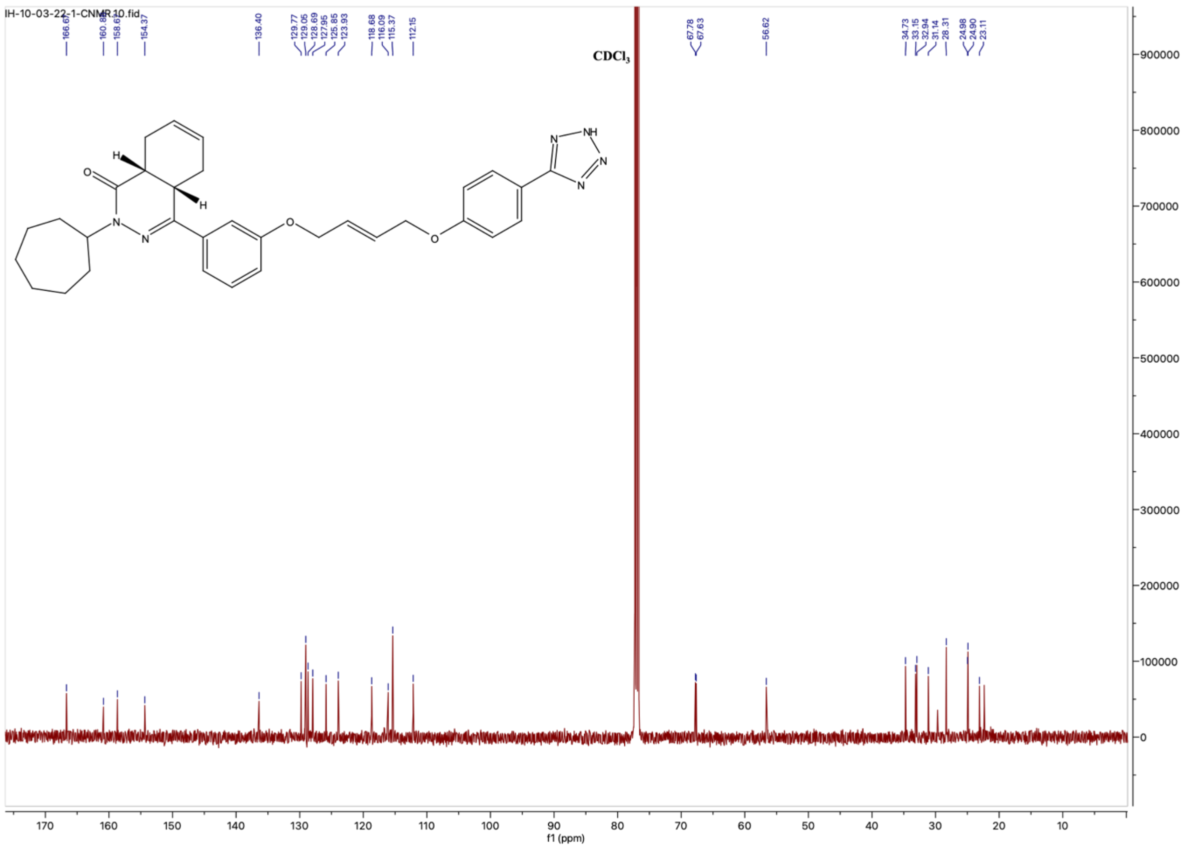

Figure S19: ^{13}CNMR for compound **11**.

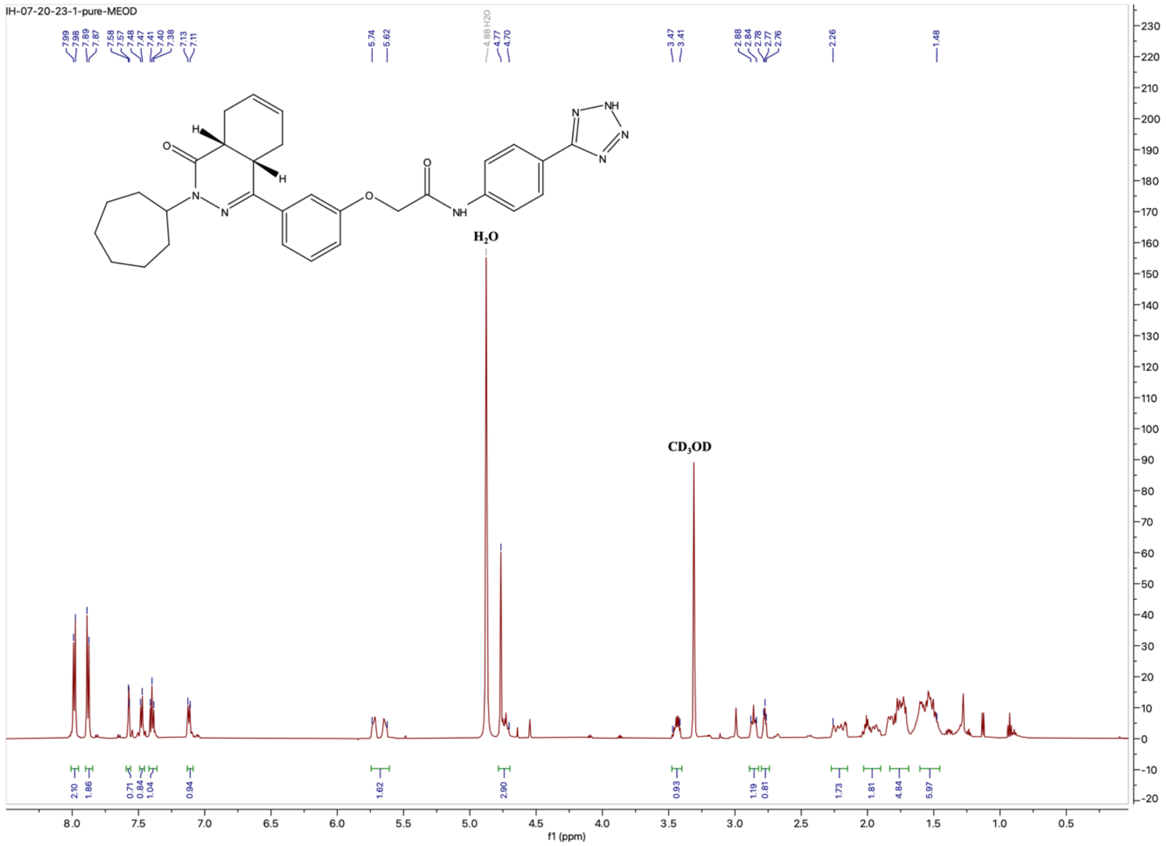

Figure S20: ^{1}HNMR for compound **12**.

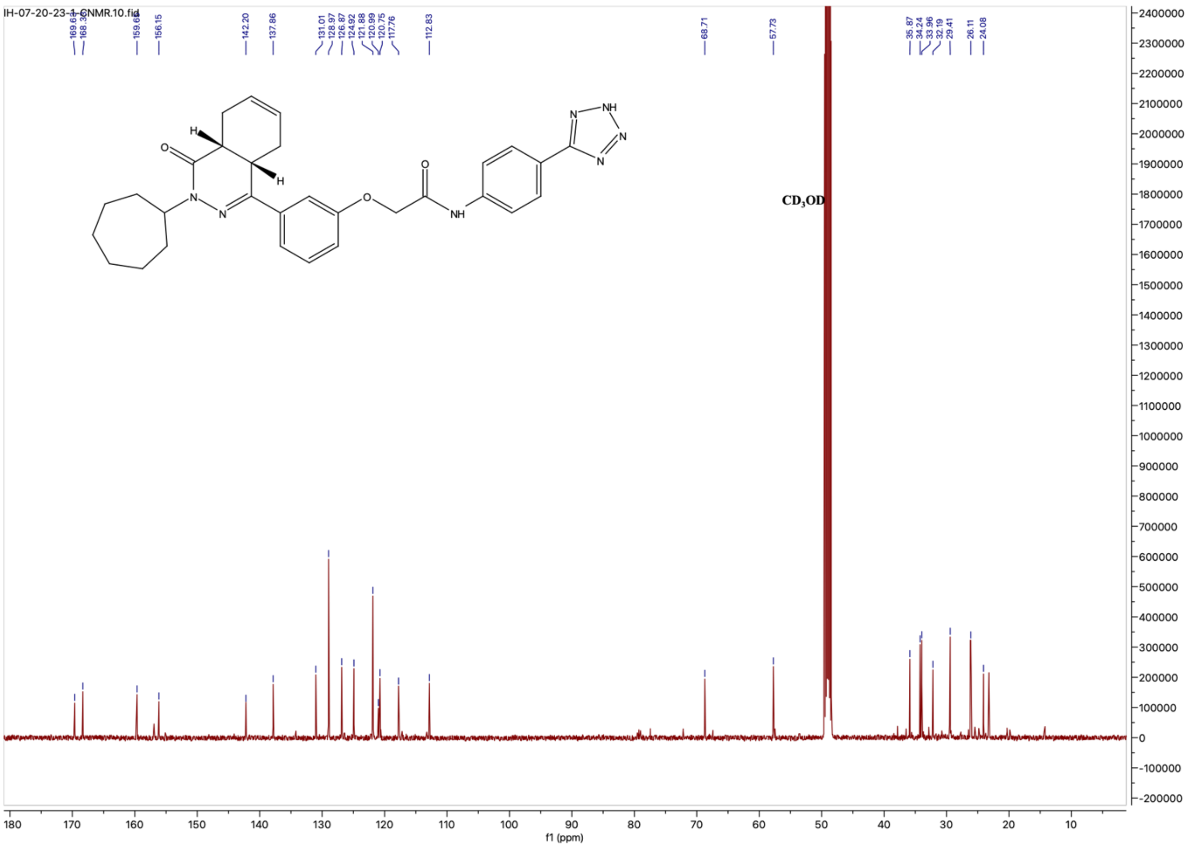

Figure S21: **13**CNMR for compound **12**.

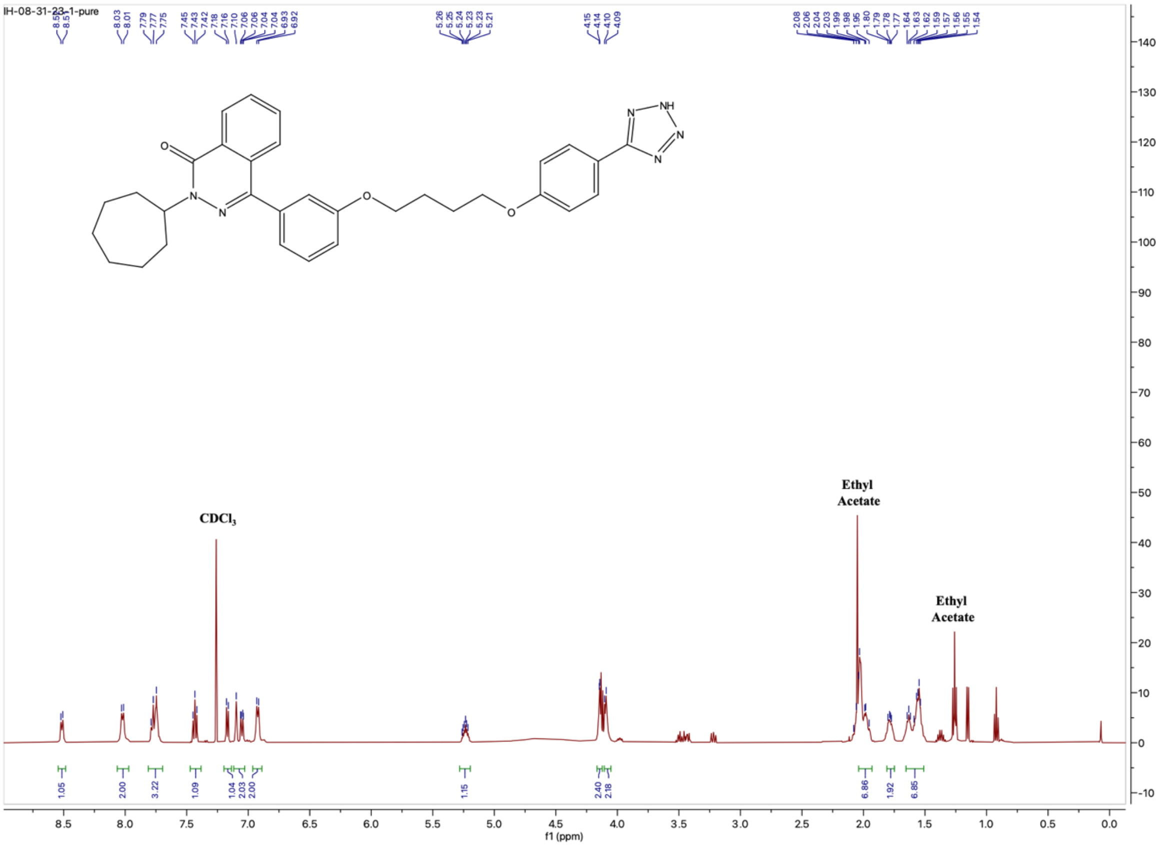

Figure S22: ^{1}HNMR for compound **13**.

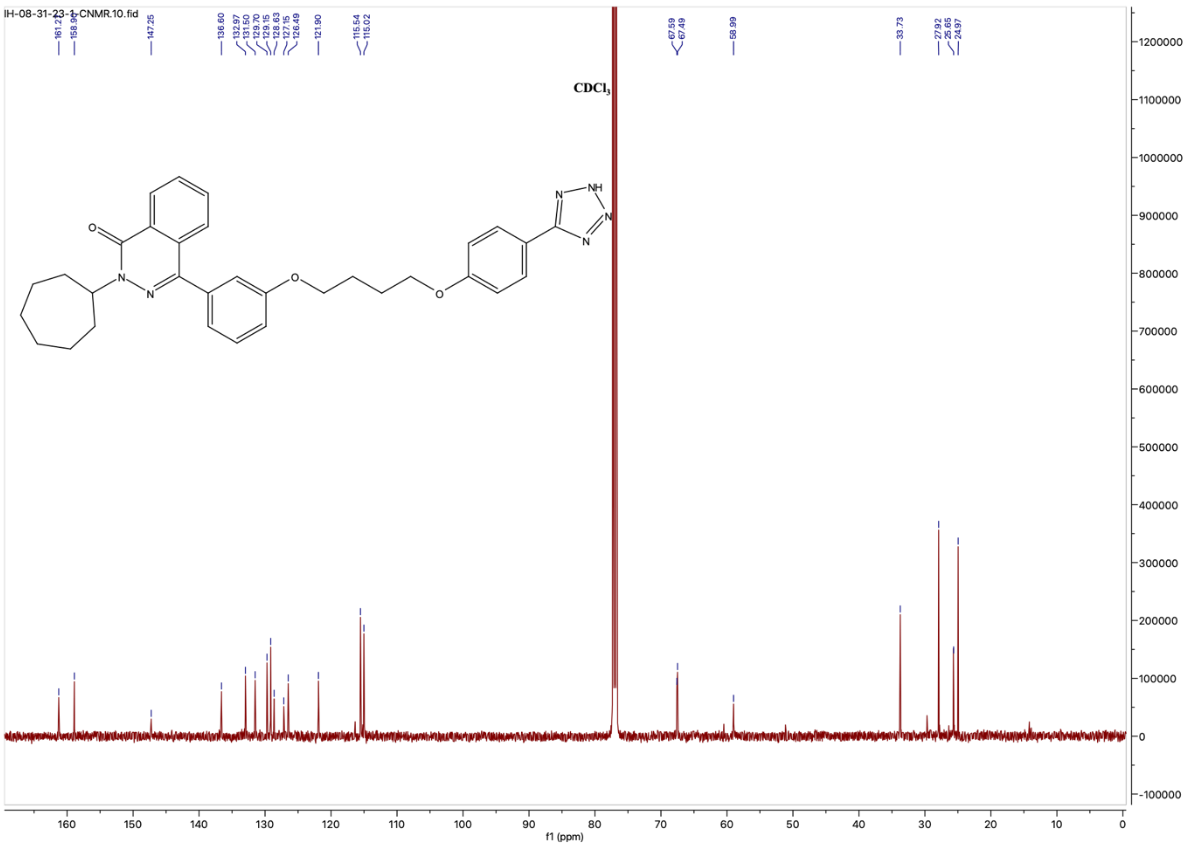

Figure S23: **13**CNMR for compound **13**.

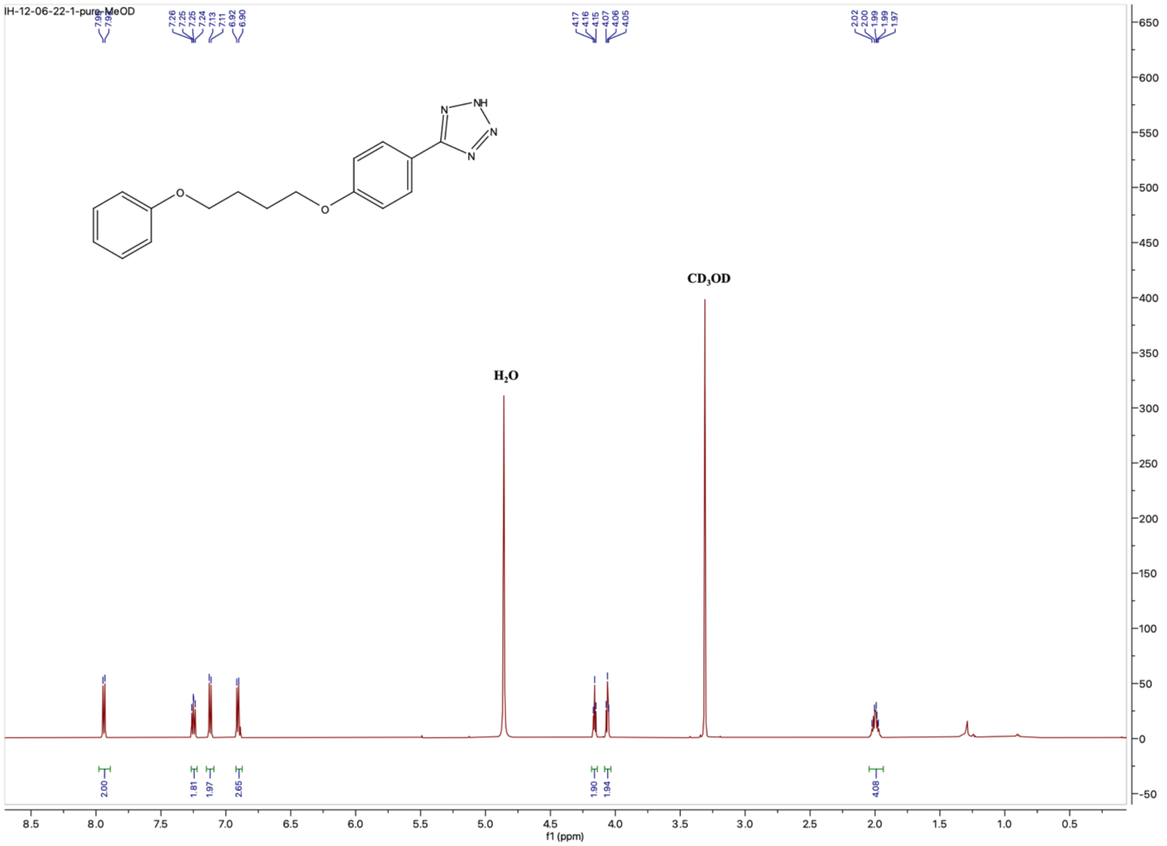

Figure S24: ^{1}HNMR for compound **14**.

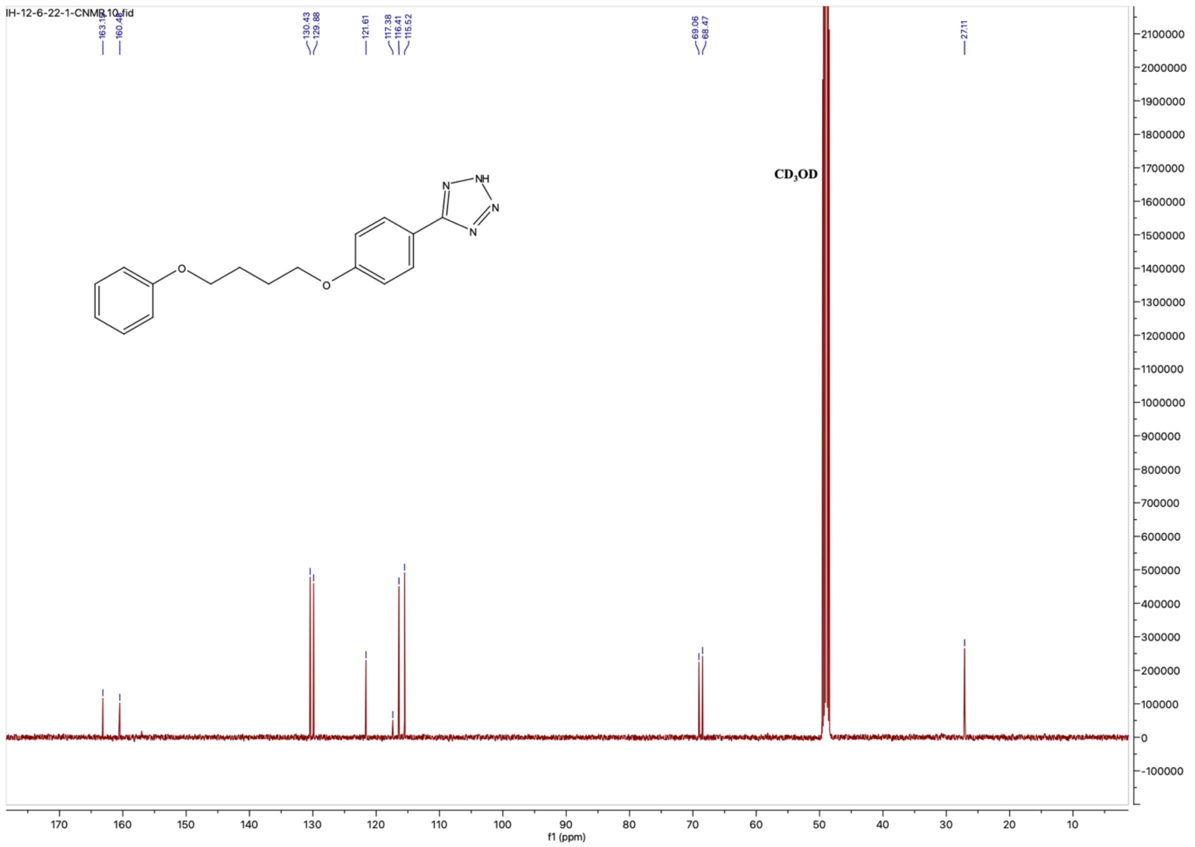

Figure S25: **13**CNMR for compound **14**.

9. References

1) Henry, S. P.; Fernandez, T. J.; Anand, J. P.; Griggs, N. W.; Traynor, J. R.; Mosberg, H. I. Structural Simplification of a Tetrahydroquinoline-Core Peptidomimetic μ-Opioid Receptor (MOR) Agonist/δ-Opioid Receptor (DOR) Antagonist Produces Improved Metabolic Stability. *J. Med. Chem.* **2019**, *62* (8), 4142-4157. DOI: 10.1021/acs.jmedchem.9b00219.
2) Van der Mey, M.; Hatzelmann, A.; Van Klink, G. P. M.; Van der Laan, I. J.; Sterk, G. J.; Thibaut, U.; Ulrich, W. R.; Timmerman, H. Novel Selective PDE4 Inhibitors. 2. Synthesis and Structure−Activity Relationships of 4-Aryl-Substituted cis-Tetra- and cis-Hexahydrophthalazinones. *J. Med. Chem.* **2001**, *44* (16), 2523-2535. DOI: 10.1021/jm010838c.
3) Swami, P.; Mali, M.; Dhulshette, B.; Ghosh, S. Total Synthesis of Thiocladospolide A and Its C2-Epimer. *Synthesis*. **2022**, *54* (03), 683-688. DOI: 10.1055/a-1652-3714.
4) Kiran, Y. B.; Wakamatsu, H.; Natori, Y.; Takahata, H.; Yoshimura, Y. Design and Synthesis of a Nucleoside and a Phosphonate Analogue Constructed on a Branched-threo-Tetrofuranose Skeleton. *Tetrahedron Lett.* **2013,** *54* (30), 3949-3952. DOI: 10.1016/j.tetlet.2013.05.062.
5) Yu, S.; Liu, Y.; Zhang, Z.; Zhang, J.; Zhao, G. Design, Synthesis, and Biological Evaluation of Novel 2,3-Indolinedione Derivatives against Mantle Cell Lymphoma. *Bioorg. Med. Chem*. **2019**, *27* (15), 3319-3327. DOI: 10.1016/j.bmc.2019.06.009.
6) Waterhouse, A.; Bertoni, M.; Bienert, S.; Studer, G.; Tauriello, G.; Gumienny, R.; Heer, F. T.; de Beer, T. A. P.; Rempfer, C.; Bordoli, L.; Lepore, R.; Schwede, T. SWISS-MODEL: Homology Modelling of Protein Structures and Complexes. *Nucleic Acids Res.* **2018**, *46,* W296– W303. DOI: 10.1093/nar/gky427.
7) Morris, G. M.; Huey, R.; Lindstrom, W.; Sanner, M. F.; Belew, R. K.; Goodsell, D. S.; Olson, A. J. AutoDock4 and AutoDockTools4: Automated docking with selective receptor flexibility. *J. Comput. Chem.* **2009**, *30*, 2785– 2791, DOI: 10.1002/jcc.21256.
